# Interferon-λ drives renal fibrosis by coordinating epithelial–fibroblast crosstalk

**DOI:** 10.1084/jem.20251858

**Published:** 2026-07-06

**Authors:** Yunfeng Zhou, Ying Zhang, Miao Zhu, Chenghui Liao, Weie Li, Jie Wang, Tie Chen, Yuan Cheng, Peter Staeheli, Liang Ye

**Affiliations:** 1 Guangdong Provincial Key Laboratory of Infection Immunity and Inflammation, Department of Physiology and Immunology, School of Basic Medical Sciences, International Cancer Center, Shenzhen University Medical School, Shenzhen University, Shenzhen, China; 2 Guangdong Key Laboratory for Biomedical Measurements and Ultrasound Imaging, National Regional Key Technology Engineering Laboratory for Medical Ultrasound, School of Biomedical Engineering, Shenzhen University Medical School, Shenzhen University, Shenzhen, China; 3 Department of Geriatric Medicine, Shenzhen University General Hospital, Shenzhen, China; 4 School of Pharmacy, Shenzhen University Medical School, Shenzhen University, Shenzhen, China; 5 Department of Nephrology, Shenzhen Second People’s Hospital, First Affiliated Hospital of Shenzhen University, Shenzhen, China; 6 Institute of Virology, Medical Center University of Freiburg, Freiburg, Germany

## Abstract

Renal fibrosis is a critical step in chronic kidney disease (CKD) progression, but fibrosis induction is still not understood well. We found that IFN-λ is a profibrotic factor that is upregulated in fibrotic human and mouse kidneys. IFN-λ receptor deficiency ameliorated renal fibrosis in mice while exogenous IFN-λ exacerbated disease, establishing a detrimental role of IFN-λ signaling in renal fibrosis. Mechanistically, we found that IFN-λ promotes fibrosis by preferentially acting on renal fibroblasts, inducing their activation and migration through ERK/JNK-dependent synthesis of TGF-β and activation of the TGF-β-SMAD2/3 signaling pathway. Renal tubular epithelial cell (TEC)-derived IFN-λ induced by RIG-I/MAVS signaling emerged as a critical driver of renal fibroblast activation and fibrogenesis. Importantly, neutralizing antibodies against IFN-λ strongly attenuated renal fibrosis in mice. Thus, the renal TEC–IFN-λ–fibroblast axis is a previously unrecognized pathway of renal fibrosis induction that represents an attractive novel target for mitigating CKD progression.

## Teaser

This study identifies a new mechanism driving kidney fibrosis. Researchers reveal that IFN-λ, produced by renal tubular epithelial cells, directly activates renal fibroblasts to cause fibrogenesis. Blocking IFN-λ signaling can delay progression of renal fibrosis.

## Graphical Abstract

**Figure:**
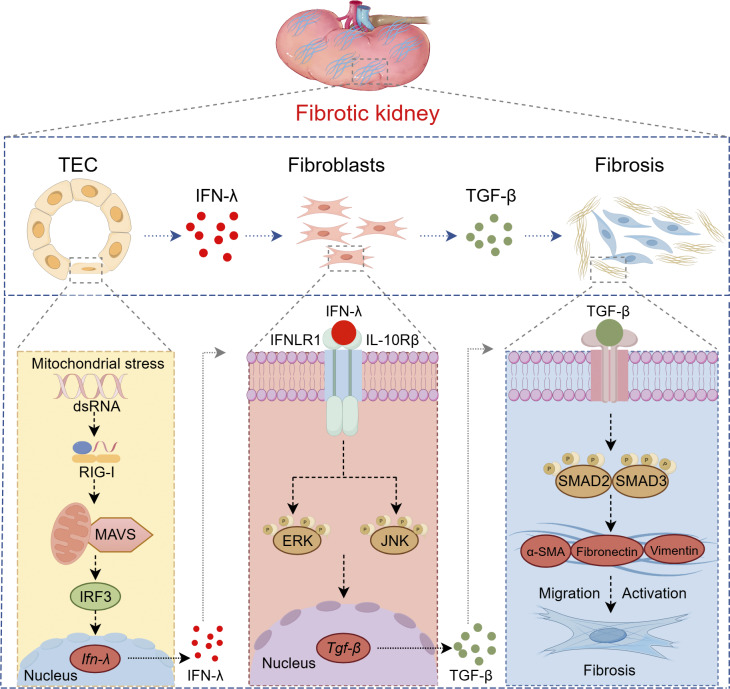

## Introduction

Renal fibrosis is a common pathological manifestation of chronic kidney disease (CKD), affecting ∼10% of the worldwide population and imposing a substantial burden on human health (Glassock et al., 2017; Levin et al., 2024; Mills et al., 2015). Persistent progression of renal fibrosis leads to irreversible deterioration of renal function, serving as a key driver of end-stage renal disease development (Ruiz-Ortega et al., 2020). Current therapeutic approaches are largely aimed at retarding renal fibrosis, which typically indicates poor prognosis and often necessitates long-term dependence on dialysis or kidney transplantation (Huang et al., 2023; Ruiz-Ortega et al., 2020). Solving this problem requires extensive research efforts and the development of innovative therapeutic strategies, both of which have become critical priorities.

Increasing evidence reveals that the development and progression of renal fibrosis involves a variety of intricate cellular and molecular cross talk mechanisms, with renal tubular epithelial cells (TECs) and fibroblasts playing pivotal roles (Abbad et al., 2025; Liu, 2011; Ruiz-Ortega et al., 2020). During renal fibrosis, fibroblasts undergo activation and migration while losing their original phenotype, presenting myofibroblast characteristics that actively drive fibrotic progression (Kendall and Feghali-Bostwick, 2014; Liu, 2011). The enhanced expression of profibrotic signature genes in fibroblasts, including α-smooth muscle actin (α-SMA), vimentin, fibronectin, and collagens, serves as a hallmark of advancing fibrosis (Kendall and Feghali-Bostwick, 2014; Ruiz-Ortega et al., 2020). The upregulation of these genes demonstrates a strong correlation with progressive renal function decline (Kendall and Feghali-Bostwick, 2014). Therefore, targeted regulation of these profibrotic genes might provide innovative strategies and approaches for therapeutic intervention in renal fibrosis. Renal TECs are considered primary targets of cellular injury (Liu, 2011). Following renal damage and incomplete epithelial repair, renal TECs initiate activation and migration of adjacent renal fibroblasts, ultimately promoting renal fibrotic development (Li et al., 2019; Liu et al., 2023; Widjaja et al., 2022). Therefore, identifying key molecular mediators in renal TECs that participate in renal fibroblast activation may lead to novel therapeutic approaches for preventing renal fibrosis.

Type III IFN, also known as IFN-λ, is the newest member of the IFN family and mediates potent antiviral effects through engagement of a unique heterodimeric receptor complex comprising IFN-λ receptor 1 (IFNLR1) and IL-10 receptor β chain (IL-10Rβ) (Ye et al., 2019b). IFN-λ interacts with IFNLR1 and activates the downstream JAK–STAT pathway, similar to type I IFN (IFN-α/β) (Ye et al., 2019b). The IFN-α/β receptor exhibits ubiquitous expression in nearly all nucleated cells, while IFNLR1 displays a tissue-restricted distribution pattern, with predominant localization to epithelial cells and select immune populations, particularly murine neutrophils and human B cells (Broggi et al., 2017; Espinosa et al., 2017; Ye et al., 2019a; Ye et al., 2019b). This compartmentalized receptor architecture confers IFN-λ signaling with distinct functional specialization, enabling targeted activity at the mucosal barrier while minimizing systemic activation (Mahlakoiv et al., 2015; Sommereyns et al., 2008; Wang et al., 2025; Ye et al., 2019a; Ye et al., 2019b). Emerging evidence now extends IFN-λ's biological relevance beyond antiviral immunity, revealing its pathological role in noninfectious diseases, including autoimmune diseases and chronic inflammatory pathologies (Goel et al., 2020; Goel et al., 2021; Jena et al., 2024; Lazear et al., 2015). In these contexts, IFN-λ signaling appears to impair epithelial regeneration while amplifying inflammatory cascades (Broggi et al., 2020; Jena et al., 2024; Major et al., 2020; Pires et al., 2024). Recent clinical studies identified an association between IFN-λ polymorphisms and renal fibrosis susceptibility (Kwak et al., 2022), yet the mechanistic contribution of IFN-λ to fibrogenesis remains poorly delineated.

In this study, we identified IFN-λ as a fibrosis-associated cytokine dynamically upregulated in both murine and human fibrotic kidneys, with expression levels tightly correlated to disease progression. Genetic ablation of IFN-λ signaling markedly attenuated fibrosis in unilateral ureteral obstruction (UUO) models, whereas exogenous IFN-λ administration exacerbated fibrotic outcomes, establishing its causal role in renal fibrogenesis. Mechanistically, IFN-λ directly activates the ERK/JNK signaling pathway in renal fibroblasts, thereby inducing the secretion of TGF-β. IFN-λ further potentiates the TGF-β–Smad2/3 pathway, which in turn establishes a positive feedback loop that amplifies profibrotic signaling. Notably, renal TECs emerge as the primary source of IFN-λ, with production driven by retinoic acid–inducible gene I (RIG-I)/mitochondrial antiviral-signaling protein (MAVS)-dependent sensing of tubular damage signals. This renal TEC–derived IFN-λ facilitates fibroblast migration and activation through paracrine mechanisms, establishing a renal TEC–IFN-λ–fibroblast axis that is central to fibrotic progression. Genetic or pharmacological blockade of IFN-λ signaling attenuated renal fibrotic progression in mice, highlighting its therapeutic potential in this animal model. Therefore, this study provides comprehensive evidence that IFN-λ serves as a central pathogenic mediator of renal fibrosis through a renal TEC-fibroblast cross talk axis, thereby identifying previously unrecognized therapeutic targets to intercept profibrotic cascades.

## Results

### IFN-λ is highly upregulated in murine and human fibrotic kidneys

**Figure 1.**
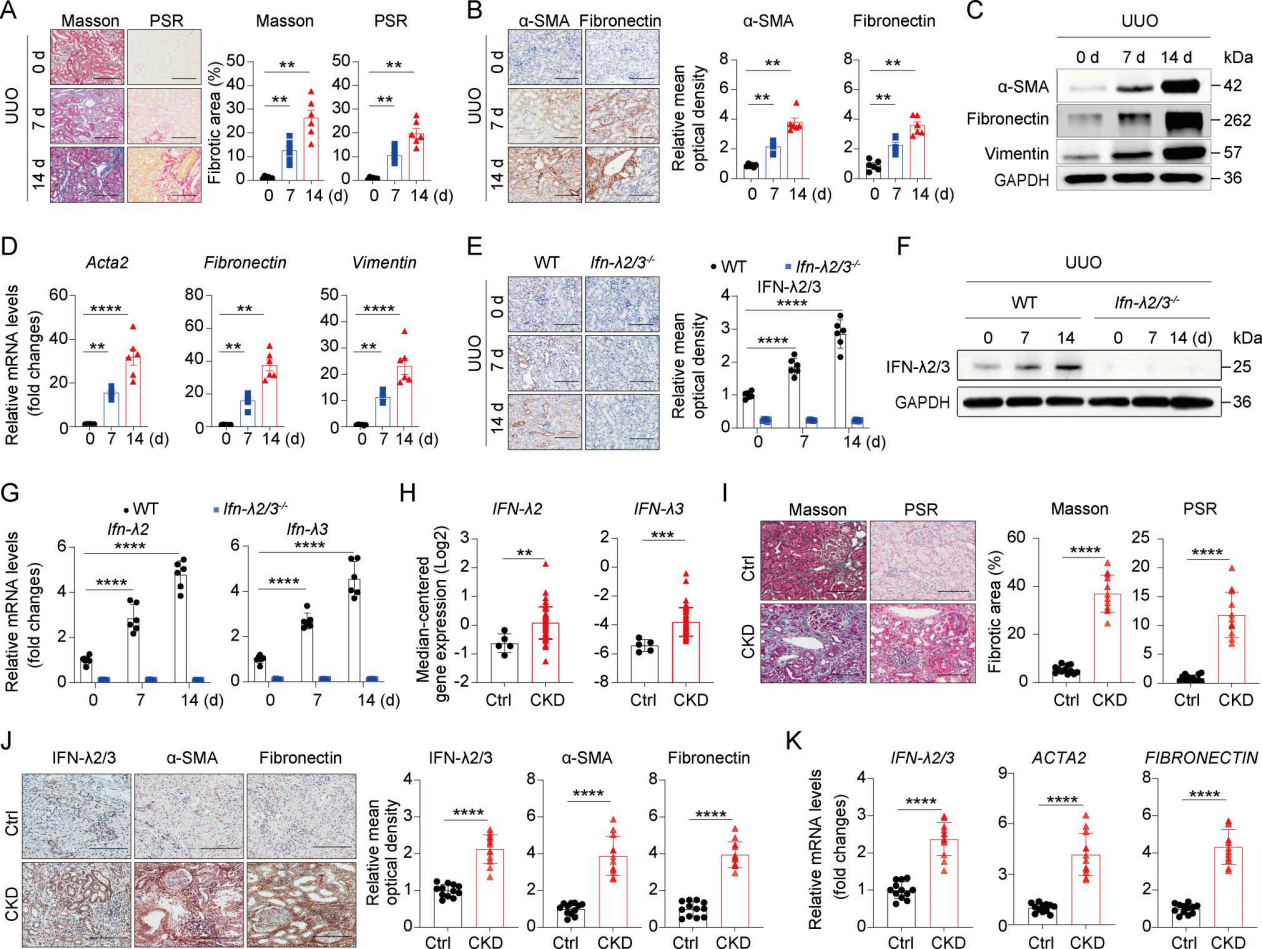
**IFN-λ is upregulated in fibrotic murine and human kidneys. (A–D)** WT mice underwent UUO surgery, and kidneys were harvested at 0, 7, and 14 days (*n* = 6 per group). **(A)** Representative images and quantitative analysis of fibrotic areas with Masson’s trichrome and PSR staining in mouse kidneys. **(B)** Representative immunohistochemical images and quantitative analysis of α-SMA and fibronectin relative mean OD (MOD) in the kidneys of UUO mice. **(C)** Western blot analysis showing α-SMA, fibronectin, and vimentin protein levels in kidneys of UUO mice at days 0, 7, and 14. **(D)** RT-qPCR analysis of *Acta2,* fibronectin*,* and vimentin mRNA levels in kidneys of UUO mice. Relative fold changes in target gene mRNA expression were normalized to *Gapdh*. **(E–G)** WT and *Ifn-λ2/3*^*–/–*^ mice following UUO, and kidneys were harvested at 0, 7, and 14 days (*n* = 6 per group). **(E)** Representative immunohistochemical images and quantitative analysis of IFN-λ2/3 relative MOD in the kidneys of UUO-treated WT and *Ifn-λ2/3*^*–/–*^ mice. **(F and G)** Western blot (F) and RT-qPCR (G) analysis showing IFN-λ2/3 protein and mRNA levels in kidneys of UUO mice. Relative fold changes in target gene mRNA expression were normalized to *Gapdh*. **(H)** The expression levels of *IFN-λ2* and *IFN-λ3* in control (Ctrl) and CKD patients from large cohorts by the Nephroseq version 5.0 database. **(I)** Kidney sections from control (Ctrl) and CKD patients were stained with Masson’s trichrome and PSR. The percentage of fibrotic area was quantified (*n* = 12 per group). **(J)** Immunohistochemical images and quantitation of IFN-λ2/3, α-SMA, and fibronectin MOD in Ctrl and CKD patients (*n* = 12 per group). **(K)** RT-qPCR analysis of *IFN-λ*2/3, *ACTA2*, and fibronectin mRNA levels in kidneys of Ctrl and CKD patients (*n* = 12 per group). Relative fold changes in target gene mRNA expression were normalized to *ACTB*. Scale bars = 50 μm (A, B, E, I, and J). Data in A–G and I–K are represented as individual data points pooled from two independent experiments. Data in H were adapted from Nephroseq version 5.0 database. Data are presented as mean ± SEM. **P < 0.01, ***P < 0.001, ****P < 0.0001, by one-way ANOVA with Tukey’s multiple-comparison test (A–D), two-way ANOVA with Tukey’s multiple-comparison test (E–G), or unpaired two-tailed Student’s *t* test (H–K). Source data are available for this figure: SourceData F1.

**Figure S1.**
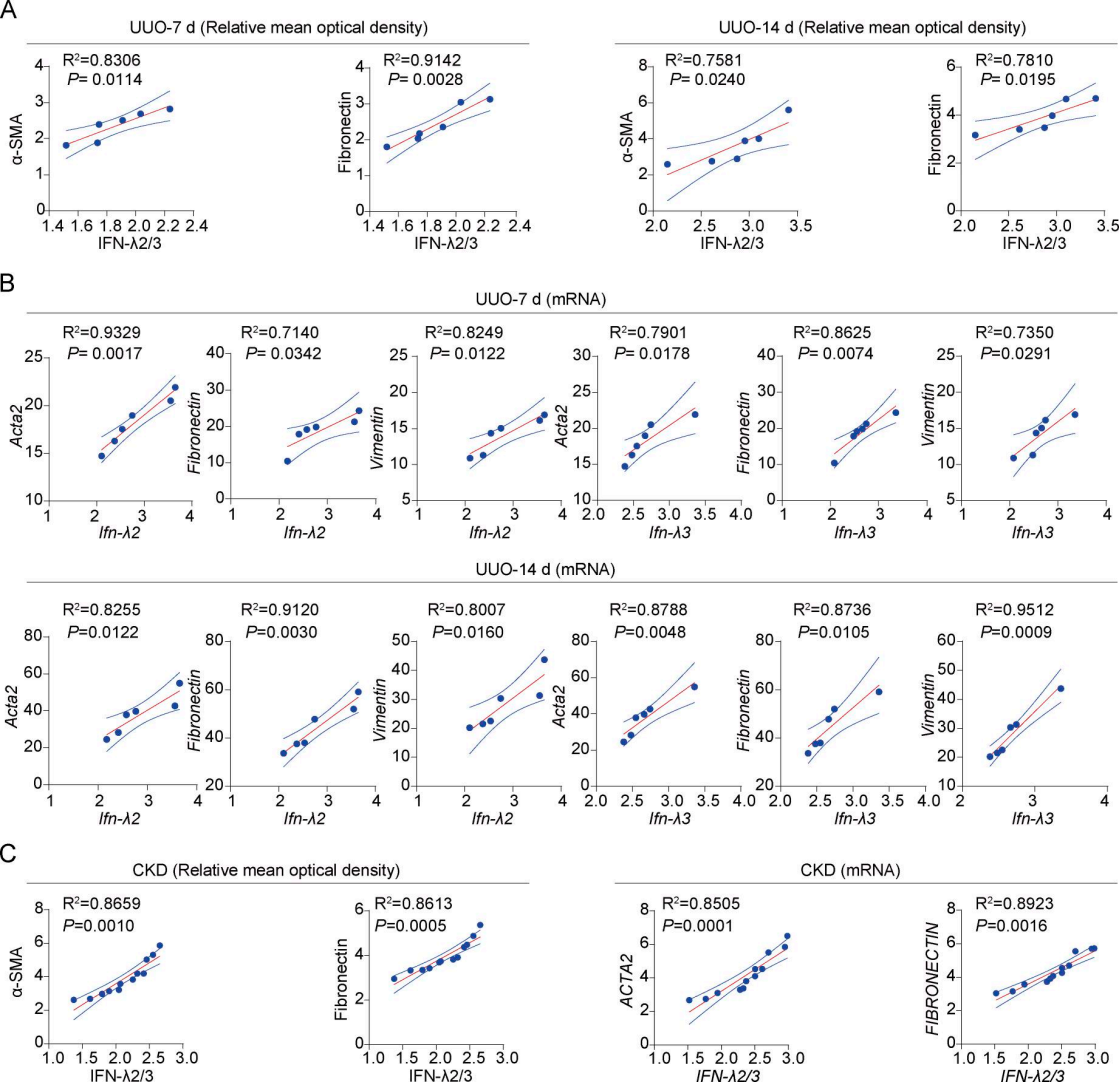
**Correlation between renal IFN-λ expression and renal fibrosis. (A)** Pearson correlation analysis between IFN-λ2/3, α-SMA, and fibronectin relative mean OD (MOD) levels in kidneys from UUO mice at days 7 and 14 after surgery (*n* = 6). **(B)** Pearson correlation analysis of *Ifn-λ2/3* mRNA levels with *Acta2*, fibronectin, and vimentin mRNA levels in kidneys from UUO mice at days 7 and 14 after surgery (*n* = 6). **(C)** Pearson correlation analysis of IFN-λ2/3, α-SMA, and fibronectin MOD values in kidney samples from CKD patients (*n* = 12) (left panel). Pearson correlation analysis of *IFN-λ2/3* mRNA levels with *ACTA2* and fibronectin mRNA levels in kidney samples from CKD patients (*n* = 12) (right panel). Data are represented as individual data points pooled from two independent experiments. The correlation coefficient and corresponding P values by the Pearson correlation test.

Previous research has shown that IFN-λ polymorphisms are closely associated with susceptibility to renal fibrosis (Kwak et al., 2022). To investigate a potential role of IFN-λ in the pathogenesis of renal fibrosis, we first measured IFN-λ levels in fibrotic kidneys of mice with UUO. WT mice exhibited the expected renal fibrosis phenotypes, evidenced by increased collagen deposition and fibrotic lesions through Masson’s trichrome and Picrosirius red (PSR) staining at days 7 and 14 after UUO operation (Fig. 1 A). Consistent with this, the expression of profibrotic markers (α-SMA [encoded by *Acta2*], fibronectin, and vimentin) was progressively upregulated at the same time points, as analyzed by immunohistochemistry (IHC) (Fig. 1 B), western blot (Fig. 1 C), and real-time quantitative PCR (RT-qPCR) (Fig. 1 D). Interestingly, IHC and western blot analysis of fibrotic kidneys showed a marked time-dependent increase in IFN-λ2/3 staining in WT mice, which was undetectable in IFN-λ2/3 double knockout (*Ifn-λ2/3*^*–/–*^) mice (Fig. 1, E and F). Complementary RT-qPCR analyses confirmed progressive expression of IFN-λ2 and IFN-λ3 at transcript levels in fibrotic mouse kidneys from WT mice but not *Ifn-λ2/3*^*–/–*^ mice (Fig. 1 G). Notably, expression of IFN-λ2/3 positively correlated with α-SMA and fibronectin staining in renal fibrotic mice (Fig. S1 A). Pearson correlation analysis demonstrated strong positive associations between *Ifn-λ2* and *Ifn-λ3* mRNA and pro-fibrosis factors *Acta2*, fibronectin, and vimentin mRNA in fibrotic kidneys at days 7 and 14 following UUO (Fig. S1 B), suggesting that IFN-λ is associated with the progression of renal fibrosis in mice.

Next, we assessed the expression of IFN-λ in kidneys with fibrosis from patients with CKD. Transcriptomic analysis from the Nephroseq public database revealed that *IFN-λ2* and *IFN-λ3* were significantly upregulated in kidney from CKD compared with controls (Fig. 1 H). To validate this, we collected kidney tissue samples from CKD patients. Healthy areas of renal tissue from patients who underwent tumor nephrectomies or renal cystectomies served as controls. As expected, Masson’s trichrome and PSR staining assay showed more prominent collagen deposition and fibrotic lesions in renal tissues from CKD groups compared with controls (Fig. 1 I). Interestingly, IHC and RT-qPCR analyses demonstrated concurrent upregulation of IFN-λ2/3, α-SMA, and fibronectin at the protein and transcriptional levels in CKD kidneys (Fig. 1, J and K). Moreover, IFN-λ2/3 protein and mRNA expression exhibited strong positive correlations with both α-SMA and fibronectin (Fig. S1 C). Thus, IFN-λ expression was substantially increased in fibrotic renal tissues from both mice and humans. In addition, IFN-λ expression correlated with fibrosis progression, indicating that IFN-λ is a profibrotic factor.

### IFN-λ promotes renal fibrogenesis in mice

**Figure 2.**
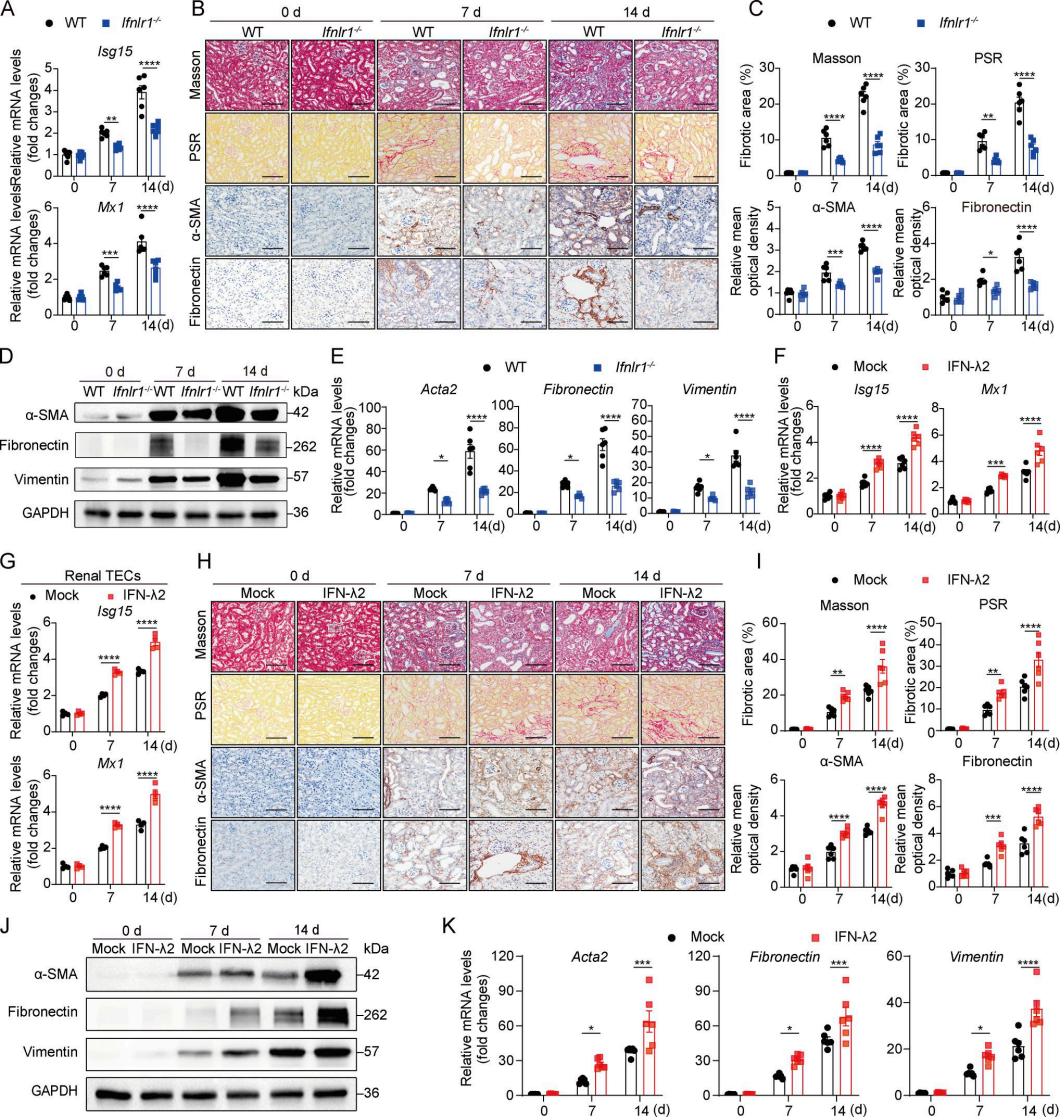
**IFN-λ triggers renal fibrosis in mice. (A–E)** WT and *Ifnlr1*^*−/−*^ mice underwent UUO surgery, and kidneys were harvested at days 0, 7, and 14 after surgery (*n* = 6). **(A)** RT-qPCR analysis for *Isg15* and *Mx1* mRNA levels in kidneys of WT and *Ifnlr1*^*−/−*^ UUO mice at days 0, 7, and 14 after surgery. **(B)** Representative images showing Masson’s trichrome, PSR, α-SMA, and fibronectin staining in kidneys among groups (scale bars = 50 μm). **(C)** Quantitative analysis of B revealed the percentage of fibrotic area and α-SMA and fibronectin MOD in kidneys. **(D)** Western blot examination of α-SMA, fibronectin, and vimentin protein levels in kidneys from WT and *Ifnlr1*^*−/−*^ UUO mice. GAPDH was employed as a loading control. **(E)** RT-qPCR analysis for renal *Acta2,* fibronectin*,* and vimentin mRNA levels in the indicated groups. **(F–K)** WT UUO mice were subcutaneously injected with 1 μg of IFN-λ2 on days −1, 1, 3, 5, 7, 9, 11, and 13. Kidneys and renal TECs were collected at days 0, 7, and 14 after UUO (*n* = 6). **(F)** RT-qPCR analysis for *Isg15* and *Mx1* mRNA levels in kidneys of IFN-λ2–treated UUO mice. **(G)** RT-qPCR analysis for *Isg15* and *Mx1* mRNA levels in renal TECs at indicated time points. **(H)** Representative images of Masson’s trichrome, PSR, α-SMA, and fibronectin staining in kidney sections at indicated time points (scale bars = 50 μm). **(I)** Quantitative analysis of H revealed differences in fibrotic area, α-SMA, and fibronectin MOD in the kidneys among groups. **(J)** Western blot assessment of α-SMA, fibronectin, and vimentin protein levels in UUO kidneys in the presence or absence of IFN-λ2 treatment. **(K)** RT-qPCR analysis of renal *Acta2,* fibronectin*,* and vimentin mRNA levels. Data in A, C, E–G, I, and K are pooled from two independent experiments. Data in B, D, H, and J are representative of two independent experiments. Data are shown as mean ± SEM. *P < 0.05, **P < 0.01, ***P < 0.001, ****P < 0.0001, by two-way ANOVA with Tukey’s multiple-comparison test. MOD, mean OD. Source data are available for this figure: SourceData F2.

To verify the involvement of IFN-λ in renal fibrosis, we subjected WT and IFN-λ receptor–deficient (*Ifnlr1*^*−/−*^) mice to UUO or sham surgery. To confirm the functional efficacy of the IFN-λ receptor in this model, we first evaluated IFN-stimulated gene (ISG) expression. *Ifnlr1*^*−/−*^ UUO mice exhibited significantly reduced ISG (*Isg15* and *Mx1*) levels, confirming impaired IFN-λ signaling (Fig. 2 A). IHC analysis through Masson’s trichrome and PSR staining revealed considerable collagen deposition and fibrotic lesions in renal sections of WT mice at 7 and 14 days after UUO. In contrast, mice lacking functional IFN-λ receptors exhibited significantly attenuated renal fibrosis (Fig. 2, B and C). Consistent with the reduced fibrotic response observed in *Ifnlr1*^*−/−*^ UUO mice, the absence of IFN-λ signaling also mitigated the expression of fibrotic markers, including α-SMA and fibronectin, after UUO (Fig. 2, B and C). Western blot analysis of whole-kidney tissue lysates further demonstrated lower protein levels of α-SMA, fibronectin, and vimentin in *Ifnlr1*^*−/−*^ mice compared with WT mice following UUO (Fig. 2 D). Moreover, RT-qPCR analysis showed that transcript levels of fibrotic markers *Acta2*, fibronectin, and vimentin were significantly decreased in *Ifnlr1*^*−/−*^ mice relative to WT controls at both 7 and 14 days after UUO surgery (Fig. 2 E). These results demonstrated that loss of IFN-λ signaling delays the development of UUO-induced fibrosis in mice.

To further delineate the role of IFN-λ in renal fibrosis, we treated UUO mice with exogenous IFN-λ2 by subcutaneous injection. We first confirmed the potent *in vivo* bioactivity of IFN-λ2 by observing significant upregulation of ISGs (*Isg15* and *Mx1*) in both kidney tissue and renal TECs (Fig. 2, F and G). IHC analysis demonstrated that IFN-λ2 accelerates renal fibrosis by increasing fibrotic areas (Masson’s trichrome and PSR staining) and enhancing the expression of fibrotic markers (α-SMA and fibronectin staining) on days 7 and 14 after UUO (Fig. 2, H and I). In addition, fibrosis-associated markers were increased in kidney tissues from IFN-λ2–treated UUO mice at both protein and mRNA levels (Fig. 2, J and K). Taken together, these results indicated that IFN-λ accelerates progression of UUO-induced renal fibrosis.

### IFN-λ acts on renal fibroblasts and drives renal fibrosis in mice

**Figure 3.**
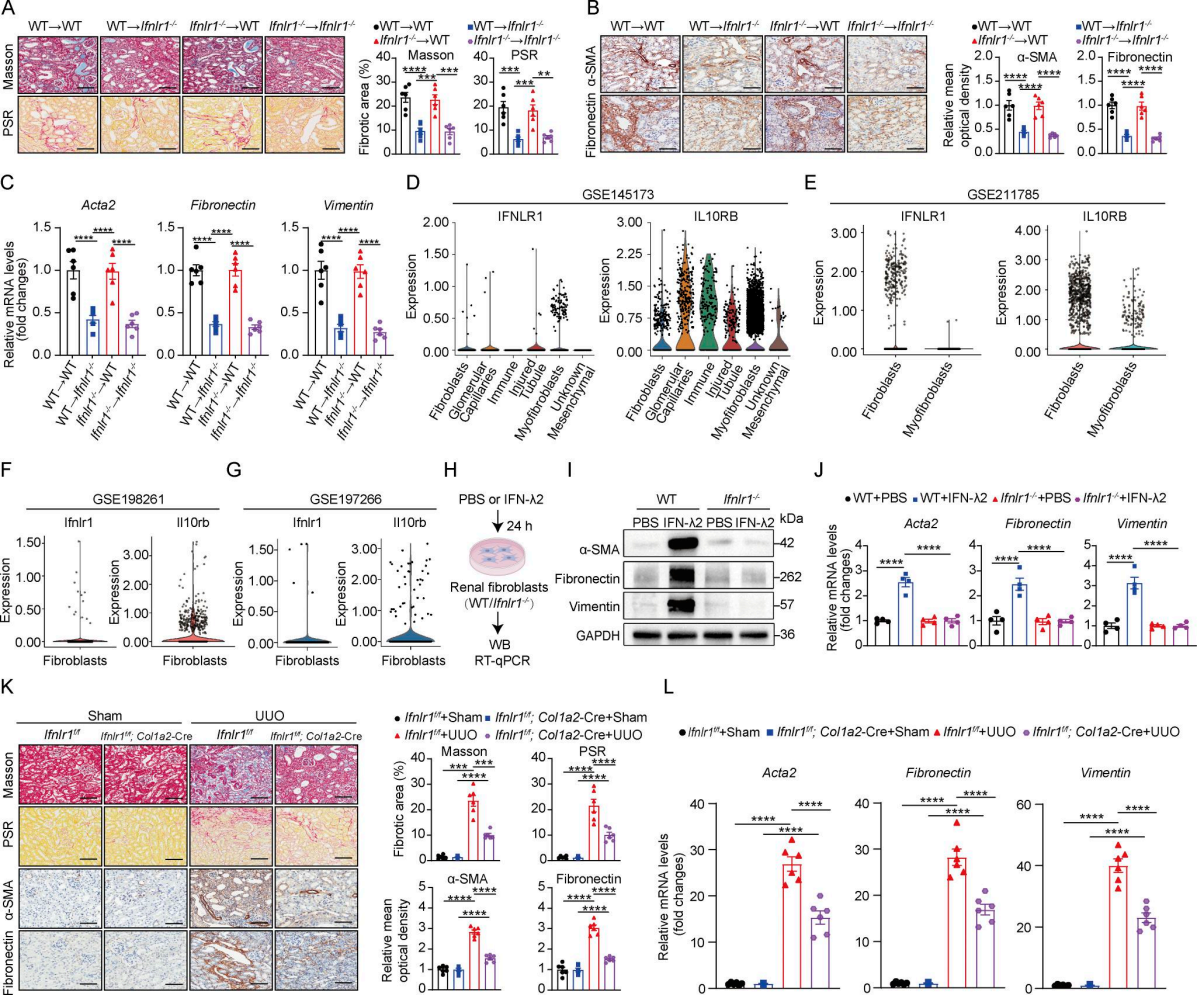
**IFN-λ induces renal fibrosis in mice by directly targeting renal fibroblasts. (A–C)** WT and *Ifnlr1*^*−/−*^ recipient mice were lethally irradiated before being reconstituted with donor BM cells from either WT or *Ifnlr1*^*−/−*^ donor mice to generate BM chimeric mice. Mice were subjected to a UUO surgery, and kidneys were harvested 7 days later (*n* = 6). **(A)** Representative images and quantitative analysis of fibrotic area in kidneys of BM chimeric mice after UUO surgery, assessed by Masson’s trichrome and PSR staining (scale bars = 50 μm). **(B)** Representative IHC images and corresponding quantification of α-SMA and fibronectin MOD in kidneys of BM chimeric mice following UUO (scale bars = 50 μm). **(C)** mRNA levels of *Acta2*, fibronectin*,* and vimentin in the kidneys were measured by RT-qPCR. **(D and E)** scRNA-seq analysis of human kidneys with renal fibrosis (GSE145173 and GSE211785) revealed *IFNLR1* and *IL10RB* expression in kidney cell populations. **(F and G)** scRNA-seq analysis from UUO mouse kidneys (GSE198261 and GSE197266) demonstrated *Ifnlr1* and *Il10rβ* expression in renal fibroblasts. **(H)** Primary renal fibroblasts isolated from WT and *Ifnlr1*^*−/−*^ mice were treated with or without 100 ng/ml IFN-λ2 for 24 h, followed by western blot and RT-qPCR analyses. **(I)** Western blot analysis of α-SMA, fibronectin, and vimentin protein expression levels in renal fibroblasts. GAPDH was employed as a loading control. **(J)** mRNA levels of *Acta2,* fibronectin*,* and vimentin in renal fibroblasts were measured by RT-qPCR (*n* = 4). **(K and L)** Fibroblast-specific *Ifnlr1* knockout mice (*Ifnlr1*^*f/f*^; *Col1a2-*Cre) and their littermate control mice (*Ifnlr1*^*f/f*^) were subjected to sham or UUO surgery, and kidneys were collected on day 7 (*n* = 6). **(K)** Representative images and quantitative analysis for Masson’s trichrome, PSR, α-SMA, and fibronectin staining in kidneys of *Ifnlr1*^*f/f*^ and *Ifnlr1*^*f/f*^; *Col1a2-*Cre mice following sham or UUO surgery (scale bars = 50 μm). **(L)** RT-qPCR analysis of *Acta2*, fibronectin*,* and vimentin mRNA levels in kidneys of *Ifnlr1*^*f/f*^ and *Ifnlr1*^*f/f*^; *Col1a2-*Cre after sham or UUO surgery. Data in A–C and J–L are pooled from two independent experiments. Data in H and I are representative of two independent experiments. Data in D–G were adapted from indicated scRNA-seq dataset. Data are presented as mean ± SEM. **P < 0.01, ***P < 0.001, ****P < 0.0001, by two-way ANOVA with Tukey’s multiple-comparison test. MOD, mean OD. Source data are available for this figure: SourceData F3.

To identify IFN-λ–responsive cells in renal fibrosis, we first generated bone marrow (BM) chimeric mice by adoptively transferring BM cells from WT and *Ifnlr1*^*–/–*^ donor mice into irradiated WT and *Ifnlr1*^*−/−*^ recipient mice. 7 wk after BM transplantation, chimeric mice were subjected to UUO. WT mice reconstituted with *Ifnlr1*^*−/−*^ BM had identical fibrosis in obstructed kidneys as WT mice transplanted with WT BM, evidenced by Masson’s trichrome, PSR, α-SMA, and fibronectin staining (Fig. 3, A and B) and fibrotic-associated gene expression (*Acta2*, fibronectin*,* and vimentin) (Fig. 3 C). Furthermore, there was no significant difference in the extent of renal fibrosis in *Ifnlr1*^*–/– *^mice transplanted with WT or *Ifnlr1*^*−/−*^ BM (Fig. 3, A–C). These data suggested that the impact of IFN-λ on renal fibrosis is not due to effects on hematopoietic cells but rather to a localized effect within the kidney.

**Figure S2.**
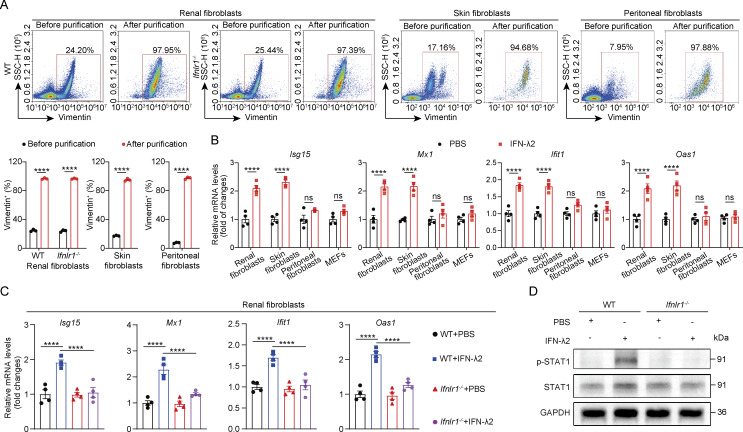
**IFN-λ acts on renal fibroblasts by activating the STAT1 signaling pathway. (A)** Flow cytometric analysis of the proportions of renal fibroblasts, skin fibroblasts, and peritoneal fibroblasts before and after purification from different mouse tissues. Fibroblasts were gated on forward and side scatters, then single cell, live cells, and finally vimentin^+^ cells (*n* = 3). **(B)** Primary renal fibroblasts, skin fibroblasts, peritoneal fibroblasts, and MEFs were treated with 100 ng/ml of IFN-λ2 or PBS for 24 h. RT-qPCR was performed to determine *Isg15*, *Mx1*, *Ifit1*, and *Oas1* mRNA levels (*n* = 4). **(C and D)** Primary renal fibroblasts isolated from WT and *Ifnlr1*^*−/−*^ mice were treated with 100 ng/ml of IFN-λ2 or PBS for 24 h or 1 h for RT-qPCR and western blot analysis, respectively (*n* = 4). **(C)** RT-qPCR was performed to determine *Isg15*, *Mx1*, *Ifit1*, and *Oas1* mRNA levels. **(D)** Western blot was used to assess STAT1 phosphorylation and protein levels. GAPDH was used as a loading control. Data are representative of two independent experiments. Data are presented as mean ± SEM. ****P < 0.0001, by unpaired two-tailed Student’s *t* test (A and B), or two-way ANOVA with Tukey’s multiple-comparison test (C and D). ns, no significant difference. Source data are available for this figure: SourceData FS2.

To identify the cellular mediators of IFN-λ signaling in the kidney, we analyzed publicly available single-cell RNA-sequencing (scRNA-seq) datasets from CKD patients and UUO mice. Analysis from two independent CKD datasets (GSE145173 and GSE211785) revealed that while the human IL-10Rβ chain is broadly expressed, IFNLR1 expression is primarily confined to distinct fibroblast subsets and a limited number of nonimmune cells in fibrotic kidney (Fig. 3, D and E). Consistently, mouse *Ifnlr1* expression was also confirmed in renal fibroblasts from UUO mice across two independent datasets (GSE198261 and GSE197266) (Fig. 3, F and G). Since fibroblasts play a central role in the development of renal fibrosis (Kendall and Feghali-Bostwick, 2014; Liu, 2011; Ruiz-Ortega et al., 2020), we sought to determine whether IFN-λ might directly activate renal fibroblasts. Such activity appeared unlikely because fibroblasts were reported to express few if any functional IFN-λ receptors (Ank et al., 2008; Lasfar et al., 2006). To determine whether kidney fibroblasts might be exceptional, we isolated high-purity primary fibroblasts from multiple mouse tissues (kidney, skin, and the peritoneum) and employed MEF cell lines for comparative functional analysis (Fig. S2 A). Interestingly, treatment of renal fibroblasts with IFN-λ2 markedly induced the expression of downstream ISGs (Fig. S2 B), demonstrating that these cells can effectively respond to IFN-λ. In contrast, IFN-λ2 did not induce ISG expression in primary peritoneal fibroblasts and in MEFs, but IFN-λ2 upregulated ISG expression in fibroblasts from skin, suggesting that IFN-λ action on fibroblasts shows a high degree of tissue specificity (Fig. S2 B). As expected, IFN-λ2–mediated induction of ISGs was completely abolished in renal fibroblasts isolated from *Ifnlr1*^*−/−*^ mice (Fig. S2 C), and IFN-λ2–mediated STAT1 activation in renal fibroblasts was IFN-λ receptor dependent (Fig. S2 D). In response to IFN-λ2 stimulation, profibrotic factors such as α-SMA (encoded by *Acta2*), fibronectin, and vimentin were more strongly upregulated at both protein and transcription levels in renal fibroblasts from WT mice compared with renal fibroblasts from *Ifnlr1*^*−/−*^ mice (Fig. 3, I and J), demonstrating that IFN-λ can readily activate renal fibroblasts *ex vitro.*

**Figure S3.**
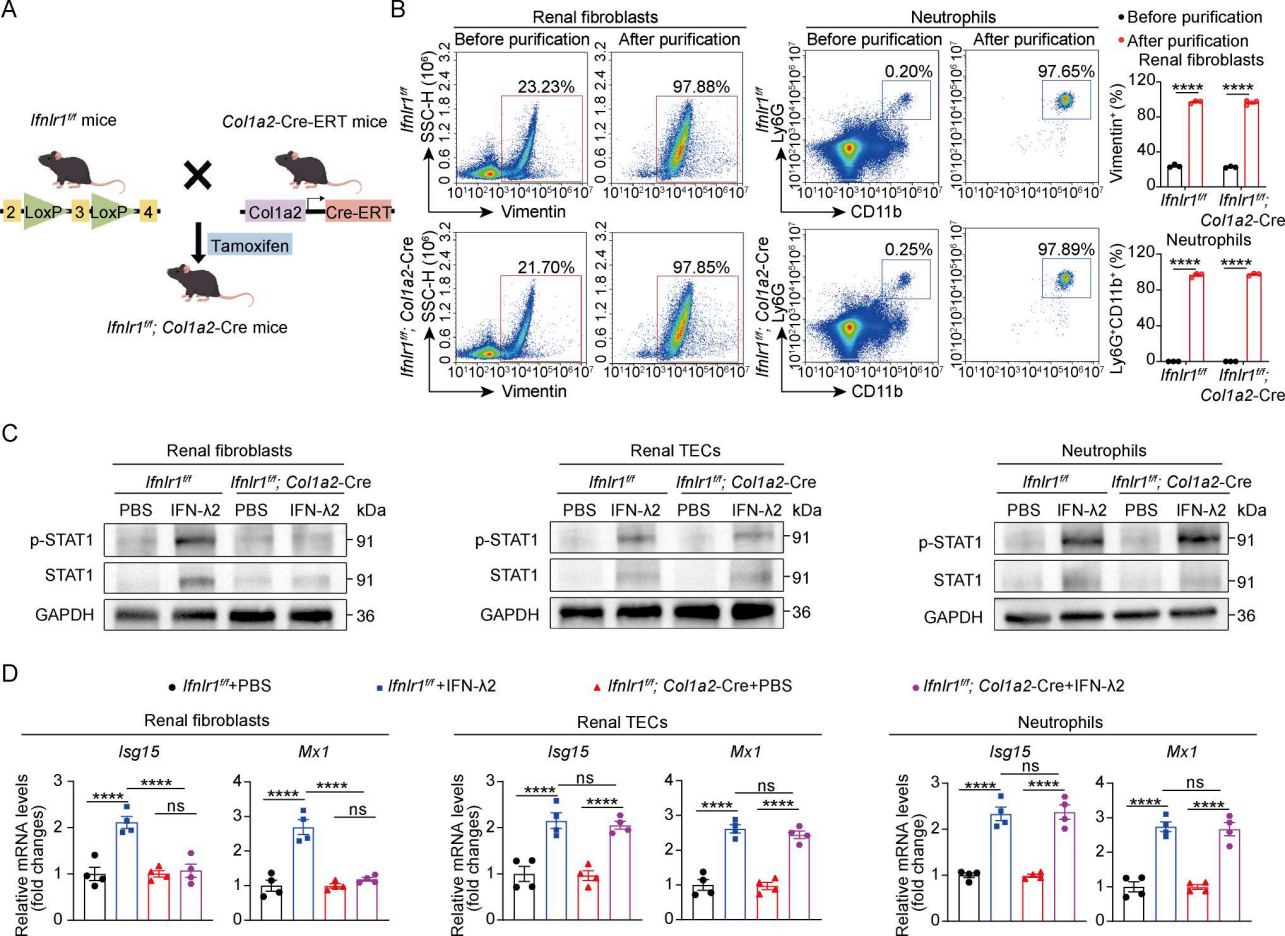
**Generation and validation of mice with conditional *Ifnlr1* deletion in fibroblasts. (A)** Strategy for generating mice with fibroblast-specific *Ifnlr1* deletion (designated *Ifnlr1*^*f/f*^; *Col1a2-*Cre). **(B)** Representative flow cytometry plots demonstrating the successful enrichment of renal vimentin^+^ fibroblasts and splenic Ly6G^+^ CD11b^+^ neutrophils from *Ifnlr1*^*f/f*^ mice and *Ifnlr1*^*f/f*^; *Col1a2-*Cre mice. Cells were gated on forward and side scatters, then single cell, live cells, and finally on vimentin^+^ fibroblasts or Ly6G^+^ CD11b^+^ neutrophils were gated (*n* = 3). **(C)** Primary renal fibroblasts, renal TECs, and splenic neutrophils isolated from *Ifnlr1*^*f/f*^ mice and *Ifnlr1*^*f/f*^; *Col1a2-*Cre mice were treated with 100 ng/ml of IFN-λ2 or PBS for 1 h. Western blot was used to assess STAT1 expression and phosphorylation. GAPDH was used as a loading control. **(D)** After 24 h of IFN-λ2 or PBS exposure, RT-qPCR was used to determine the levels of *Isg15* and *Mx1* mRNA in primary renal fibroblasts, renal TECs, and neutrophils from *Ifnlr1*^*f/f*^ and *Ifnlr1*^*f/f*^; *Col1a2-*Cre mice. *n* = 4 biologically independent samples per group. Data are representative of two independent experiments. Data are shown as mean ± SEM. ****P < 0.0001, by two-way ANOVA with Tukey’s multiple-comparison test. ns, no significant difference. Source data are available for this figure: SourceData FS3.

To test whether IFN-λ drives renal fibrosis through a direct effect on renal fibroblasts *in vivo*, we generated mice with conditional *Ifnlr1* deletion in fibroblasts (designated *Ifnlr1*^*f/f*^; *Col1a2*-Cre) by crossing *Col1a2*-Cre-ERT mice (expressing tamoxifen-inducible Cre recombinase under the control of the mouse collagen type I α 2 promoter) with mice homozygous for a floxed *Ifnlr1* allele (*Ifnlr1*^*f/f*^). To selectively induce deletion of the *Ifnlr1* gene in fibroblasts, we injected *Ifnlr1*^*f/f*^*; Col1a2*-Cre-ERT mice with tamoxifen. Littermate *Ifnlr1*^*f/f*^ mice were used as controls (Fig. S3 A). Due to the lack of commercially available antibodies against mouse IFNLR1, we treated purified renal fibroblasts and splenic neutrophils (purity >97%), as well as isolated renal TECs derived from *Ifnlr1*^*f/f*^ mice and *Ifnlr1*^*f/f*^*; Col1a2*-Cre mice with IFN-λ2 to assess downstream STAT1 activation and ISG expression (Fig. S3 B). We found that IFN-λ2 robustly induced STAT1 phosphorylation and expression of *Isg15* and *Mx1* mRNA in renal fibroblasts from *Ifnlr1*^*f/f*^ mice but failed to induce such responses in renal fibroblasts from *Ifnlr1*^*f/f*^*; Col1a2*-Cre mice (Fig. S3, C and D). In contrast, IFN-λ2 readily induced STAT1 activation and ISG expression in renal TECs and splenic neutrophils from both genotypes (Fig. S3, C and D). These results confirmed the specific ablation of IFNLR1 signaling in renal fibroblasts. In response to UUO, *Ifnlr1*^*f/f*^*; Col1a2*-Cre mice showed significantly less collagen deposition and fewer fibrotic lesions compared with *Ifnlr1*^*f/f*^ mice, as shown by Masson’s trichrome and PSR staining as well as by α-SMA and fibronectin staining (Fig. 3 K). Consistent with this finding, we noted that *Ifnlr1*^*f/f*^*; Col1a2*-Cre mice showed reduced expression of fibrosis-associated genes such as *Acta2*, fibronectin, and vimentin in renal tissue on day 7 after UUO compared with *Ifnlr1*^*f/f*^ mice (Fig. 3 L). These results showed that IFN-λ signaling in fibroblasts is an important driver of renal fibrosis in UUO mice.

### IFN-λ promotes TGF-β synthesis and downstream SMAD2/3 activation in renal fibroblasts

**Figure 4.**
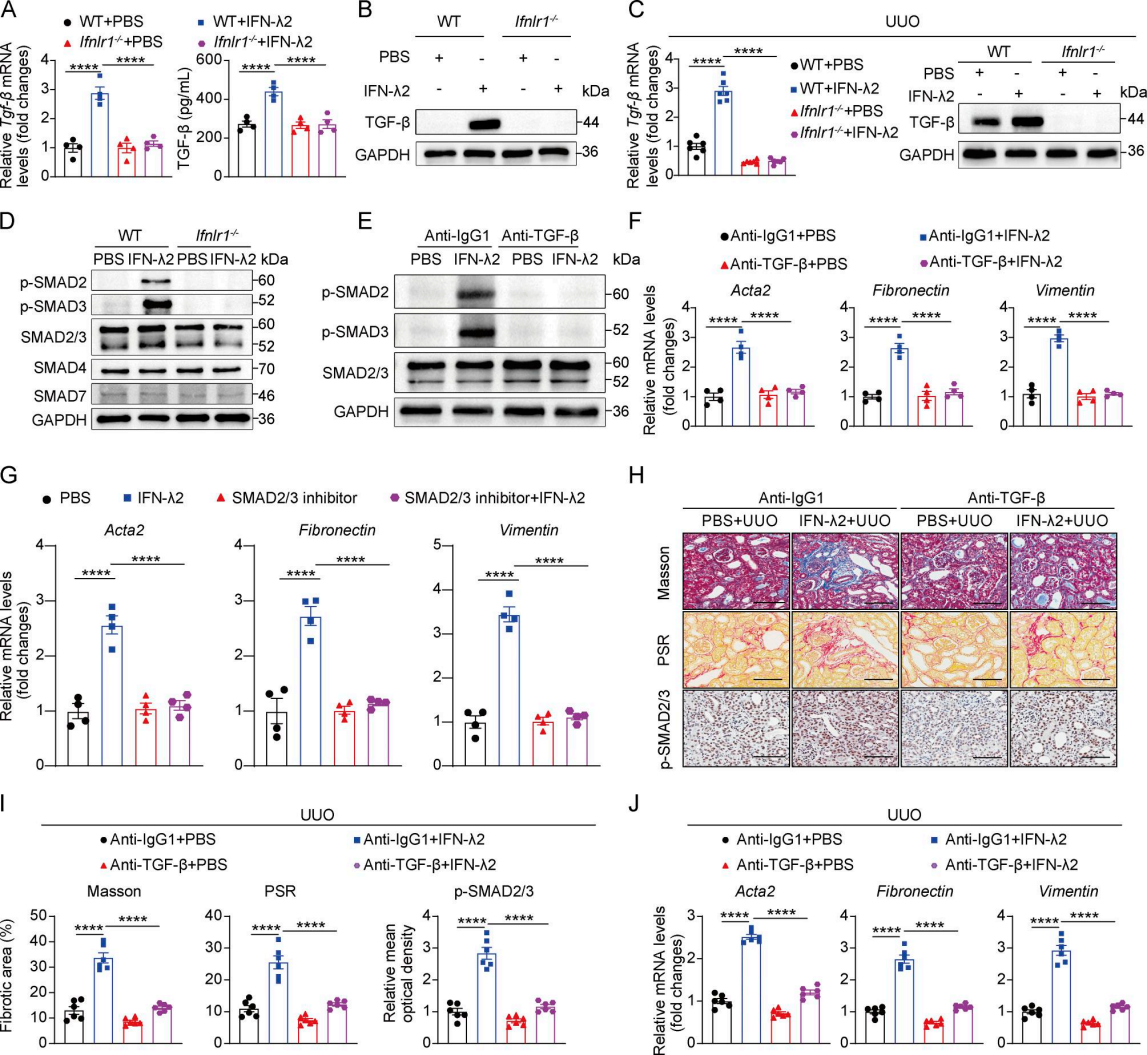
**IFN-λ promotes TGF-β–Smad2/3 signaling in renal fibroblasts that drives fibrogenesis. (A and B)** Primary renal fibroblasts from WT and *Ifnlr1*^*−/−*^ mice were treated with 100 ng/ml of IFN-λ2 or PBS for 24 h (*n* = 4). **(A)***Tgf-β* mRNA levels were assessed by RT-qPCR, while the culture supernatants were evaluated for TGF-β by ELISA. **(B)** TGF-β protein expression in cell lysates was examined by western blot. GAPDH was employed as a loading control. **(C)** WT and *Ifnlr1*^*−/−*^ mice (*n* = 6 per group) underwent UUO surgery and were subcutaneously injected with IFN-λ2 (1 μg) or PBS on days −1, 1, 3, and 5. Renal fibroblasts were isolated on day 7 after surgery to measure TGF-β mRNA and protein levels using RT-qPCR and western blot. **(D)** Primary kidney fibroblasts from WT and *Ifnlr1*^*−/−*^ mice were treated with 100 ng/ml of IFN-λ2 or PBS for 24 h. Western blot was used to assess the phosphorylated or total SMAD2/3, SMAD4, and SMAD7 levels. **(E and F)** Primary renal fibroblasts from WT mice were treated with 100 ng/ml IFN-λ2 or PBS in the presence of 2 μg/ml anti-TGF-β–neutralizing antibody (anti-TGF-β) or IgG1 isotype control (anti-IgG1) for 24 h (*n* = 4). **(E)** The phosphorylated and total SMAD2/3 levels were analyzed by western blot. **(F)***Acta2*, fibronectin*,* and vimentin mRNA levels were measured by RT-qPCR. **(G)** Primary renal fibroblasts from WT mice were treated with 100 ng/ml of IFN-λ2 or PBS in the presence or absence of 20 μM SMAD2/3 inhibitor (LY2109761) for 24 h (*n* = 4). *Acta2*, fibronectin*,* and vimentin mRNA levels were determined by RT-qPCR. **(H–J)** WT mice were subjected to UUO surgery and received subcutaneous injections of 1 μg of IFN-λ2 or PBS, combined with intraperitoneal injections of 25 μg of TGF-β–neutralizing antibody (anti–TGF-β) or IgG isotype control (anti-IgG1) on days −1, 1, 3, and 5. The kidneys were collected on day 7 after surgery (*n* = 6). **(H and I)** Representative images and quantitative analysis of fibrotic area and p-SMAD2/3 MOD using Masson’s trichrome and PSR staining and p-SMAD2/3 staining (scale bars = 50 μm). **(J)***Acta2*, fibronectin*,* and vimentin mRNA levels were determined by RT-qPCR. Data are representative of two (H) or three (A–G) independent experiments. Data in I and J are pooled from two independent experiments. Data are shown as mean ± SEM. ****P < 0.0001, by two-way ANOVA with Tukey’s multiple-comparison test. MOD, mean OD. Source data are available for this figure: SourceData F4.

**Figure S4.**
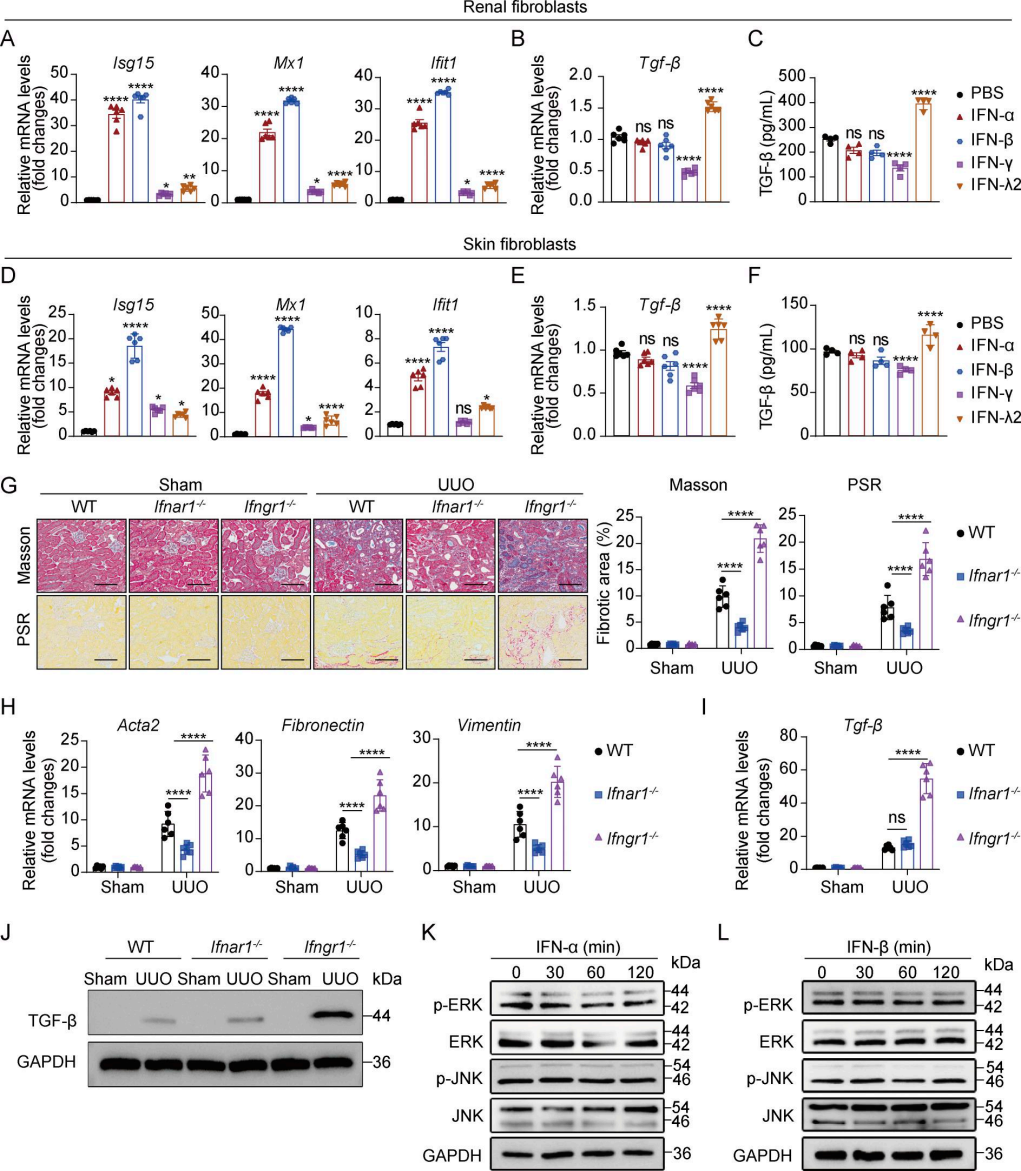
**Type I and II IFNs differ from type III IFN in their regulation of TGF-β expression and the ERK–JNK pathway in renal fibroblasts during kidney fibrosis. (A–C)** Primary renal fibroblasts from WT mice were treated for 24 h with 100 ng/ml of various IFNs subtypes (IFN-α, IFN-β, IFN-γ, or IFN-λ2) or PBS. **(A and B)***Isg15*, *Mx1*, *Ifit1*, and *Tgf-β* mRNA levels were assessed by RT-qPCR (*n* = 6), and (C) TGF-β protein in the culture supernatants was quantified by ELISA (*n* = 4). **(D–F)** Primary skin fibroblasts from WT mice were treated for 24 h with 100 ng/ml of various IFNs subtypes (IFN-α, IFN-β, IFN-γ, or IFN-λ2) or PBS. **(D and E)***Isg15*, *Mx1*, *Ifit1*, and *Tgf-β* mRNA levels were detected by RT-qPCR (*n* = 6), and (F) TGF-β protein in the culture supernatants was measured by ELISA (*n* = 4). **(G–J)** WT, *Ifnar*^*–/–*^, and *Ifngr1*^*−/−*^ mice were subjected to sham or UUO surgery, and kidneys were collected on day 7. *n *= 6 per group. **(G)** Representative images and quantitative analysis of fibrotic areas with Masson’s trichrome and PSR staining (scale bars = 50 μm). **(H)** RT-qPCR analysis of *Acta2*, fibronectin, and vimentin mRNA levels in kidneys. TGF-β mRNA and protein levels in kidneys were measured by RT-qPCR (I) and western blot (J). **(K and L)** Primary kidney fibroblasts were treated with 100 ng/ml IFN-α (K) or IFN-β (L) for the indicated times. **(K and L)** Western blot analysis of phosphorylated and total ERK and JNK protein levels. Data in A–I are pooled from two independent experiments. Data in J–L are representative of three independent experiments. Data are presented as mean ± SEM. *P < 0.05, ****P < 0.0001, by two-way ANOVA with Tukey’s multiple-comparison test (A–I). ns, no significant difference. Source data are available for this figure: SourceData FS4.

TGF-β orchestrates renal fibrogenesis by activating fibroblasts through autocrine and paracrine mechanisms to induce fibrotic factor expression (Meng et al., 2016). We hypothesized that IFN-λ may potentiate TGF-β signaling in renal fibroblasts, thereby exacerbating renal fibrosis. To test this hypothesis, we first analyzed the effects of IFN-λ on TGF-β expression in renal fibroblasts. Primary renal fibroblasts from WT mice stimulated with IFN-λ2 exhibited marked upregulation of TGF-β mRNA and protein, an effect that was abolished by IFN-λ receptor deficiency (Fig. 4, A and B). *In vivo* validation using renal fibroblasts isolated from IFN-λ–treated WT and *Ifnlr1*^*−/−*^ mice following UUO surgery demonstrated that IFN-λ robustly induced TGF-β mRNA and protein expression in WT fibroblasts, with complete abrogation in *Ifnlr1*^*−/−*^ counterparts (Fig. 4 C). Since type I (IFN-α/β), II (IFN-γ), and III IFNs activate the overlapping JAK–STAT1 pathway, we next asked whether TGF-β induction was specific to IFN-λ. Treatment of mouse primary renal fibroblasts or skin fibroblasts with IFN-α, IFN-β, or IFN-γ uniformly induced ISG expression (Fig. S4, A and D). However, neither IFN-α nor IFN-β altered TGF-β mRNA and protein expression in renal fibroblasts or skin fibroblasts, and IFN-γ significantly suppressed it (Fig. S4, B, C, E, and F). To further dissect the roles of IFN-α/β and IFN-γ *in vivo*, we subjected IFN-α/β receptor–deficient (*Ifnαr1*^*−/−*^) and IFN-γ receptor–deficient (*Ifnγr1*^*−/−*^) mice to UUO. *Ifnαr1*^*−/−*^ mice developed attenuated fibrosis, while *Ifnγr1*^*−/−*^ mice exhibited an exacerbated fibrotic phenotype (Fig. S4, G and H). Consistently, TGF-β mRNA and protein expression in renal fibroblasts was unaffected in *Ifnαr1*^*−/−*^ UUO mice but was increased in *Ifnγr1*^*−/−*^ UUO mice (Fig. S4, I and J). Collectively, these results demonstrate that TGF-β induction is a specific function of IFN-λ signaling in renal fibroblasts during renal fibrosis.

Given TGF-β's canonical activation of SMAD-dependent pathways (Derynck and Zhang, 2003; Meng et al., 2016), we interrogated the impact of IFN-λ on TGF-β–mediated SMAD signaling. Western blot analysis revealed that IFN-λ2 selectively enhanced SMAD2 and SMAD3 phosphorylation without altering SMAD2/3, SMAD4, and SMAD7 levels in renal fibroblasts (Fig. 4 D). Neutralizing TGF-β with a blocking antibody abolished IFN-λ2–derived SMAD2/3 activation and suppressed profibrotic factors (*Acta2*, fibronectin, and vimentin) (Fig. 4, E and F). Pharmacological SMAD2/3 inhibition similarly attenuated IFN-λ2–induced profibrotic gene expression (Fig. 4 G). These results demonstrated that IFN-λ induces TGF-β synthesis in renal fibroblasts and activates the TGF-β-SMAD2/3 signaling axis, thereby driving the expression of profibrotic mediators.

To establish *in vivo* relevance, WT mice subjected to UUO received IFN-λ2 with or without TGF-β–neutralizing antibody. Anti–TGF-β treatment mitigated IFN-λ–derived renal fibrosis, as shown by reduced SMAD2/3 activation, collagen deposition and fibrotic lesions (Masson’s trichrome and PSR staining) (Fig. 4, H and I), and fibrotic marker expression (Fig. 4 J). These data demonstrated that IFN-λ exacerbates renal fibrosis by amplifying TGF-β synthesis and downstream SMAD2/3 signaling in renal fibroblasts.

### IFN-λ promotes TGF-β synthesis in renal fibroblasts through the activation of ERK and JNK signaling

**Figure 5.**
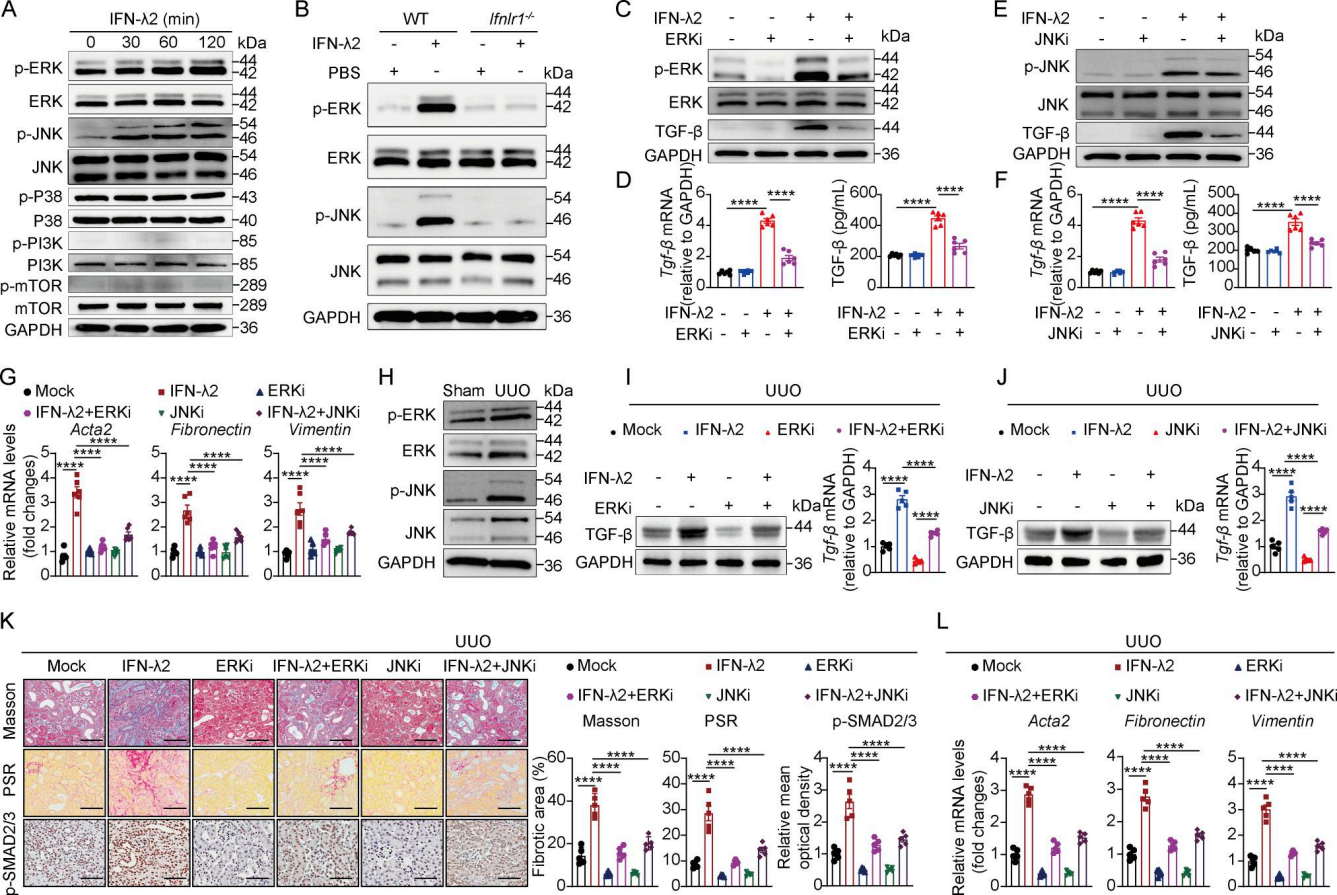
**IFN-λ induces TGF-β synthesis in renal fibroblasts through activation of the ERK and JNK signaling pathways. (A)** Primary renal fibroblasts from WT mice were treated IFN-λ2 (100 ng/ml) for 0, 30, 60, and 120 min. Phosphorylated and total ERK, JNK, p38, PI3K, and mTOR were assessed by western blot. **(B)** Primary renal fibroblasts from WT and *Ifnlr1*^*−/−*^ mice were treated with IFN-λ2 (100 ng/ml) or PBS for 120 min p-ERK, ERK, p-JNK, and JNK were analyzed by western blot. **(C and D)** Primary renal fibroblasts were pretreated with ERK inhibitor SCH772984 (ERKi, 1 μM) for 1 h, then stimulated with IFN-λ2 (100 ng/ml) for 2 h (to assess ERK activation) or 24 h (to evaluate TGF-β expression) (*n* = 6). **(C)** p-ERK and ERK were assessed by western blot; TGF-β expression were analyzed by western blot (C), RT-qPCR (D, left panel), and ELISA (D, right panel). **(E and F)** Primary renal fibroblasts were pretreated with JNK inhibitor SP600125 (JNKi, 10 μM) for 1 h, then stimulated with IFN-λ2 (100 ng/ml) for 2 h (to assess JNK activation) or 24 h (to evaluate TGF-β expression) (*n* = 6). **(E)** p-JNK and JNK were assessed by western blot; TGF-β expression were analyzed by western blot (E), RT-qPCR (F, left panel), and ELISA (F, right panel). **(G)** Primary renal fibroblasts were treated with IFN-λ2 (100 ng/ml) for 24 h in the presence or absence of ERKi or JNKi. *Acta2,* fibronectin*,* and vimentin mRNA levels were measured by RT-qPCR (*n* = 6). **(H)** WT mice underwent sham or UUO surgery for 7 days. Renal fibroblasts were isolated and analyzed for p-ERK, ERK, p-JNK, and JNK by western blot. **(I and J)** WT UUO mice were subcutaneously injected with 1 μg of IFN-λ2 on days −1, 1, 3, and 5, combined with intraperitoneal administration of ERKi (50 mg/kg) (I) or JNKi (50 mg/kg) (J). Kidneys were collected at day 7 after UUO. TGF-β protein and mRNA levels in renal fibroblasts were assessed by western blot and RT-qPCR (*n* = 5). **(K)** Representative images and quantitative analysis of fibrotic area and p-SMAD2/3 MOD in kidneys using Masson’s trichrome, PSR, and IHC (scale bars = 50 μm) (*n* = 5). **(L)** Renal *Acta2,* fibronectin*,* and vimentin mRNA levels were measured by RT-qPCR (*n* = 5). Data are representative of three (A–C, E, and H–J) independent experiments. Data in D, F–G, and K–L are pooled from two independent experiments. Data are shown as mean ± SEM. ****P < 0.0001, by two-way ANOVA with Tukey’s multiple-comparison test. MOD, mean OD. Source data are available for this figure: SourceData F5.

As TGF-β is not a classic ISG, we reasoned that its induction by IFN-λ in renal fibroblasts may occur through noncanonical signaling pathways, particularly the MAPK and phosphoinositide 3-kinase-mechanistic target of rapamycin (PI3K-mTOR) cascades (Alase et al., 2015; Brand et al., 2005; Chai et al., 2011; Syedbasha et al., 2020; Zhou et al., 2007), both known to regulate TGF-β expression (Xiao et al., 2008). We therefore sought to identify which pathway mediates IFN-λ–induced TGF-β expression. Treatment of mouse primary renal fibroblasts with IFN-λ2 effectively activated the MAPK members ERK and JNK signaling, while p38 MAPK and PI3K-mTOR signaling were unaffected (Fig. 5 A). Functional ablation of the IFN-λ receptor in renal fibroblasts abolished IFN-λ2–induced activation of ERK and JNK in renal fibroblasts. (Fig. 5 B). In contrast, neither IFN-α nor IFN-β activated ERK or JNK in renal fibroblasts, underscoring the specificity of the response to IFN-λ (Fig. S4, K and L). Importantly, small-molecule inhibitors targeting ERK (Fig. 5, C and D) or JNK (Fig. 5, E and F) abolished the IFN-λ2–induced upregulation of TGF-β mRNA and protein in renal fibroblasts, as well as the expression of profibrotic genes *in vitro* (Fig. 5 G).

We next confirmed *in vivo* that ERK and JNK were significantly activated in UUO mice (Fig. 5 H). Administration of small-molecule inhibitors to block ERK or JNK activity in UUO mice markedly suppressed the IFN-λ2–induced expression of TGF-β mRNA and protein in renal fibroblasts (Fig. 5, I and J). In UUO mice, pharmacological inhibition of ERK or JNK ameliorated IFN-λ2–induced renal fibrosis, as evidenced by reduced collagen deposition and fibrotic lesions (Masson’s trichrome and PSR staining), downregulated SMAD2/3 activation, and diminished profibrotic genes (*Acta2*, fibronectin, and vimentin) (Fig. 5, K and L). These results indicate that IFN-λ drives TGF-β production and fibrotic signaling in renal fibroblasts primarily through the ERK and JNK pathways.

### RIG-I/MAVS signaling–dependent IFN-λ secretion in renal TECs during renal fibrosis

**Figure 6.**
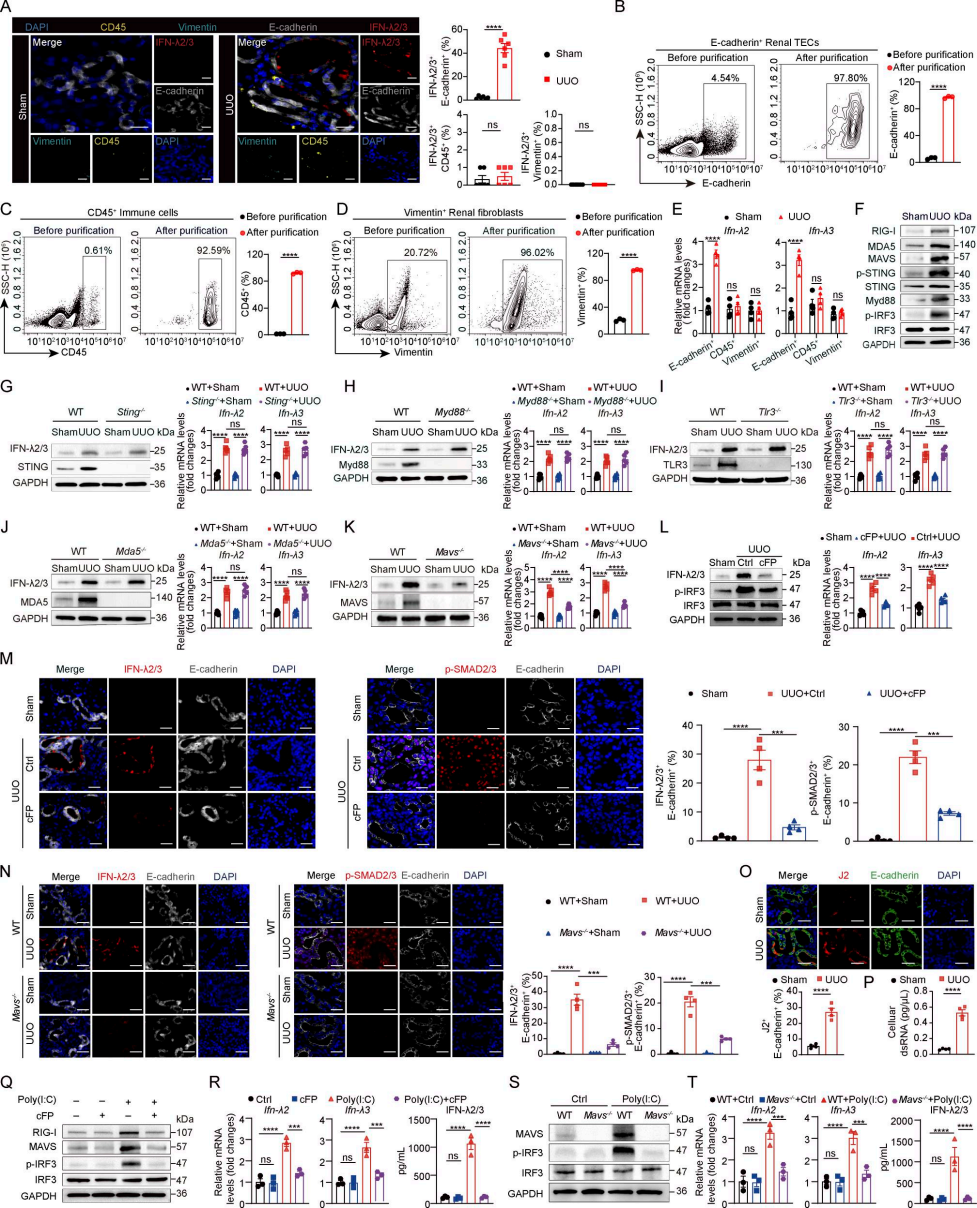
**Renal TECs generate IFN-λ upon RIG-I/MAVS signaling activation. (A)** Multiplex immunofluorescence staining for IFN-λ2/3 (red), TECs (E-cadherin, white), fibroblasts (vimentin, cyan), and immune cells (CD45, yellow) in kidney sections at day 7 from sham-operated mice (sham) and UUO mice. Scale bars = 50 μm. Quantification of IFN-λ2/3–positive cells are shown on the right panel (*n* = 6). **(B–D)** Flow cytometry plots showing the purity of isolated E-cadherin^+^ TECs (B), CD45^+^ immune cells (C), and vimentin^+^ fibroblasts (D) from mouse kidneys (*n* = 3). **(E)** RT-qPCR analysis of *Ifn-λ2* and *Ifn-λ3* mRNA levels in the indicated purified renal cell populations from sham and UUO mice at day 7 (*n* = 4). **(F)** Western blot analysis of indicated pathway proteins in renal TECs of sham and UUO mice on day 7 (*n* = 5 per group). **(G–K)** WT, *Sting*^*–/–*^ (G), *Myd88*^*−/−*^ (H), *Trl3*^*−/−*^ (I), *Mda5*^*−/−*^ (J), and *Mavs*^*–/–*^ (K) mice were subjected to sham or UUO surgery (*n* = 5 per group). Renal TECs were isolated on day 7 after surgery and analyzed for IFN-λ2/3 and pathway protein expression by western blot (left panels) and *Ifn-λ2* and *Ifn-λ3* mRNA by RT-qPCR (right panels). **(L)** Renal TECs from sham and UUO treated with PBS or 50 mg/kg of cFP were analyzed for IFN-λ2/3, p-IRF3, and IRF3 protein levels by western blot (left panel) and *Ifn-λ2* and *Ifn-λ3* mRNA by RT-qPCR (right panel) (*n* = 5 per group). **(M)** Immunofluorescence staining for IFN-λ2/3 or p-SMAD2/3 in renal TECs (E-cadherin) in kidneys of sham and UUO mice treated with PBS or 50 mg/kg of cFP on day 7 after surgery. Nuclei were counterstained with DAPI. Quantification is shown on the right. *n* = 4 per group, scale bars = 50 μm. **(N)** Representative images and quantitative analysis of IFN-λ2/3 or p-SMAD2/3 in renal TECs (E-cadherin) in kidneys of WT and *Mavs*^*–/–*^ UUO mice at day 7. *n* = 4 per group, scale bar = 50 μm. **(O)** Representative images and quantitative analysis of dsRNA (J2) in renal TECs (E-cadherin) in kidneys of sham and UUO mice at day 7. *n* = 4 per group, scale bars = 50 μm. **(P)** ELISA analysis of dsRNA levels in isolated renal TECs. **(Q and R)** Primary renal TECs were treated with or without 2.5 mM cFP for 6 h, followed by 1 μg/ml of poly(I:C) transfection for 12 h (*n* = 3). **(Q)** Western blot analysis of RIG-I, MAVS, p-IRF3, and IRF3 protein levels. **(R)** RT-qPCR and ELISA were used to evaluate IFN-λ2/3 mRNA and protein levels, respectively. **(S and T)** Renal TECs isolated from WT and *Mavs*^*–/–*^ mice were transfected with 1 μg/ml of poly(I:C) for 12 h (*n* = 3). **(S)** Western blot analysis of MAVS, p-IRF3, and IRF3 protein levels. **(T)** IFN-λ2/3 mRNA and protein levels were determined by RT-qPCR and ELISA. Data in A, E, and G–P are pooled from two independent experiments. Data in B–D are pooled from three independent experiments. Data in F and Q–T are representative of three independent experiments. Data are presented as mean ± SEM. ***P < 0.001, ****P < 0.0001, by unpaired two-tailed Student’s *t* test (A–F and O–P), two-way ANOVA with Tukey’s multiple-comparison test (G–K, N, R, and T), and one-way ANOVA with Tukey’s multiple-comparison test (L and M). ns, no significant difference. Source data are available for this figure: SourceData F6.

To identify the IFN-λ–producing cell types in renal fibrosis, multiple immunofluorescence staining was performed on renal tissues from sham or UUO-induced mice using antibodies specific for IFN-λ2/3, E-cadherin (TEC marker), vimentin (fibroblast marker), and CD45 (immune marker). At day 7 after UUO, IFN-λ2/3 expression was predominantly localized to renal TECs, with negligible detection in fibroblasts or immune cells (Fig. 6 A). To confirm this, we purified renal E-cadherin^+^ TECs, CD45^+^ immune cells, and vimentin^+^ fibroblast populations by flow cytometry (Fig. 6, B–D). RT-PCR analysis demonstrated that *Ifn-λ2* and *Ifn-λ3* mRNA expression was predominantly restricted to the E-cadherin^+^ TEC fraction from UUO mice (Fig. 6 E). Collectively, these results identify renal TECs as the principal IFN-λ source in renal fibrosis.

**Figure S5.**
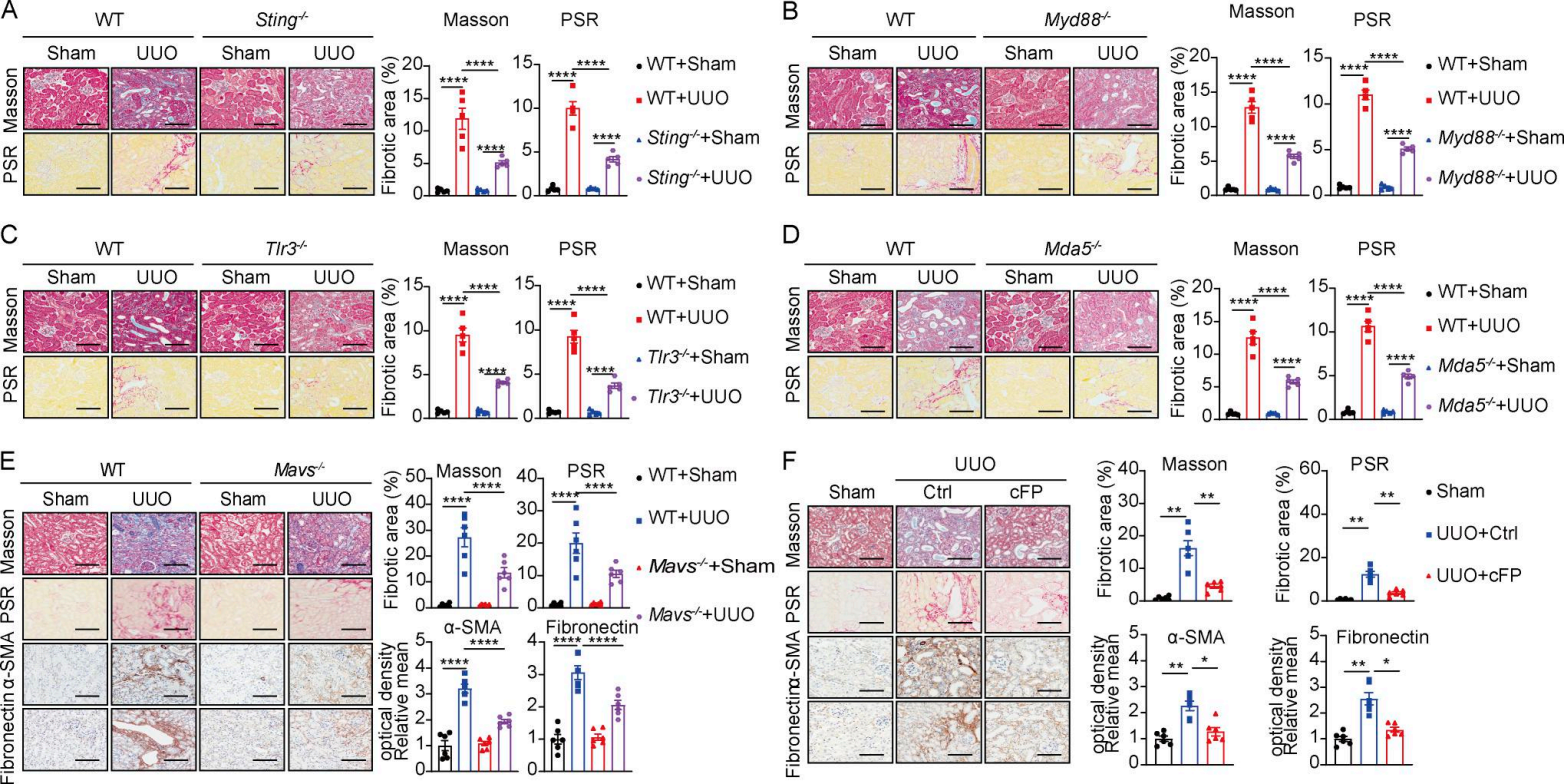
**Inhibition of STING, Myd88, TLR3, or RIG-I/MAVS signaling attenuates renal fibrosis in mice. (A–D)** Representative images and quantitation of the fibrotic areas with Masson’s trichrome and PSR staining in the kidneys from WT, *Sting*^*–/–*^ (A), *Myd88*^*−/−*^ (B), *Trl3*^*−/−*^ (C), and *Mda5*^*−/−*^ (D) mice subjected to sham or UUO mice. Scale bars = 50 μm. **(E)** Representative images and quantitative analysis of Masson’s trichrome, PSR, α-SMA, and fibronectin staining in kidneys from WT and *Mavs*^*–/–*^ UUO mice. Scale bar = 50 μm. **(F)** Representative images for Masson’s trichrome, PSR, α-SMA, and fibronectin staining in the kidneys of sham and UUO mice with or without cFP treatment. Scale bars = 50 μm. Data are pooled from two independent experiments with a total of five to six mice per group. Data are presented as mean ± SEM. *P < 0.05, **P < 0.01, ****P < 0.0001, by two-way ANOVA with Tukey’s multiple-comparison test (A–E) or one-way ANOVA with Tukey’s multiple-comparison test (F).

To interrogate the innate immune signals required for IFN-λ production in renal TECs, we initially surveyed key pattern recognition receptor (PRR) pathways in fibrotic kidneys, including TLRs, RIG-I–like receptors, and cytosolic DNA sensors. At day 7 after UUO, renal TECs showed concurrent increases in the levels of RIG-I, melanoma differentiation-associated protein 5 (MDA5), P-STING, MAVS, IRF3, and Myd88 (Fig. 6 F). Subsequent functional assessment using respective knockout mice (*Sting*^*–/–*^, *Tlr3*^*−/−*^, *Mda5*^*−/−*^, *Myd88*^*−/−*^, or *Mavs*^*–/–*^) and a RIG-I inhibitor (cFP) revealed that, although blocking these PRR pathways attenuated renal fibrosis in UUO mice (evidenced by Masson’s trichrome/PSR staining or profibrotic marker expression) (Fig. S5, A–F), only genetic deletion of MAVS or pharmacological inhibition of RIG-I profoundly reduced IFN-λ2/3 protein and mRNA expression in renal TECs (Fig. 6, G–L). Consistently, genetic deletion of MAVS or pharmacological inhibition of RIG-I (cFP) reduced the proportion of IFN-λ2/3-positive renal TECs and diminished SMAD2/3 phosphorylation–positive renal TECs (Fig. 6, M and N). These findings indicate that the RIG-I–MAVS signaling axis is specifically required for IFN-λ production in renal TECs during renal fibrosis.

Given that RIG-I recognizes double-stranded RNA (dsRNA), we hypothesized that endogenous dsRNA might serve as its ligand in this context. Immunostaining with the dsRNA-specific J2 antibody and ELISA revealed a significant accumulation of intracellular dsRNA specifically in renal TECs of UUO mice compared with sham controls (Fig. 6, O and P), suggesting dsRNA as a key endogenous activator of RIG-I. To establish a causal link, we stimulated primary renal TECs with poly(I:C), a dsRNA analog. Poly(I:C) treatment in renal TECs induced coordinated upregulation of RIG-I and MAVS and phosphorylated IRF3, whereas pharmacological blockade of RIG-I activation abolished poly(I:C)-induced IRF3 activation (Fig. 6 Q). Critically, poly(I:C) significantly upregulated both gene transcription and protein expression of IFN-λ2/3 in renal TECs in a RIG-I–dependent manner (Fig. 6 R). To validate this observation, we isolated primary renal TECs from WT and *Mavs*^*–/–*^ mice and treated them with poly(I:C) for 12 h. MAVS signaling deficiency in renal TECs largely prevented poly(I:C)-induced IRF3 activation, resulting in reduced IFN-λ2/3 mRNA and protein expression (Fig. 6, S and T). Together, these data demonstrate that dsRNA activates the RIG-I–MAVS axis in renal TECs to drive IFN-λ expression during renal fibrosis.

### Renal TEC-derived IFN-λ promotes renal fibroblast migration and activation

**Figure 7.**
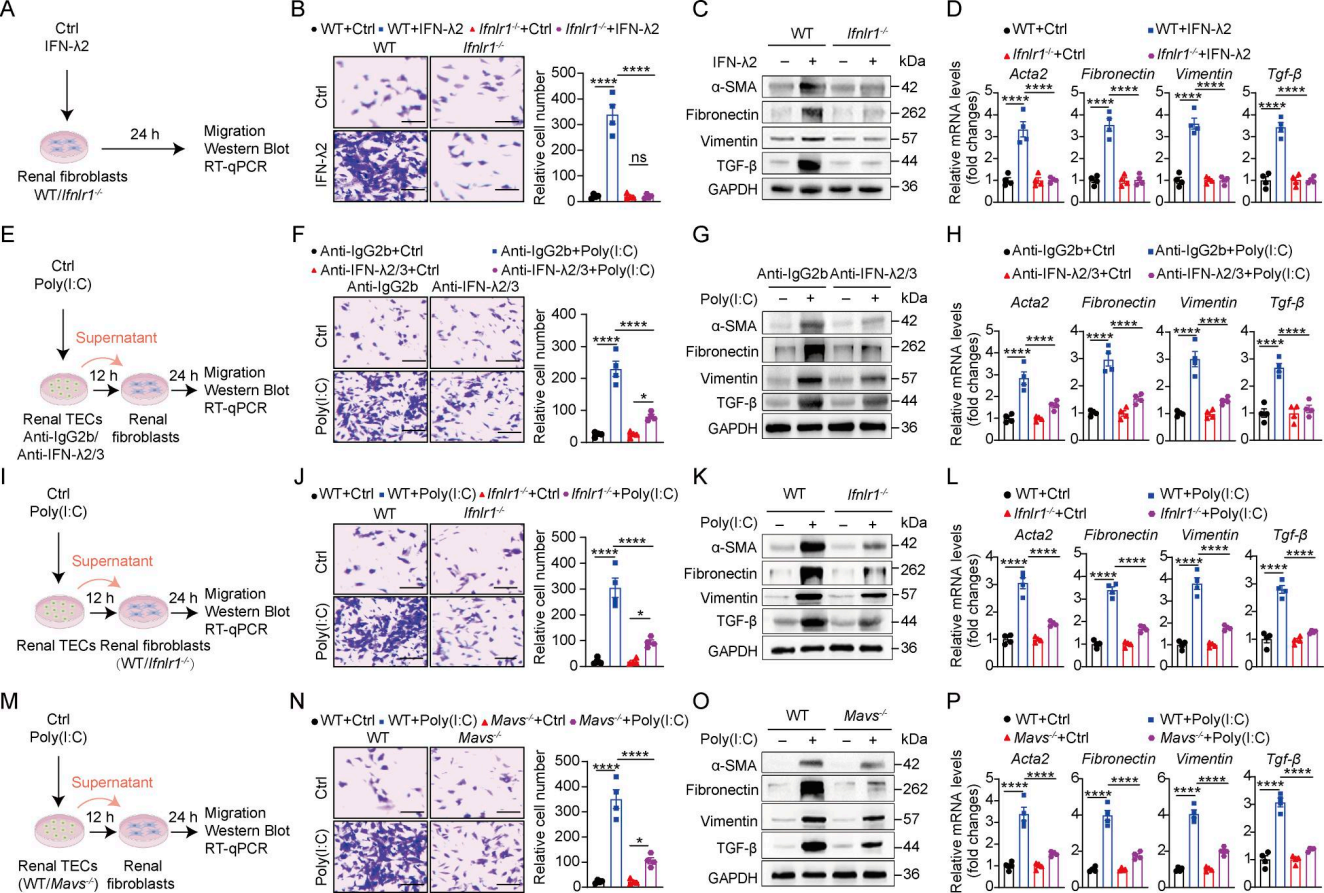
**Renal TEC-derived IFN-λ drives renal fibrosis by promoting renal fibroblast activation and migration. (A–D)** Primary renal fibroblasts from WT and *Ifnlr1*^*−/−*^ mice were treated with 100 ng/ml of IFN-λ2 or PBS for 24 h (A). *n* = 4 per group. **(B)** Representative images and quantification of the migrated renal fibroblasts. **(C)** The indicated protein levels were measured by western blot. **(D)***Acta2*, fibronectin*,* vimentin, and *Tgf-β* mRNA levels in fibroblasts were detected by RT-qPCR. **(E–H)** Renal TECs were transfected with 1 μg/ml of poly(I:C) in the presence of 10 μg/ml of anti-IFN-λ2/3–neutralizing antibody (anti–IFN-λ2/3) or IgG2b isotype control (anti-IgG2b) for 12 h. The supernatants were collected and applied to primary renal fibroblasts for 24 h (E). *n *= 4 per group. **(F)** Representative images and quantification of the migrated renal fibroblasts. **(G)** The indicated protein levels were detected by western blot. **(H)***Acta2*, fibronectin, vimentin, and *Tgf-β* mRNA levels in fibroblasts were measured by RT-qPCR. **(I–L)** Renal TECs were transfected with or without 1 μg/ml poly(I:C) for 12 h. The supernatants were then collected and added to the culture medium of primary renal fibroblasts from WT and *Ifnlr1*^*−/−*^ mice for 24 h (I). *n* = 4 per group. **(J)** Representative images and quantification of the migrated renal fibroblasts. **(K)** Western blot was used to quantify the indicated proteins in renal fibroblasts. **(L)***Acta2*, fibronectin, vimentin, and *Tgf-β* mRNA levels in renal fibroblasts were assessed by RT-qPCR. **(M–P)** Renal TECs from WT and *Mavs*^*–/–*^ mice were transfected with 1 μg/ml of poly(I:C) for 12 h. Supernatants were collected and added to primary renal fibroblasts for 24 h (M). *n* = 4 per group. **(N)** Representative images and quantification of the migrated renal fibroblasts. **(O)** Western blot was utilized to quantify the indicated proteins in renal fibroblasts. **(P)***Acta2*, fibronectin, vimentin, and *Tgf-β* mRNA levels were measured by RT-qPCR. Scale bar = 50 μm (B, F, J, and N). Data in A–P are representative of two independent experiments. Data are presented as mean ± SEM. *P < 0.05, ****P < 0.0001, by two-way ANOVA with Tukey’s multiple-comparison test. ns, no significant difference. Source data are available for this figure: SourceData F7.

To investigate whether IFN-λ directly affects renal fibroblast activity, we treated WT and *Ifnlr1*^*−/−*^ renal fibroblasts with IFN-λ2 (Fig. 7 A). Migration assays showed IFN-λ2 robustly induced fibroblast migration (Fig. 7 B) and upregulated fibrotic markers (α-SMA [encoded by Acta2], fibronectin, vimentin, and TGF-β) at both protein and mRNA levels (Fig. 7, C and D), all of which required functional IFN-λ receptor signaling (Fig. 7, B–D).

To further investigate the role of renal TEC-derived IFN-λ for renal fibroblast activity, we treated primary renal fibroblasts with supernatants from poly(I:C)-treated renal TECs in the presence of IFN-λ2/3–neutralizing antibodies for 24 h (Fig. 7 E). Migration assays demonstrated that supernatants from poly(I:C)-induced renal TEC enhanced renal fibroblast migration, which was significantly inhibited by IFN-λ2/3 blockade (Fig. 7 F). These supernatants also increased expression of fibrotic factor α-SMA, fibronectin, vimentin, and TGF-β at both transcription and protein levels, and this activity was largely dependent on the presence of IFN-λ2 (Fig. 7, G and H).

To validate the view that the regulatory effect of IFN-λ on renal fibroblasts depends on the IFN-λ receptor, we stimulated WT and *Ifnlr1*^*−/−*^ fibroblasts with supernatants from poly(I:C)-treated renal TECs for 24 h (Fig. 7 I). Migration assays demonstrated that poly(I:C)-induced TEC supernatants significantly enhanced fibroblast migration of WT cells but failed to elicit this effect in *Ifnlr1*^*−/−*^ fibroblasts (Fig. 7 J). Notably, these supernatants upregulated both protein and RNA expression of α-SMA, fibronectin, vimentin, and TGF-β in fibroblasts from WT mice, whereas this profibrotic response was abrogated in IFN-λ receptor–deficient fibroblasts (Fig. 7, K and L).

To determine the role of MAVS in poly(I:C)-mediated fibroblast migration and activation, we stimulated renal fibroblasts with supernatants from poly(I:C)-treated renal TECs derived from WT or *Mavs*^*–/–*^ mice for 24 h (Fig. 7 M). Migration assays demonstrated that supernatants from poly(I:C)-treated WT renal TECs significantly enhanced renal fibroblast migration, whereas MAVS deficiency in renal TECs largely abolished this migratory effect (Fig. 7 N). Crucially, western blot and RT-qPCR analysis demonstrated that MAVS signaling in TECs was required for poly(I:C)-induced upregulation of fibrotic markers α-SMA, fibronectin, vimentin, and TGF-β in fibroblasts (Fig. 7, O and P). Taken together, these findings demonstrated that renal TEC-derived IFN-λ is essential for mediating profibrotic factor expression and conferring migratory capacity in renal fibroblasts.

### Targeting IFN-λ therapy improves renal fibrosis in mice

**Figure 8.**
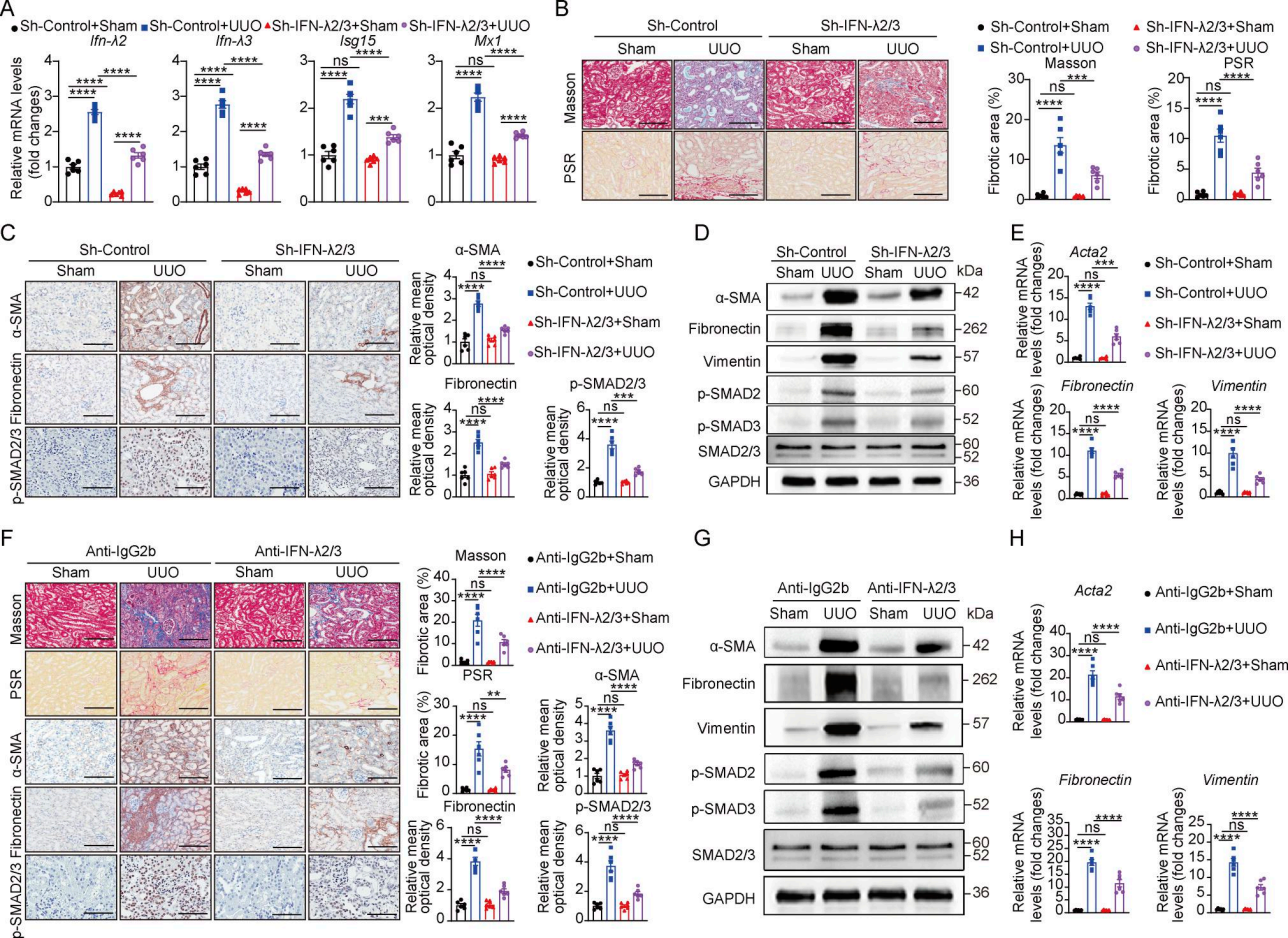
**Neutralizing IFN-λ improves renal fibrosis in mice. (A–E)** WT mice received intrarenal injections of an adenovirus vector expressing shRNA-targeting mouse IFN-λ2/3 (Sh-IFN-λ2/3) and control adenovirus-CMV-GFP (Sh-control) prior to sham or UUO surgery. Kidneys were harvested on day 7 after surgery. *n* = 6 per group. **(A)** RT-qPCR analysis for renal *Ifn-λ2*, *Ifn-λ3*, *Isg15*, and *Mx1* mRNA levels among groups. **(B)** Representative images and quantitative analysis of fibrotic areas with Masson’s trichrome and PSR staining (scale bars = 50 μm). **(C)** Representative images and quantitative analysis of α-SMA, fibronectin, and p-SMAD2/3 MOD in mouse kidneys (scale bars = 50 μm). **(D)** Western blot examination of α-SMA, fibronectin, vimentin, phosphorylated and total SMAD2/3 proteins in mouse kidneys. **(E)** RT-qPCR analysis of *Acta2*, fibronectin, and vimentin mRNA levels in mouse kidneys. **(F–H)** WT UUO mice received intraperitoneal injections with anti-IFN–λ2/3 neutralizing antibody (anti–IFN-λ2/3) or IgG2b isotype control (anti-IgG2b) on days −1, 1, 3, and 5. The kidneys were collected at day 7 after UUO surgery (*n* = 6 per group). **(F)** Representative images and quantification analysis of fibrotic area, α-SMA, fibronectin, and p-SMAD2/3 using Masson’s trichrome, PSR, and IHC staining (scale bars = 50 μm). **(G)** Western blot analysis of α-SMA, fibronectin, vimentin, phosphorylated, and total SMAD2/3 proteins in mouse kidneys. **(H)** RT-qPCR analysis of *Acta2*, fibronectin, and vimentin mRNA levels in mouse kidneys. Data in A–H are representative of three independent experiments. Data are presented as mean ± SEM. **P < 0.01, ***P < 0.001, ****P < 0.0001, by two-way ANOVA with Tukey’s multiple-comparison test. ns, no significant difference. MOD, mean OD. Source data are available for this figure: SourceData F8.

To evaluate a new therapeutic strategy for renal fibrosis, we employed adenovirus-mediated silencing of the *Ifn-λ2/3* gene in mice. Delivery of an adenovirus vector expressing shRNA against *Ifn-λ2/3* (Sh-IFN-λ2/3), but not a control shRNA (Adenovirus-CMV-GFP, Sh-control), effectively suppressed the expression of both *Ifn-λ2* and *Ifn-λ3* mRNA and downstream ISG (*Isg15* and *Mx1*) in the kidney of UUO mice (Fig. 8 A). Following intrarenal delivery of Sh-IFN-λ2/3 and Sh-control to UUO mice, we found a significant reduction of fibrotic areas (Masson’s trichrome and PSR staining), fibrosis-related factors (α-SMA and fibronectin), and SMAD2/3 phosphorylation in the kidneys of Sh-IFN-λ2/3–treated UUO mice compared with controls (Fig. 8, B and C). Consistently, western blot analysis showed reduced SMAD2/3 activation and lower protein levels of the profibrotic markers (α-SMA, fibronectin, and vimentin) in the Sh-IFN-λ2/3–treated fibrotic kidneys (Fig. 8 D), while RT-qPCR confirmed downregulation of profibrotic markers at the transcriptional level in the Sh-IFN-λ2/3–treated fibrotic kidneys (Fig. 8 E).

To further validate the therapeutic benefits of IFN-λ depletion in renal fibrosis, we evaluated the effect of IFN-λ2/3–neutralizing antibodies. Intraperitoneal administration of anti-IFN-λ2/3–neutralizing antibodies significantly attenuated renal damage and collagen deposition (evidenced by Masson’s trichrome and PSR staining) (Fig. 8 F), reduced fibrotic progression (confirmed by αSMA and fibronectin staining), and suppressed SMAD2/3 activation in UUO mice (Fig. 8 F). Western blot analysis further verified the effective suppression of SMAD2/3 activation and reduced fibrotic markers (α-SMA, fibronectin, and vimentin) at protein levels following anti–IFN-λ2/3 antibody treatment, while RT-qPCR verified parallel downregulation of fibrotic markers at the mRNA level (Fig. 8, G and H). These results collectively indicated that blocking IFN-λ activity represents a promising therapeutic strategy for ameliorating renal fibrosis.

## Discussion

This study identified IFN-λ as an important regulator of renal fibrosis. We found that renal TECs can secrete IFN-λ in response to stress-induced RIG-I–MAVS signaling and that the resulting IFN-λ then activates renal fibroblasts to secrete TGF-β by activating ERK and JNK signaling pathways, which, in turn, promotes fibroblast migration and excessive profibrotic factor synthesis. IFN-λ is well-known for its protective role in antiviral defense at barrier surfaces (Mahlakoiv et al., 2015; Sommereyns et al., 2008; Ye et al., 2019a; Ye et al., 2019b). Our study extends the known spectrum of biological activities of IFN-λ and identifies IFN-λ as a tissue-specific profibrotic amplifier that orchestrates the renal TEC-fibroblast crosstalk during renal fibrosis. Thus, pharmaceutic targeting of IFN-λ emerges as a novel therapeutic concept for CKD.

We found that selective genetic ablation of the IFN-λ receptor in renal fibroblasts strongly attenuated renal fibrosis in mice, highlighting the critical role of IFN-λ activity on fibroblasts during CKD. The observation that IFN-λ is acting on renal fibroblasts was a big surprise as previous studies had suggested that the IFNLR1 is only expressed by a few cell types, including epithelial cells and neutrophils, but not by fibroblasts (Ank et al., 2008; Lasfar et al., 2006; Sommereyns et al., 2008; Ye et al., 2019b). Analysis of public scRNA-seq data revealed that the IFN-λ receptor is readily detectable in fibroblasts from both patients with CKD and UUO mice. More detailed work showed that IFN-λ readily acts on primary fibroblasts derived from kidney and skin but fails to act on fibroblasts from the peritoneum or on embryo fibroblasts, revealing that the responsiveness of fibroblasts to IFN-λ is dependent on the tissue source and cellular context. Our findings are in line with recent data showing that IFN-λ can activate human dermal fibroblasts but not embryo fibroblasts (Alase et al., 2015; Ank et al., 2008; Lasfar et al., 2006).

Our work revealed that renal TECs, rather than immune cells or fibroblasts, are the major source of IFN-λ in the kidney of UUO mice, supporting previous conclusions that renal parenchymal cells are involved in the development of renal fibrosis (Huang et al., 2023; Liu, 2011). Multiple innate immune pathways, including cyclic GMP-AMP synthase–STING, TLR-Myd88, and RIG/MDA5-MAVS, are known to regulate IFN production and participate in renal fibrosis (Anders, 2007; Andrade-Silva et al., 2025; Qi and Yang, 2018; Zhou et al., 2020). Accordingly, we confirmed that the key components of these pathways are functionally active in renal TECs and that their genetic loss attenuates renal fibrosis in UUO mice. Strikingly, however, disruption of the RIG-I–MAVS axis, either through pharmacological inhibition of RIG-I or genetic ablation of MAVS, strongly reduced IFN-λ expression in renal TECs. Therefore, our data uncover a novel mechanism by which RIG-I–MAVS signaling in renal TECs mediates IFN-λ–dependent profibrotic processes. RIG-I was originally identified as a cytoplasmic receptor that recognizes viral dsRNA and activates the MAVS pathway, thereby triggering IFN production (Rehwinkel and Gack, 2020; Thoresen et al., 2021). Studies by others revealed distinct functions of RIG-I beyond its antiviral role, highlighting the importance of RIG-I in noninfectious diseases, including renal fibrosis (Kato and Fujita, 2015; Lei et al., 2022; Zhou et al., 2020). RIG-I protein is expressed in renal biopsy specimens from patients with active lupus nephritis and renal TECs can express RIG-I (Anders, 2007; Ma et al., 2023; Suzuki et al., 2007). Furthermore, the lack of RIG-I signaling in renal TECs can attenuate renal fibrosis in mice by suppressing NF-κB–mediated inflammation (Zhou et al., 2020). Renal TECs are rich in mitochondria and, therefore, are highly sensitive to various insults such as hypoxia, oxidative stress, and toxins (Qi and Yang, 2018). For most mammalian cells, cytoplasmic dsRNA represents an important threat (Chen and Hur, 2022; López-Polo et al., 2024), and homeostasis and dynamic turnover of mitochondrial dsRNA are crucial to cell fate (López-Polo et al., 2024; Zhu et al., 2023). In support of this, we found that renal TECs in UUO mice accumulate substantial amounts of cytoplasmic dsRNA, which likely serves as the key endogenous ligand activating RIG-I–IFN-λ axis.

An exciting finding of the present study was that IFN-λ contributes to renal fibrosis by enhancing the production of TGF-β, which is a key renal profibrotic factor (Meng et al., 2016). Notably, this ability to induce TGF-β secretion from renal fibroblast is unique to IFN-λ and is not shared by type I (IFN-α/β) or type II (IFN-γ) IFNs. Although our data confirm that IFN-α/β signaling promotes renal fibrosis while IFN-γ suppresses it, neither IFN-α nor IFN-β affected renal fibroblast–derived TGF-β, and IFN-γ actually inhibited its secretion. This indicates that type I and II IFNs operate through distinct, TGF-β–independent mechanisms. IFN-λ signals through both canonical (JAK–STAT1) and noncanonical pathways (Lazear et al., 2015; Ye et al., 2019b). We demonstrate that while the JAK–STAT1 axis is essential for IFN-λ–induced ISG expression, the IFN-λ primarily activates the ERK and JNK pathways to drive TGF-β secretion from renal fibroblasts. This aligns with prior reports establishing ERK and JNK pathways are crucial for TGF-β expression and their activation in CKD and is consistent with IFN-λ's ability to activate JNK and ERK in other cell types, such as intestinal epithelial cells and human dermal fibroblasts (Alase et al., 2015; Qi and Yang, 2018; Xiao et al., 2008). In contrast, neither type I nor type II IFNs activated ERK/JNK signaling in renal fibroblasts, underscoring the pathway specificity underlying IFN-λ's profibrotic action.

Canonical TGF-β profibrotic signaling involves recruitment and activation of TGF-β receptors 1 (TGFβR1) and TGFβR2 that activate SMAD2 and SMAD3 (Derynck and Zhang, 2003), which then form complexes with SMAD4 and translocate to the nucleus to regulate the expression of profibrotic molecules. SMAD7 usually acts as a negative regulator of SMAD2/3 to suppress fibrosis (Derynck and Zhang, 2003; Meng et al., 2016). Our data indicate that IFN-λ induces TGF-β synthesis and enhances TGF-β–mediated SMAD2/3 signaling to amplify profibrotic factor production in renal fibroblasts without affecting SMAD4 or SMAD7 expression. Conversely, we found that blocking TGF-β production with a neutralizing antibody or SMAD2/3 signaling with an inhibitor limited IFN-λ–triggered renal fibrosis. Critically, blocking ERK/JNK signaling similarly alleviated IFN-λ–mediated fibrosis by impairing TGF-β–dependent SMAD2/3 activation. This previously unknown IFN-λ–ERK/JNK–TGF-β axis establishes a self-reinforcing renal fibroblast activation loop and represents a novel compartment-specific crosstalk mechanism that drives renal fibrosis progression.

However, a limitation of our study is that we primarily focused on the UUO model, a classic and robust system, to dissect the novel “RIG-I/MAVS–IFN-λ–ERK/JNK–TGF-β axis” in driving renal fibrosis. While this model effectively recapitulates the core fibrotic progression following obstructive injury, fibrotic microenvironments may vary across etiologies (e.g., metabolic, toxic, or immune-mediated) (Abbad et al., 2025; Huang et al., 2023). Thus, the generalizability of the IFN-λ axis in other CKD models, such as folic acid nephropathy or diabetic nephropathy, awaits future validation.

Taken together, our current study identified IFN-λ not only as a biomarker of progressive renal fibrosis but also as a central mediator of CKD pathogenesis. Since renal disease could be attenuated by silencing IFN-λ in kidneys of UUO mice, our work provides first experimental evidence that targeting IFN-λ signaling is a promising new therapeutic concept for renal fibrosis.

## Materials and methods

### Mice

All mice in this study were on the C57BL/6 genetic background. WT C57BL/6 mice (designated WT) were purchased from the Guangdong Medical Laboratory Animal Center (Guangdong, China). *Mavs*^*–/–*^ mice (#T011413) were kindly provided by Prof. Xialin Liu and Dr. Wei Yi (Sun Yat-Sen University, Guangdong, China). *Ifn-λ2/3*^*–/–*^ mice (#S-KO-09527) were purchased from Cyagen Biosciences. *Sting*^*–/–*^ (#T012747), *Ifnar1*^*−/−*^ (#T005534), *Ifngr1*^*−/−*^ (#T012667), *Ifnlr1*^*−/−*^ (#T013985), *Tlr3*^*−/−*^ (#T006734), *Myd88*^*−/−*^ (#T003613), and *Mda5*^*−/−*^ (#T013946) mice were purchased from GemPharmatech Co., Ltd.

To generate fibroblast-specific *Ifnlr1* knockout mice, Col1a2-Cre/ERT mice (#T068041) were purchased from GemPharmatech Co., Ltd and *Ifnlr1*^*f/f*^ mice (#S-CKO-08469) were purchased from Cyagen Biosciences. Fibroblast-specific *Ifnlr1* knockout mice (designated *Ifnlr1*^*f/f*^*; Col1a2-*Cre) were generated by crossbreeding Col1a2-Cre/ERT mice with *Ifnlr1*^*f/f*^ mice. To induce the Cre-recombinase, mice were treated with intraperitoneal injections of tamoxifen (T5648; Sigma-Aldrich) (0.1 ml of 20 mg/ml in corn oil [c8267; Sigma-Aldrich]) for 5 consecutive days. Littermate *Ifnlr1*^*f/f*^ mice served as controls.

All experiments were conducted using 8–10-wk-old male mice on a C57BL/6 background weighing 23–27 g. Mice were bred and maintained at a local animal facility under specific pathogen-free conditions (12-h light/dark cycle, 24°C, and 40–60% humidity). All mouse experiments were authorized by the Institutional Animal Care and Use Committee of Shenzhen University Medical School (approval no. IACUC-202300025). The mice were anesthetized with isoflurane inhalation prior to surgery. All animals were euthanized by isoflurane inhalation and cervical dislocation at the end of experiments.

### UUO mouse model and treatment

The UUO-induced mouse renal fibrosis model was employed as previously described (Zhou et al., 2021). Briefly, a left-sided flank incision was performed in anesthetized WT, *Tlr3*^*−/−*^, *Myd88*^*−/−*^, *Mda5*^*−/−*^, *Sting*^*–/–*^, *Ifnl2/3*^*−/−*^, *Ifnαr1*^*−/−*^, *Ifngr1*^*−/−*^, *Mavs*^*–/–*^, *Ifnlr1*^*−/−*^, *Ifnlr1*^*f/f*^, or *Ifnlr1*^*f/f*^*; Col1a2-*Cre mice, and the left ureter of the animals was ligated with two 4-0 silk suture ties. Sham operation with the abdomen opened only was used as control. The abdomen was then repaired with running sutures, while the skin was closed with interrupted sutures. Following surgery, mice were kept in a temperature-controlled facility with a 12-h light/dark cycle and provided standard chow and water *ad libitum*. Mice were sacrificed at 0, 7, or 14 days after surgery, and the kidneys were removed for subsequent experiments.

For IFN-λ treatment, WT mice that underwent UUO surgery received subcutaneous injections of 1 μg of murine IFN-λ2 (250-33; PeproTech) on days −1, 1, 3, 5, 7, 9, 11, and 13 in a 100 μl volume. The kidneys were collected at days 0, 7, and 14 for quantitative assessment of renal fibrosis. In other experiments, WT and *Ifnlr1*^*−/−*^ mice that underwent UUO surgery were subcutaneously injected either with 1 μg of IFN-λ2 or PBS on days −1, 1, 3, and 5 before primary kidney fibroblasts were isolated for subsequent experiments at day 7.

For anti–TGF-β antibody treatment, WT mice were subjected to UUO surgery. The treatment protocol included subcutaneous injections of 100 μl containing 1 μg IFN-λ2 or PBS on days −1, 1, 3, and 5. Concurrently at each time point, mice were administered intraperitoneally with 25 μg of anti–TGF-β antibody (16-9243-85; Thermo Fisher Scientific) or anti-IgG1 isotype control antibody (02-6100; Thermo Fisher Scientific) in a 100 μl volume. Kidney tissues were collected for subsequent analysis at day 7.

For RIG-inhibitor treatment, WT mice were subjected to UUO or sham operations and were given cFP Cyclo (Phe-Pro, cFP) (HY-P1934; MedChemExpress) at 50 mg/kg in 100 μl PBS through the tail vein on days −1, 1, 2, 3, 4, 5, and 6. Mice were sacrificed on the seventh day following UUO surgery to obtain kidneys for subsequent experiments.

For pharmacological inhibition of ERK or JNK, WT mice undergoing UUO or sham surgery were treated with either ERK inhibitor SCH772984 (HY-50846; MedChemExpress) at 50 mg/kg in 100 μl PBS intraperitoneally or JNK inhibitor SP600125 (HY-12041; MedChemExpress) at 30 mg/kg in 100 μl PBS by gavage on days −1, 1, 3, and 5. Mice were sacrificed on day 7 after UUO, and kidney tissues were collected for subsequent analyses.

For anti–IFN-λ antibody treatment, WT mice were subjected to UUO or sham operations along with an intraperitoneal injection of 20 μg anti–IFN-λ2/3 antibody (MAB17892; R&D System) or anti-IgG2b isotype control antibody (MAB0061; R&D System) in a 100 μl volume on days −1, 1, 3, and 5. Mice were sacrificed on the seventh day after UUO surgery to collect kidneys for additional examinations.

### Adenovirus encoding IFN-λ2/3 shRNA treatment

The adenovirus vector expressing shRNA-targeting mouse *Ifn-λ2/3* (Sh-IFN-λ2/3) and control adenovirus-CMV-GFP (Sh-control) were purchased from Vigene Biosciences. To knock down IFN-λ expression in UUO mice, the adenovirus was delivered into the kidneys of experimental mice by intrarenal injection. Briefly, WT mice were anesthetized and fixed, exposing the left kidney. A 31-G needle was inserted from the lower pole of the kidney along the long axis and was gently pushed on the upper pole at the depth of around 0.5 cm while avoiding the renal veins, arteries, and ureters. 50 μl of Sh-IFN-λ2/3 or Sh-control at the concentration of 3 × 10^11^ pfu/ml was injected. We then waited 2–3 s before removing the needle. The kidney became white following the injection, indicating that the procedure was effective. Mice experienced UUO surgery 1 day after administering Sh-IFN-λ2/3 or Sh-control and were then sacrificed on the seventh day after UUO surgery to collect kidneys for further study.

### BM chimeras

To construct IFN-λ receptor-chimeric mice, WT and *Ifnlr1*^*−/−*^ recipient mice were lethally irradiated (2 × 5.5 Gy, 4 h interval) and reconstituted with 10^7^ donor BM cells from WT or *Ifnlr1*^*−/−*^ donor mice, respectively. Mice were used to establish the UUO model 7 wk after transplantation. After 1 wk, the animals were euthanized, and their kidneys were taken for light microscopy, IHC, and RT-qPCR analysis.

### Human kidney samples

Human kidney tissue samples were collected from six patients who underwent tumor nephrectomies or renal cystectomies without other kidney diseases as a control group. 12 patients who underwent renal biopsy for CKD and were diagnosed with IgA nephropathy or diabetic nephropathy in the Department of Nephrology of the First Affiliated Hospital of Shenzhen University. Experienced pathologists evaluated the samples for evidence of renal fibrosis. The study involving human samples was authorized by the Ethics Committee for the Clinical Investigation of Shenzhen University (PN-202500057), and it followed all applicable ethical standards and the Declaration of Helsinki. Before participating in the study, all patients provided informed consent for the collection and use of their kidney tissues for research purposes. To investigate the expression levels of IFN-λ2/3 in control subjects and patients with CKD from large cohorts, we utilized the Nephroseq version 5.0 database (http://v5.nephroseq.org) to examine *IFN-λ2* and *IFN-λ3* gene expression.

### Preparation of primary renal TECs

Fresh mouse kidneys were minced into fragments and digested in 10 ml DMEM F12 (DMEM/F12) (11320033; Thermo Fisher Scientific) containing 0.75 mg/ml of collagenase IV (LS004188; Worthington) and 0.75 mg/ml of trypsin inhibitor (T8031; Solarbio) for 45 min at 37°C. Afterward, collagenase IV activity was stopped by adding 10% FBS (A5256501; Thermo Fisher Scientific). Single-cell suspensions from kidney digestions were sequentially sieved through 70 µm (352350; Falcon) and 40-µm cell strainers (352340; Falcon) before centrifugation at 2,000 rpm for 5 min. The cell suspension was then layered onto 50% Percoll (P4937; Sigma-Aldrich) and centrifuged at 14,000 rpm for 1 h at 4°C. Following centrifugation, renal TECs were collected from the uppermost and second fractions. The harvested renal TEC fraction was pelleted by centrifugation, washed with DMEM/F12 medium (11320033; Thermo Fisher Scientific), and finally cultured in DMEM/F12 medium supplemented with 10% FBS (A5256501; Thermo Fisher Scientific) and 1% penicillin-streptomycin (PS, 15140122; Thermo Fisher Scientific) at 37°C in a CO_2_ incubator.

### Preparation of primary fibroblasts

To prepare primary kidney fibroblasts, fresh mouse kidneys were washed with cold PBS, and the outer cortex was separated from the medulla and cut into small pieces. This was followed by three rounds of washing. The diced fragments were digested in a solution of 1 mg/ml of collagenase I (C-0130; Sigma-Aldrich) in PBS and placed in a water bath at 37°C for 30 min. The digestion was then stopped by adding an equal volume of 10% FBS. The solution was filtered through 70- and 40-µm strainers (352350 and 352340; Falcon) to eliminate glomeruli and undigested tubular pieces. The suspensions were then collected and centrifuged at 4°C at 2,000 rpm for 5 min. The pellets were resuspended in MACS running buffer (130-091-221; Miltenyi Biotec) and incubated with biotinylated primary anti-vimentin monoclonal antibody (Biotin-60330; Proteintech) for 30 min. The cells were subsequently incubated with Streptavidin Microbeads (130-048-102; Miltenyi Biotec) for 15 min at 4°C. Positive selection was achieved using MACS magnetic bead separation in accordance with the manufacturer’s instructions. Primary kidney fibroblasts were cultured in DMEM/F12 medium (Thermo Fisher Scientific) with the addition of 10% FBS and 1% PS (Thermo Fisher Scientific) at 37°C in a CO_2_ incubator and within two passages were subjected to various treatments as indicated.

To prepare primary peritoneal fibroblasts, mouse omentum was collected from the abdominal cavity, and excess fat was removed. The omentum was washed in PBS and divided into small segments, then digested with 1 mg/ml of collagenase I in PBS at 37°C for 60 min in a water bath. Digestion was terminated by adding an equivalent amount of 10% FBS. The suspensions were collected and centrifuged at 2,000 rpm for 5 min at 4°C. Pellets were resuspended in MACS running buffer (130-091-221; Miltenyi Biotec) and incubated for 30 min with biotinylated primary anti-vimentin antibody (Biotin-60330; Proteintech). The cells were then treated with Streptavidin Microbeads (130-048-102; Miltenyi Biotec) for 15 min at 4°C. Positive selection was performed utilizing MACS magnetic bead separation according to the manufacturer’s recommendations. Primary peritoneal fibroblasts were cultured in DMEM/F12 medium (Thermo Fisher Scientific) supplemented with 10% FBS and 1% PS (Thermo Fisher Scientific) at 37°C in a CO_2_ incubator. Cells within two passages were used for indicated treatments.

To prepare primary skin fibroblasts, shaved and depilated mouse dorsal skin samples were harvested, defatted, and dissected into ∼1.0 cm^2^ pieces using sterile scissors. Tissue fragments were washed thoroughly in PBS, then submerged epidermis-side up in 0.25% trypsin (15050057; Gibco) at 4°C overnight, followed by 37°C for 30 min to remove the epidermis. After discarding the epidermis, the retained dermis was incubated in 0.05% trypsin/EDTA (25300062; Gibco) at 37°C for 15 min (with careful aspiration every 5 min). The resulting cell suspension was transferred to serum-containing medium to terminate digestion and centrifuged at 1,000 rpm for 5 min at 4°C. Pellets were resuspended in MACS running buffer (130-091-221; Miltenyi Biotec) and incubated for 30 min with biotinylated primary anti-vimentin antibody (Biotin-60330; Proteintech). The cells were then treated with Streptavidin Microbeads (130-048-102; Miltenyi Biotec) for 15 min at 4°C. Positive selection was performed utilizing MACS magnetic bead separation according to the manufacturer’s recommendations. Primary skin fibroblasts were resuspended in DMEM/F12 medium (Thermo Fisher Scientific) supplemented with 10% FBS and 1% PS (Thermo Fisher Scientific). Cells were cultured at 37°C in a CO_2_ incubator, and those within two passages were used for experiments.

### Cell sorting and treatment

To sort E-cadherin^+^ renal TECs, CD45^+^ immune cells, and vimentin^+^ renal fibroblasts, kidney tissues from sham or UUO mice were collected and minced. The fragments were digested with 0.25 mg/ml Liberase TL (5401020001; Roche) at 37°C for 45 min, and the reaction was stopped by adding an equal volume of 10% FBS. The suspension was filtered through 100-µm strainers (352360; Falcon) and centrifuged at 1,500 rpm for 5 min at 4°C. The cell pellet was resuspended in FACS buffer (PBS with 2% FBS) and stained with a viability dye (423106; BioLegend) for 8 min, followed by washing. Cells were then incubated with anti-CD16/32 antibody (101319; BioLegend) for 15 min on ice to block Fc receptors. Subsequently, cells were stained with fluorescence-conjugated antibodies against CD45 (157219; BioLegend), E-cadherin (147312; BioLegend), and vimentin (S0B0141; Starter) on ice for 20 min. After washing and passing through a 70-µm cell strainer, viable cells were gated; the following populations were sorted using a BD FACSAria III cell sorter (BD Biosciences): renal immune cells (CD45^+^), renal epithelial cells (CD45^−^ E-cadherin^+^), and renal fibroblasts (CD45^−^ E-cadherin^−^ vimentin^+^). Total RNA was extracted from sorted cells for RT-PCR analysis.

To isolate splenic neutrophils, single-cell suspensions were prepared by mechanical dissociation of spleen from *Ifnlr1*^*f/f*^ mice and *Ifnlr1*^*f/f*^; *Col1a2-*Cre mice. Erythrocytes were removed by treatment with ACK lysis buffer (0.15 mM NH_4_Cl, 10 mM KHCO_3_, and 0.1 mM EDTA-Na_2_ in Milli-Q water). Cells were then resuspended in FACS buffer (PBS with 2% FBS) and incubated with anti-mouse CD16/32 antibody (101319; BioLegend) on ice for 15 min to block Fc receptors. Subsequently, cells were stained with fluorescence-conjugated antibodies against CD45 (157219; BioLegend), CD11b (101208; BioLegend), and Ly6G (127622; BioLegend) on ice for 20 min. After washing and passing through a 70-µm cell strainer, cells were resuspended in FACS buffer and sorted on a BD FACSAria III cell sorter (BD Biosciences). Viable cells were gated, and CD45^+^CD11b^+^Ly6G^+^ neutrophils were purified to a purity of >97%. Purified neutrophils were then treated with 100 ng/ml of IFN-λ2 or PBS for 1 h or 24 h. STAT1 activation were assessed by western blotting, and the ISG expression was quantified by RT-qPCR.

### Culture of MEFs

MEFs were purchased from the Cell Bank of the Chinese Academy of Sciences (Shanghai, China). MEFs were cultured in MEF medium (11095080; Thermo Fisher Scientific) supplemented with 10% FBS and 1% PS at 37°C in a CO_2_ incubator and used within two passages for the appropriate treatments.

### *In vitro* primary renal TEC treatment

Primary renal TECs were cultured in 6-well plates until reaching 80% confluency. Cells were pretreated with 2.5 mM Cyclo (Phe-Pro, cFP) (HY-P1934; MedChemExpress), a cFP, or vehicle control for 6 h, followed by stimulation with 1 μg/ml of poly(I:C) (InvivoGen, tlrl-picwlv) for 12 h according to the manufacturer’s instructions. Cell culture supernatant for ELISA detection, total RNA was extracted for RT-qPCR, and protein was isolated for western blot analysis.

Primary renal TECs isolated from WT and *Mavs*^*–/–*^ mice were transfected with or without 1 μg/ml of poly(I:C) (InvivoGen, tlrl-picwlv) for 12 h. Cellular responses were subsequently assessed by RT-qPCR and western blot analysis.

Primary renal TECs isolated from *Ifnlr1*^*f/f*^ mice and *Ifnlr1*^*f/f*^; *Col1a2-*Cre mice were treated with 100 ng/ml of IFN-λ2 or PBS for 1 h or 24 h. STAT1 activation were assessed by western blotting. The expression of ISGs was quantified by RT-qPCR.

### *In vitro* primary fibroblast and MEF treatment

Primary renal fibroblasts isolated from WT and *Ifnlr1*^*−/−*^ mice were stimulated with or without 100 ng/ml IFN-λ2 (250-33; PeproTech) for 1 h or 24 h. In separate experiments, primary renal and skin fibroblasts from WT mice were treated with 100 ng/ml of IFN-λ2 (250-33; PeproTech), IFN-α (CK83; Novoprotein), IFN-β (HY-P73130; MedChemExpress), or IFN-γ (315-05; PeproTech) for different times. Western blot was used for protein validation, and RT-qPCR was used to quantify target transcripts. In parallel, conditioned media were collected for subsequent transwell migration assays.

Primary renal fibroblasts isolated from WT and *Ifnlr1*^*−/−*^ mice were treated with or without 100 ng/ml IFN-λ2 (250-33; PeproTech) for 2 h. ERK and JNK activation were assessed by western blotting. For inhibitor studies, primary renal fibroblasts isolated from WT mice were pretreated with or without 1 μM ERK inhibitor SCH772984 (HY-50846; MedChemExpress) or 10 μM JNK inhibitor SP600125 (HY-12041; MedChemExpress), followed by stimulation with or without 100 ng/ml IFN-λ2. Protein levels were assessed by western blotting, and target gene expression was quantified by RT-qPCR.

Primary renal fibroblasts from WT mice were treated with 100 ng/ml of IFN-λ2 (250-33; PeproTech) or PBS, in combination with either 2 μg/ml of TGF-β–neutralizing antibody (16-9243-85; Thermo Fisher Scientific) or anti-IgG1 isotype control antibody (02-6100; Thermo Fisher Scientific), for 24 h. Then, RNA was extracted for RT-qPCR, and protein was isolated for western blot analysis.

Primary peritoneal fibroblasts from WT mice and MEFs were stimulated with or without 100 ng/ml of IFN-λ2 (250-33; PeproTech) for 24 h. Then, RNA was extracted, and the expression of ISGs was quantified by RT-qPCR.

### Indirect co-culture model of renal TECs and fibroblasts

To determine whether poly(I:C)-induced IFN-λ production is required for renal fibroblast activation, primary renal TECs and fibroblasts were seeded in 6-well plates at a density of 1 × 10^6^ cells per well at 37°C in a CO_2_ incubator. Renal TECs from WT mice were treated with or without 1 μg/ml poly(I:C) (InvivoGen, tlrl-picwlv) for 12 h, then cell culture supernatants were mixed with fresh medium at a ratio of 1:1 as conditional medium. Fibroblasts from WT or *Ifnlr1*^*−/−*^ mice were washed with PBS and cultured in conditional medium with the presence or absence of 10 μg/ml of anti–IFN-λ2/3 antibody (MAB17892; R&D System) or IgG2b isotype control (MAB0061; R&D System) for 24 h, followed by either RNA isolation for RT-qPCR analysis or protein extraction for western blot assessment. Some conditional medium was employed for the subsequent cell transwell migration studies.

To investigate whether MAVS-mediated IFN-λ production is necessary for renal fibroblast activation, renal TECs and fibroblasts were seeded in 6-well plates at a density of 1 × 10^6^ cells per well at 37°C in a CO_2_ incubator. Renal TECs from WT or *Mavs*^*–/–*^ mice were treated with or without 1 μg/ml of poly(I:C) for 12 h, then cell culture supernatants were mixed with fresh medium at a ratio of 1:1 as conditional medium. Renal fibroblasts from WT mice were washed with PBS and cultured in conditional medium for 24 h, followed by either RNA isolation for RT-qPCR analysis or protein extraction for western blot assessment. Some conditional medium was used for subsequent transwell migration experiments.

### Transwell migration assay

To investigate whether renal TEC-derived IFN-λ is necessary for renal fibroblast migration, renal fibroblasts isolated from WT mice were serum-deprived for 4 h in six-well plates. Renal fibroblasts were resuspended at a concentration of 1 × 10^5^ cells per well, with 200-μl aliquots seeded into the upper chamber of an 8-μm pore size transwell system (3422; Corning). The lower chamber was loaded with conditioned medium derived from differentially treated renal TECs (treatment groups detailed in corresponding figures). After 24 h of incubation, the culture medium in the lower chamber of the Transwell was aspirated, the chamber was washed twice with PBS, and the cells were fixed with 4% paraformaldehyde (PFA, P1110; Solarbio) at room temperature for 30 min. Subsequently, the residual fibroblasts on the upper chambers were gently removed with a cotton swab. Then, 0.1% crystal violet was used to stain the cells in the lower chamber for 30 min at room temperature. Finally, the stained renal fibroblasts were examined using an optical microscope (Olympus Optical, Olympus BX-50), and the total cell number was counted.

### RT-qPCR

Total RNA was extracted from cells and tissues using the Trizol reagent RNAiso Plus (9109; Takara), according to the manufacturer’s procedure. The extracted RNA was reverse transcribed into cDNA using the RevertAid First Strand cDNA Synthesis Kit (K1622; Thermo Fisher Scientific). Following reverse transcription, qPCR amplification was carried out using a CFX96 Real-Time PCR System (Bio-Rad) and Tip Green qPCR SuperMix (AQ141-01; TransGen). Relative fold changes in target gene mRNA levels were measured in cells or tissues using the 2^−ΔΔCt^ method and normalized to *Gapdh* (mouse) or actin β (*ACTB*) (human) expression. The primer sequences used for PCR amplification are listed in Tables S1 and S2.

### ELISA

Cell culture supernatants were harvested and tested for IFN-λ2/3 and TGF-β production using a mouse IFN-λ2/3 ELISA kit (EM562RB; Invitrogen) and TGF-β ELISA kit (BMS608-4; Invitrogen), respectively, according to the manufacturer’s protocols. Cellular dsRNA was measured by a dsRNA ELISA kit (36717ES; Yeasen), according to the manufacturer’s protocols. The OD of each sample at wavelengths of 450 nm was measured by the BioTek Epoch 2 Microplate Spectrophotometer (Agilent).

### Western blot

Cells and tissues were collected and lysed with RIPA buffer (R0010; Solarbio) containing protease inhibitor (05892791001; Roche) and phosphatase inhibitor (04906837001; Roche) to extract proteins. The protein concentration was determined with the BCA assay kit (P0012; Beyotime). Protein samples were separated by sodium dodecyl sulfate-PAGE (SDS-PAGE) with the Omni-Easy one-step PAGE Gel Fast Preparation Kit (PG222; Epizyme) and transferred to nitrocellulose membranes. The membrane was washed and blocked in TBS plus Tween (TBST) (1 × TBS with 0.05% Tween-20) supplemented with 5% skim milk powder for 1 h at room temperature with gentle shaking, then incubated overnight at 4°C with the following primary antibodies: anti-IFN-λ2/3 (ab191426; Abcam), anti-α-SMA antibody (ab5694; Abcam), anti-fibronectin antibody (ab2413; Abcam), anti-vimentin (A19607; ABclonal), p-STAT1 (9167; Cell Signaling Technology), STAT1 (66545-1-Ig; Proteintech), TGF-β (81746-2-RR; Proteintech), p-SMAD2 (18338; Cell Signaling Technology), p-SMAD3 (9520; Cell Signaling Technology), SMAD2/3 (8685; Cell Signaling Technology), SMAD4 (38454; Cell Signaling Technology), SMAD7 (25840-1-AP; Proteintech), RIG-I (ab302778; Abcam), MAVS (A25005; ABclonal), p-IRF3 (AP0623; ABclonal), IRF3 (MA5-32348; Invitrogen), p-ERK (9101; Cell Signaling Technology), ERK (9102; Cell Signaling Technology), p-JNK (9251; Cell Signaling Technology), JNK (9252; Cell Signaling Technology), p-mTOR (2971; Cell Signaling Technology), mTOR (2983; Cell Signaling Technology), p-p38 (9215; Cell Signaling Technology), p38 (9212; Cell Signaling Technology), p-PI3K (17366; Cell Signaling Technology), PI3K (4257; Cell Signaling Technology), MDA5 (ab315242; Abcam), p-STING (72971; Cell Signaling Tehcnology), STING (13647; Cell Signaling Technology), TLR3 (HA724070; HUABIO), Myd88 (AF7524; Beyotime), and GAPDH (81640-5-RR; Proteintech). The membrane was rinsed three times for 30 min in TBST before being incubated with HRP-conjugated secondary antibodies for 1 h at room temperature. After three washes, the membrane was transferred to the BeyoECL Star reagent (P0018AM; Beyotime). The images were obtained by the Tanon 5200 multi-gel imaging system (Tanon). GAPDH was employed as the loading control protein.

### Histological analysis

Kidney tissue samples were fixed in 4% PFA (P1110; Solarbio) overnight at 4°C, embedded in paraffin, and sectioned to 4 μm thickness. The sections were placed on glass slides, deparaffinized with xylene, and then dehydrated using a graded series of ethanol. Kidney sections were stained with Masson’s trichrome (G1006; ServiceBio) and PSR (G1078; ServiceBio) to visualize the fibrotic matrix. Sections were examined using an optical microscope (Olympus Optical, Olympus BX-50). To quantify the fibrotic area following Masson’s trichrome and PSR staining, the staining area in 10 random fields of view was calculated using ImageJ version 1.52n analysis software. Data are expressed as the percentage of positively stained area to the total analyzed area. All samples were evaluated in a blind manner.

### IHC analysis

The paraffin-embedded kidney tissue samples were sectioned into a thickness of 4 μm, deparaffinized, rehydrated, and then antigen-retrieved. Subsequently, these sections were blocked with 1% BSA blocking solution for 20 min. The sections were incubated with primary antibodies against α-SMA (ab5694; Abcam), fibronectin (ab2413; Abcam), p-Smad2/3 (AP0548; ABclonal), or IFN-λ2/3 (ab191426; Abcam) overnight at 4°C, respectively. After washing in PBS, slides were treated with the corresponding HRP-conjugated secondary antibody (PV-6001; Zsbio) followed by the DAB Detection Kit (ZLI-9019; Zsbio). Images were obtained using an optical microscope (Olympus Optical, Olympus BX-50). The mean OD value across 10 randomly selected regions within each sample of DAB-positive reactants was detected by ImageJ version 1.52n software. All samples were evaluated in a blind way. Data are presented as the relative mean OD of identified protein molecules in the kidney tissues.

### Immunofluorescence staining

The kidney tissues were fixed in paraffin and sectioned to a thickness of 4 μm. The slices were incubated with dimethyl benzene for 15 min before being treated with 100%, 85%, and 75% ethanol for 5 min, respectively. Deparaffinized tissue slices were treated to antigen rederivation using EDTA buffer, followed by blocking with 1% BSA before incubation with primary antibodies against E-cadherin (GB12083; ServiceBio), IFN-λ2/3 (ab191426; Abcam), J2 (76651; Cell Signaling Technology), or p-SMAD2/3 (AP0548; ABclonal) overnight at 4°C. The slides were then washed three times with PBS before being incubated with an FITC-conjugated goat anti-mouse secondary antibody (F-2761; Thermo Fisher Scientific), an Alexa Fluor 594–conjugated goat anti-rabbit secondary antibody (A-11012; Thermo Fisher Scientific), or an Alexa Fluor 488–conjugated goat anti-rabbit secondary antibody (A-11008; Thermo Fisher Scientific) for 30 min at room temperature, followed by three washes with PBS. Finally, the nuclei were stained with DAPI solution (G1012; ServiceBio). Images were obtained using light microscopy (Olympus Optical, Olympus BX-50). To evaluate the percentage of protein molecules and cells, 10 views per slide were randomly selected and were analyzed with ImageJ (version 1.52n) analysis software.

### Multiplex immunofluorescence staining

Formalin-fixed paraffin-embedded kidney tissues were cut to 4 μm thickness. These sections were deparaffinized with xylene, rehydrated with decreasing concentrations of ethanol in water, and performed using a microwave-based antigen retrieval technique. After being blocked with 1% BSA for 20 min, the sections were incubated overnight at 4°C with primary antibodies against IFN-λ2/3 (ab191426; Abcam), E-cadherin (GB12083; ServiceBio), CD45 (103101; BioLegend), and Vimentin (A19607; ABclonal), respectively. The slides were detected by a HRP-conjugated goat anti-rabbit secondary antibody (GB23303; ServiceBio) or HRP-goat anti-mouse secondary antibody (GB23301; ServiceBio) followed by TSAPlus Fluorescence 4-Label 5-Color Staining Kit (G1255; ServiceBio) at a 1:500 dilution for 15 min. The kit contains the TSA fluorophores Opal 440, Opal 546, Opal 647, and Opal 750. Finally, nuclei were stained with DAPI (G1012; ServiceBio), and the slides were mounted with Anti-Fade Fluorescence Reagent (AR1109; Boster Biological Technology). Multispectral images were acquired using the Vectra 3.0 System (PerkinElmer), which captured fluorescent spectra images in five channels (DAPI, Opal 440, Opal 546, Opal 647, and Opal 750). Images were processed using ImageJ version 1.52n analysis software to determine the proportion of distinct cell types expressing IFN-λ in kidney tissues.

### Analysis of scRNA-seq datasets

Publicly available scRNA-seq datasets (GSE211785, GSE145173, GSE198621, GSE197266) were obtained and reprocessed using a standard workflow in Scanpy (Python, version 1.11.5) and Seurat (R, version 4.4.0). Briefly, cells were filtered using standard quality-control metrics to remove low-quality profiles as described in the original references. The filtered count matrices were then normalized by total-count normalization per cell (total-count = 10^5^) and log-transformed for downstream visualization and expression comparisons, following the general strategy of the original studies (Abedini et al., 2024; Kuppe et al., 2021; Rudman-Melnick et al., 2024; Wang et al., 2022). Cell clustering and the initial identification of fibroblast populations were based on the references, using the author-provided cell type annotations when available. To confirm fibroblast identity in our reanalysis, we examined the expression of canonical fibroblast marker genes (e.g., COL1A1, COL1A2, DCN, LUM, and COL3A1) within the annotated fibroblast population.

### Statistical analysis

Statistical analysis and graph generation were performed using GraphPad Prism software (version 9.0). Data are shown as means ± SEM. Data are analyzed using unpaired two-tailed Student’s *t* test, one-way ANOVA with Tukey’s multiple-comparison test, two-way ANOVA with Tukey’s multiple-comparison test, and Pearson correlation, as described in the relevant figure legends. A P value <0.05 was considered significant.

### Online supplemental material

Fig. S1 establishes a clinical correlation by showing the relationship between renal IFN-λ expression and fibrosis. Fig. S2 demonstrates that IFN-λ activates the STAT1 signaling pathway in renal fibroblasts. Fig. S3 details the generation and validation of mice with a fibroblast-specific conditional knockout of IFN-λ receptor. Fig. S4 highlights the unique role of type III IFN by comparing its effects on TGF-β expression and ERK/JNK signaling with those of type I and II IFNs. Fig. S5 shows that inhibiting the STING, Myd88, TLR3, or RIG-I/MAVS pathways attenuates renal fibrosis in mice. Table S1 shows mouse primer sequences. Table S2 shows human primer sequences.

## Supplementary Material

Table S1 shows mouse primer sequences.

Table S2 shows human primer sequences.

SourceData F1 is the source file for Fig. 1.

SourceData F2 is the source file for Fig. 2.

SourceData F3 is the source file for Fig. 3.

SourceData F4 is the source file for Fig. 4.

SourceData F5 is the source file for Fig. 5.

SourceData F6 is the source file for Fig. 6.

SourceData F7 is the source file for Fig. 7.

SourceData F8 is the source file for Fig. 8.

SourceData FS2 is the source file for Fig. S2.

SourceData FS3 is the source file for Fig. S3.

SourceData FS4 is the source file for Fig. S4.

## Data Availability

The data underlying *IFN-λ2* and *IFN-λ3* in Fig. 1 H are openly available in the Nephroseq version 5.0 database at http://v5.nephroseq.org. The scRNA-seq data underlying Fig. 3 D are openly available in GEO at GSE145173. The scRNA-seq data underlying Fig. 3 E are openly available in GEO at GSE211785. The scRNA-seq data underlying Fig. 3 F are openly available in GEO at GSE198621. The scRNA-seq data underlying Fig. 3 G are openly available in GEO at GSE197266. All other data described in the text are available in main figures and supplementary figures.
