# Supplementary material for: Interferon-λ drives renal fibrosis by coordinating epithelial–fibroblast crosstalk
Source: J Exp Med. 2026 Jul 6;223(8):e20251858. doi: 10.1084/jem.20251858 (PMC13335421; doi:10.1084/jem.20251858)
Supplement: Table S1 — shows mouse primer sequences. [file jem_20251858_tables1.docx]

**Table S1. Mouse primer sequences**

| Gene | Sequences |
| --- | --- |
| *Ifn-λ2* (forward) | 5’- AGGTGCAGTTCCCAGCTCT -3’ |
| *Ifn-λ2* (reverse) | 5’- TCAGTCATGTTCTCCCAGACC -3’ |
| *Ifn-λ3* (forward) | 5’- TCCCAGCTGCGACCTGT -3’ |
| *Ifn-λ3* (reverse) | 5’- CAGGAGTCTCCTTGCTCTG -3’ |
| *Acta2* (forward) | 5’- GTCCCAGACATCAGGGAGTAA -3’ |
| *Acta2* (reverse) | 5’- TCGGATACTTCAGCGTCAGGA -3 |
| *Fibronectin* (forward) | 5’- ATGTGGACCCCTCCTGATAGT -3 |
| *Fibronectin* (reverse) | 5’- GCCCAGTGATTTCAGCAAAGG -3’ |
| *Vimentin* (forward) | 5’- CGTCCACACGCACCTACAG -3’ |
| *Vimentin* (reverse) | 5’- GGGGGATGAGGAATAGAGGCT -3’ |
| *Isg15* (forward) | 5’- CATCCTGGTGAGGAACGAAAGG -3’ |
| *Isg15* (reverse) | 5’- CTCAGCCAGAACTGGTCTTCGT -3’ |
| *Mx1* (forward) | 5’- TGGACATTGCTACCACAGAGGC -3’ |
| *Mx1* (reverse) | 5’- TTGCCTTCAGCACCTCTGTCCA -3’ |
| *Ifit1* (forward) | 5’- TACAGGCTGGAGTGTGCTGAGA -3’ |
| *Ifit1* (reverse) | 5’- CTCCACTTTCAGAGCCTTCGCA -3’ |
| *Oas1* (forward) | 5’- CTGTGCTGACCTCAGAGAAGTC -3’ |
| *Oas1* (reverse) | 5’- TGCCCTTGAGTGTGGTGCCTTT -3’ |
| *Tgf-β* (forward) | 5’- CTCCCGTGGCTTCTAGTGC -3’ |
| *Tgf-β* (reverse) | 5’- GCCTTAGTTTGGACAGGATCTG -3’ |
| *Gapdh* (forward) | 5’- CATCACTGCCACCCAGAAGACTG -3’ |
| *Gapdh* (reverse) | 5’- ATGCCAGTGAGCTTCCCGTTCAG -3’ |
