# Supplementary material for: Interferon-λ drives renal fibrosis by coordinating epithelial–fibroblast crosstalk
Source: J Exp Med. 2026 Jul 6;223(8):e20251858. doi: 10.1084/jem.20251858 (PMC13335421; doi:10.1084/jem.20251858)
Supplement: Table S2 — shows human primer sequences. [file jem_20251858_tables2.docx]

**Table S2. Human primer sequences**

| Gene | Sequences |
| --- | --- |
| *IFN-λ2/3* (forward) | 5’- CAAAGATGCCTTAGAAGAGTCG -3’ |
| *IFN-λ2/3* (reverse) | 5’- TCCAGAACCTTCAGCGTCAG -3’ |
| *ACTA2* (forward)  *ACTA2* (reverse)  *FIBRONECTIN* (forward) | 5’- CTATGAGGGCTATGCCTTGCC -3’  5’- GCTCAGCAGTAGTAACGAAGGA -3  5’- CGGTGGCTGTCAGTCAAAG -3 |
| *FIBRONECTIN* (reverse) | 5’- AAACCTCGGCTTCCTCCATAA -3’ |
| *ACTB* (forward) | 5’- ATCTGGCACCACACCTTC -3’ |
| *ACTB* (reverse) | 5’- AGCCAGGTCCAGACGCA -3’ |
