# Supplementary material for: Interferon-λ drives renal fibrosis by coordinating epithelial–fibroblast crosstalk
Source: J Exp Med. 2026 Jul 6;223(8):e20251858. doi: 10.1084/jem.20251858 (PMC13335421; doi:10.1084/jem.20251858)

Figure 1

Panel C

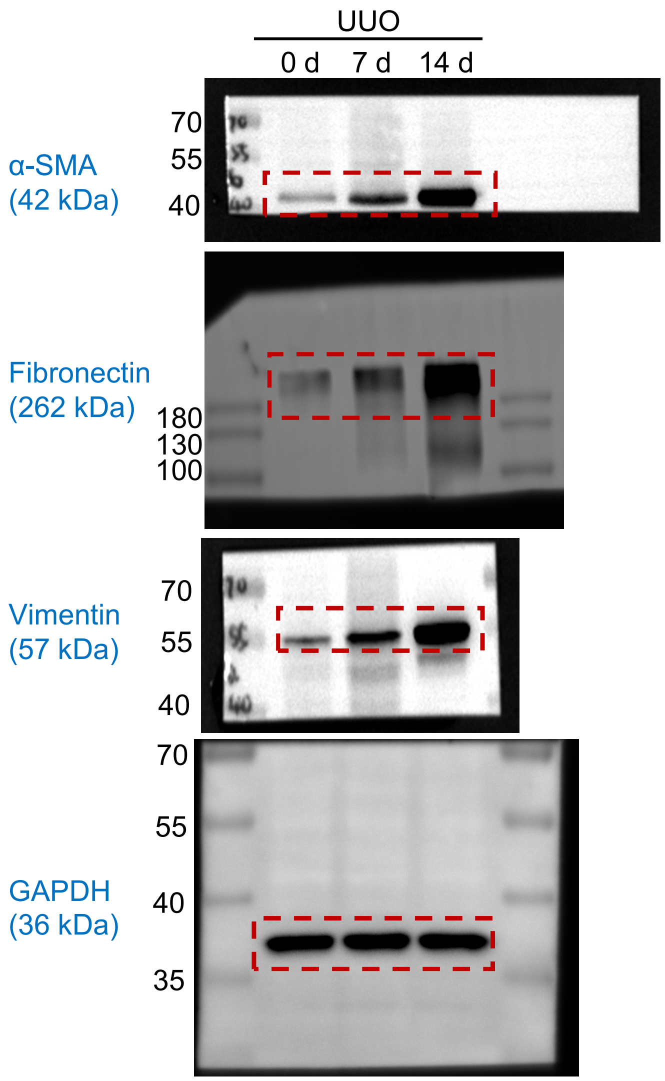

Panel F

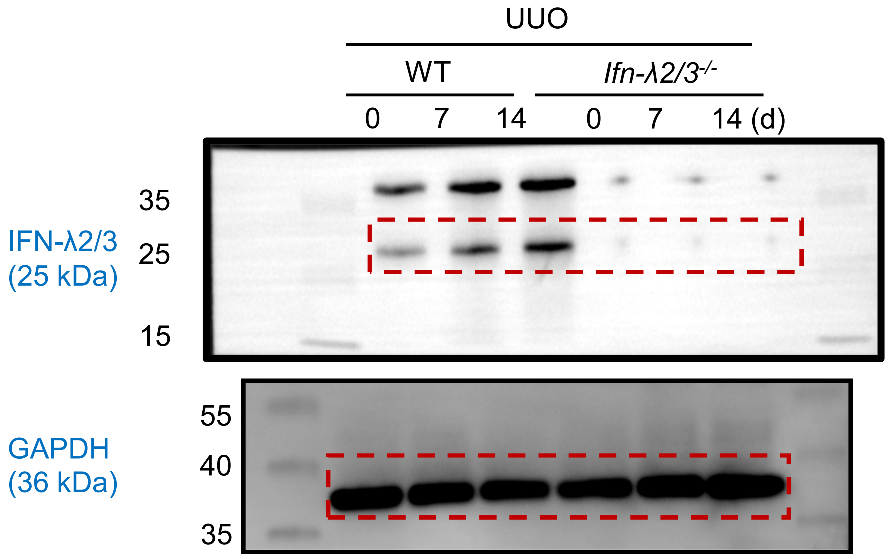

Supplement: SourceData F1 — is the source file for Fig. 1. [file jem_20251858_sourcedataf1.pdf]
