# Supplementary figures and images for: Interferon-λ drives renal fibrosis by coordinating epithelial–fibroblast crosstalk
Source: J Exp Med. 2026 Jul 6;223(8):e20251858. doi: 10.1084/jem.20251858 (PMC13335421; doi:10.1084/jem.20251858)

Figure 2

Panel D

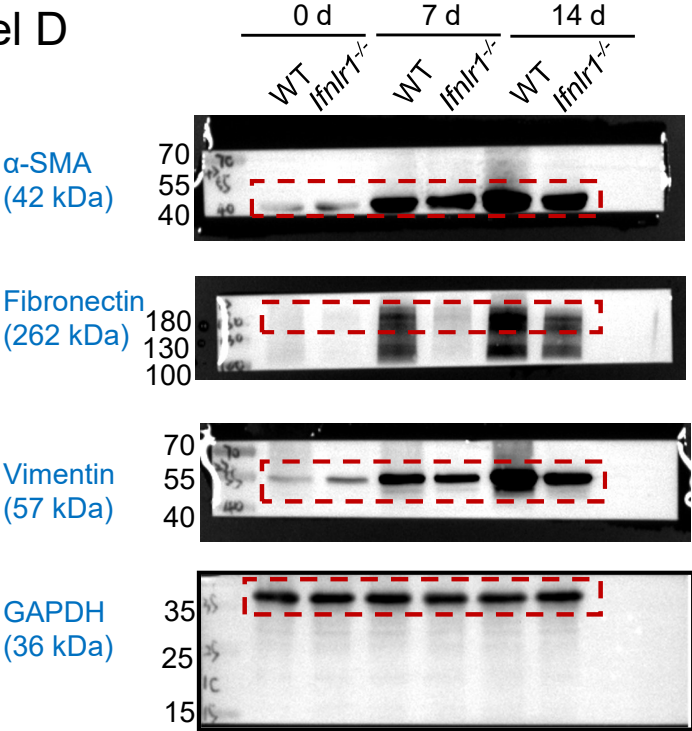

Panel J

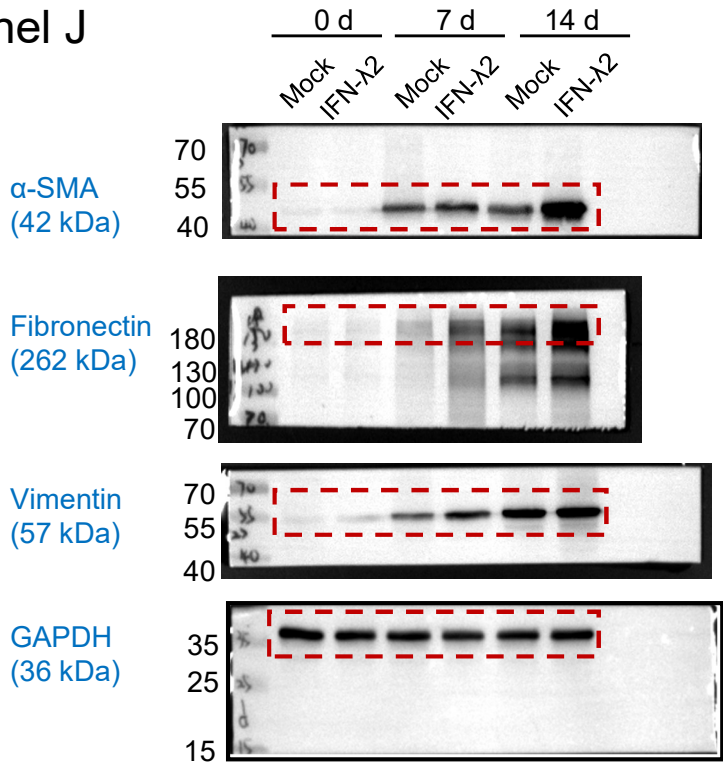

Supplement: SourceData F2 — is the source file for Fig. 2. [file jem_20251858_sourcedataf2.pdf]

Figure 3

Panel I

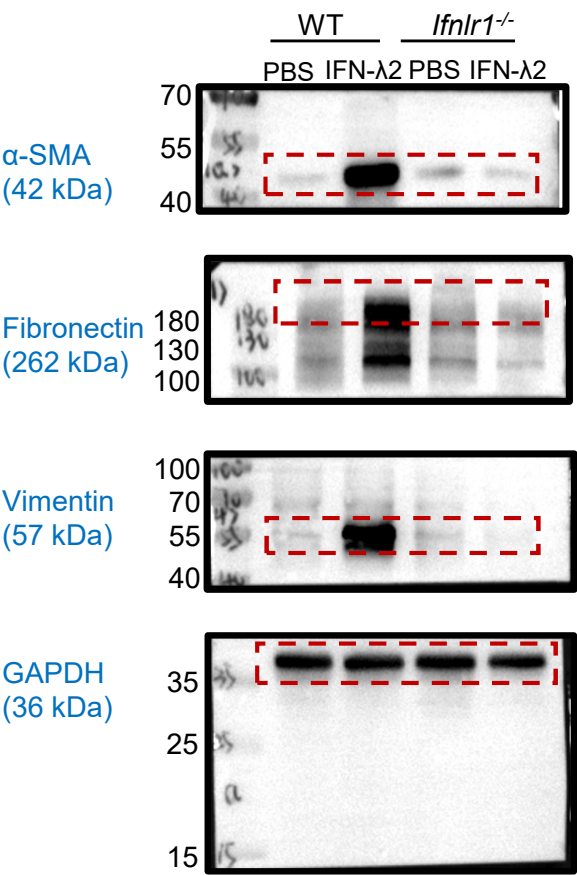

Supplement: SourceData F3 — is the source file for Fig. 3. [file jem_20251858_sourcedataf3.pdf]

Figure 4

Panel B

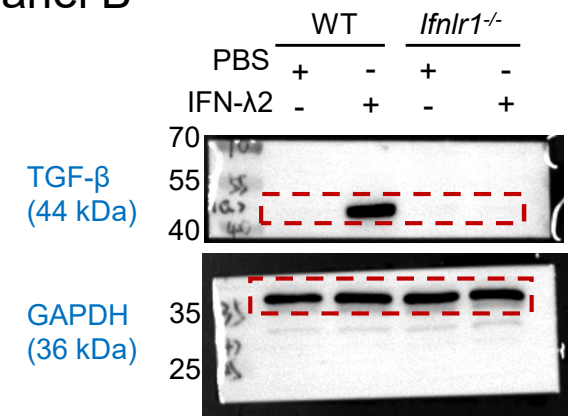

Panel C

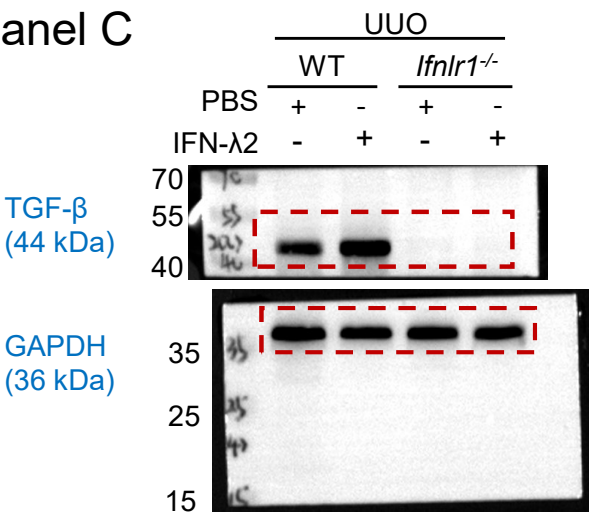

Panel D

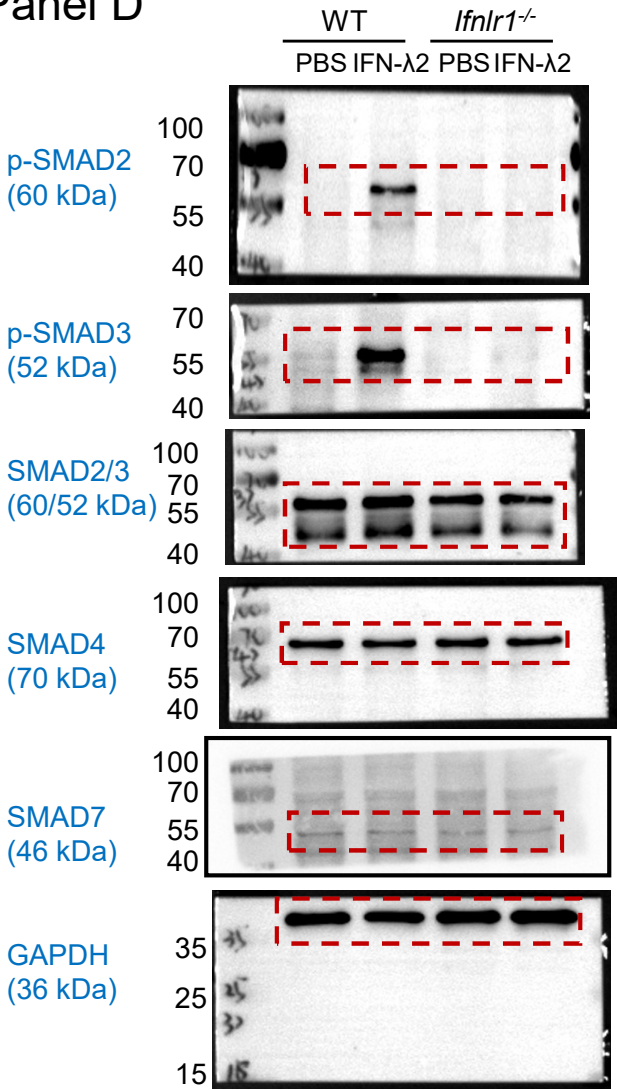

Panel E

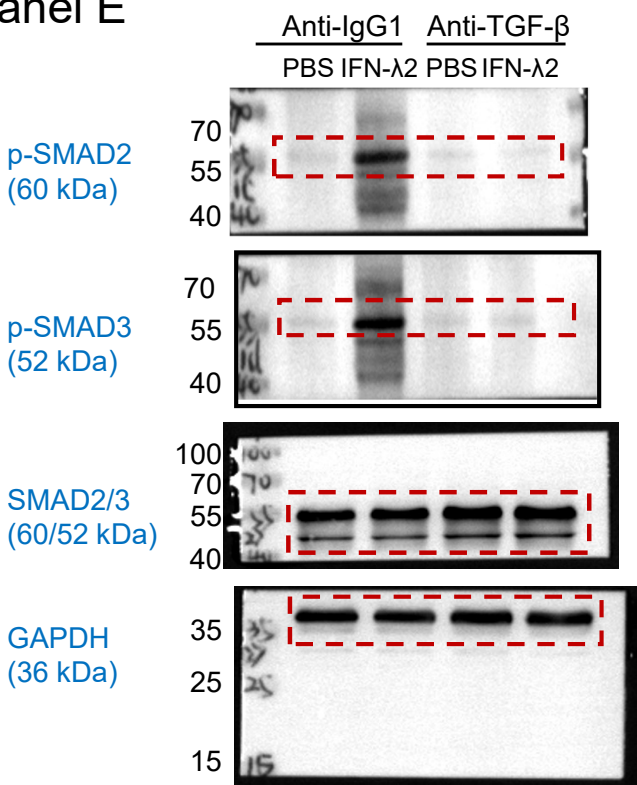

Supplement: SourceData F4 — is the source file for Fig. 4. [file jem_20251858_sourcedataf4.pdf]

Figure 5

Panel A

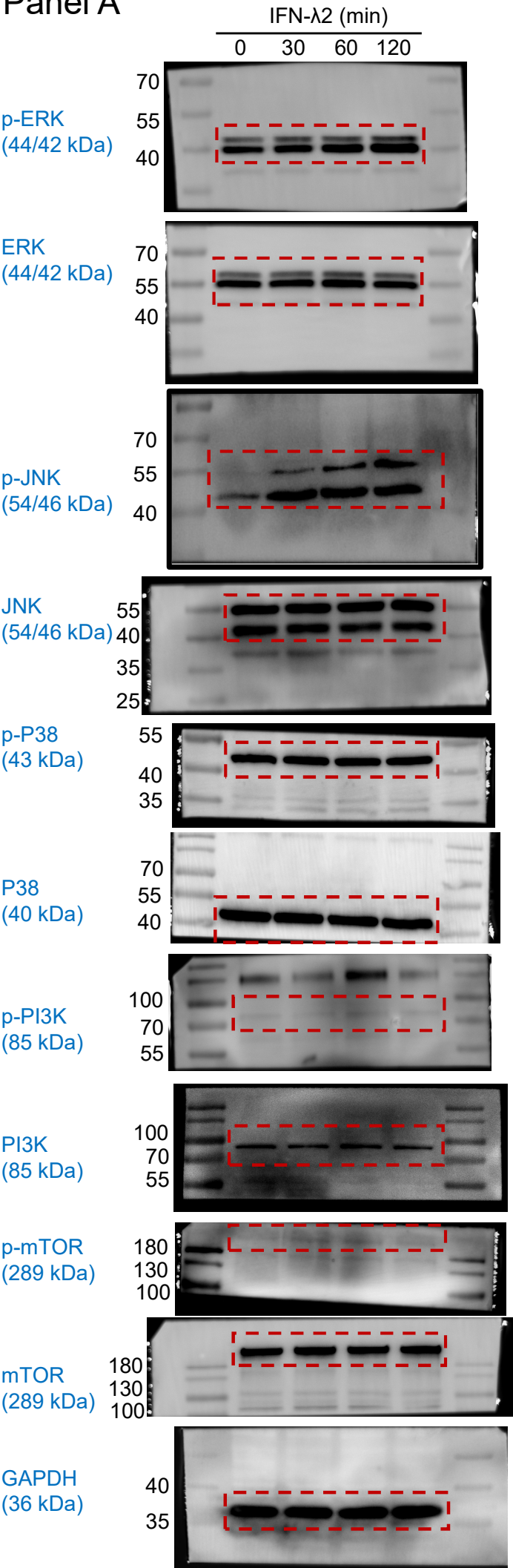

Panel B

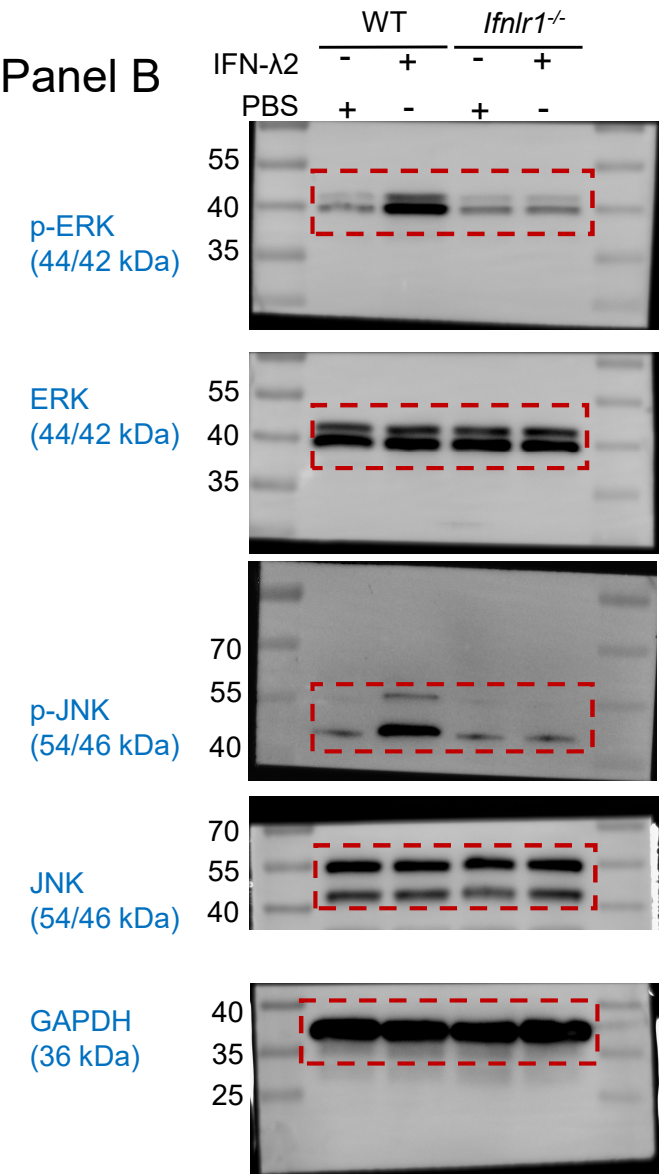

Panel C

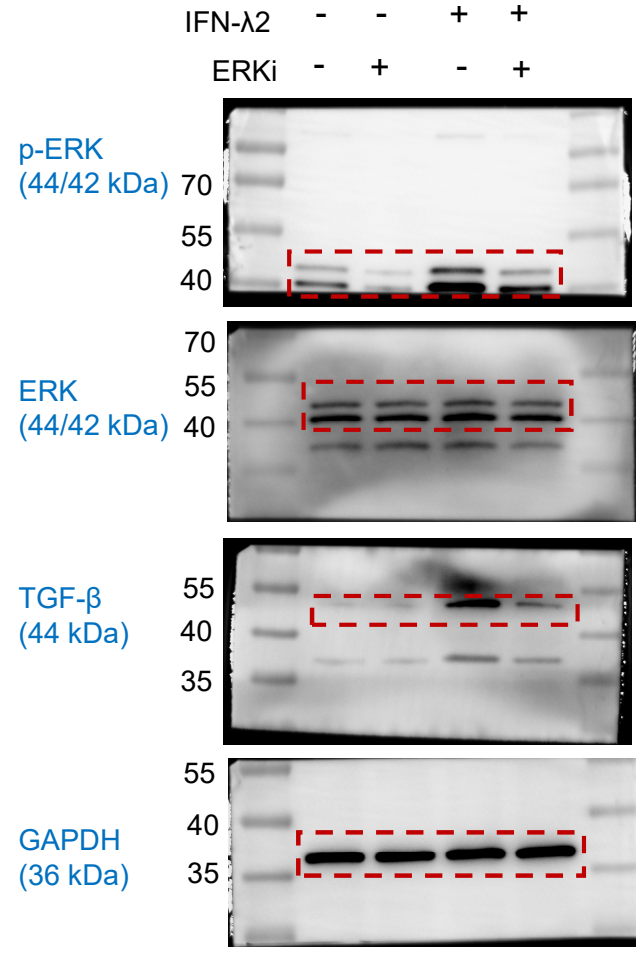

Figure 5

Panel E

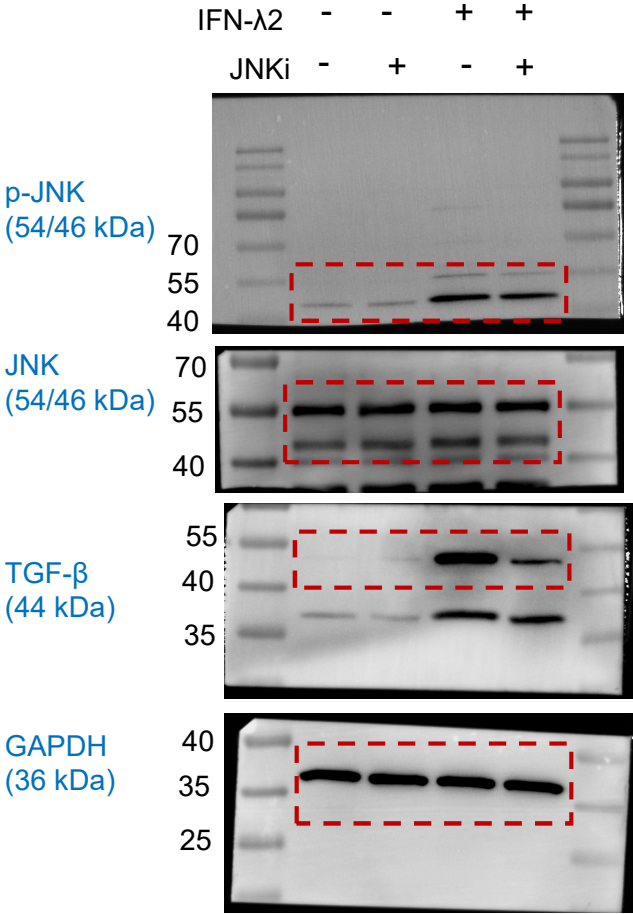

Panel H

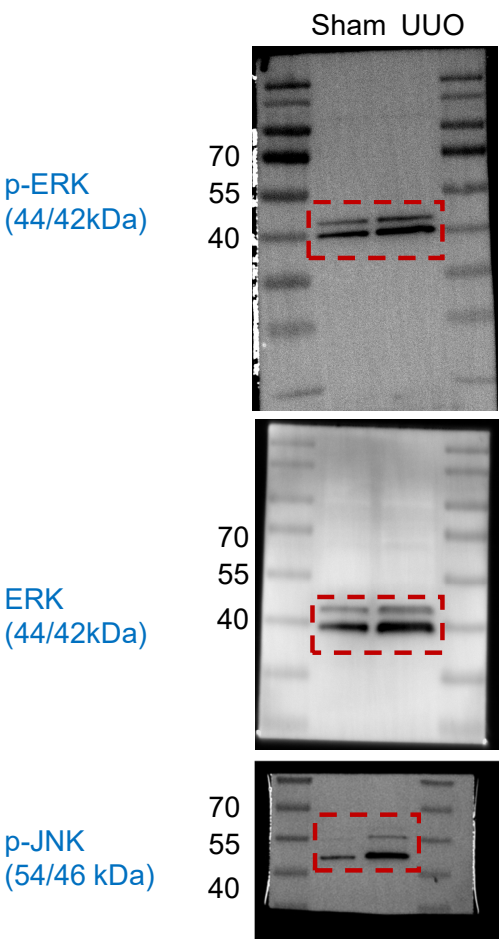

Panel I

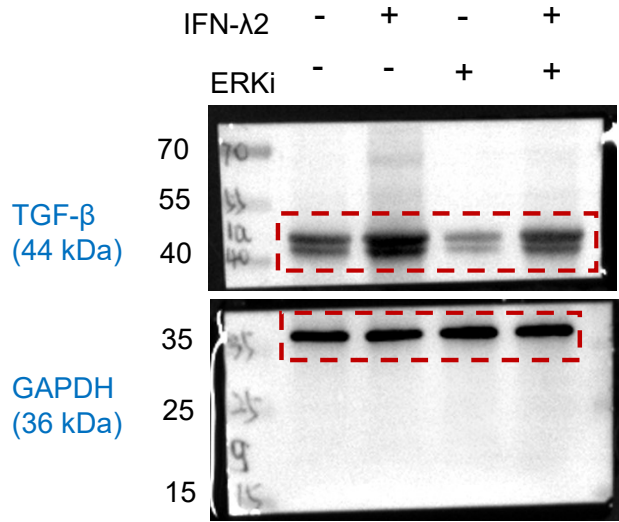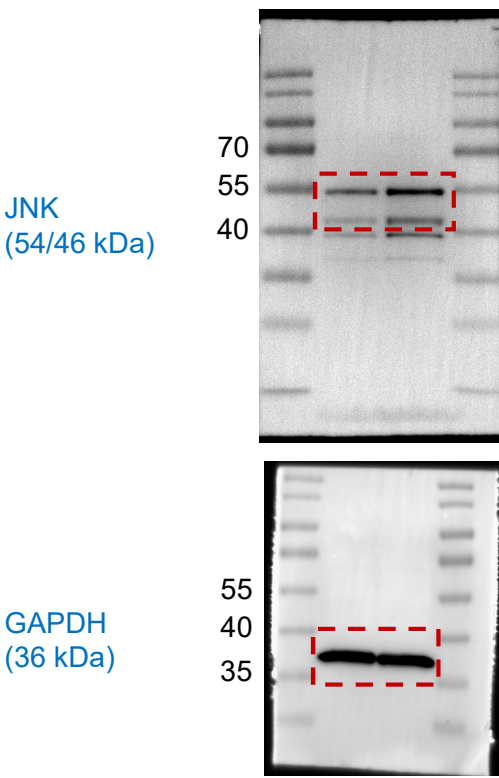

Panel J

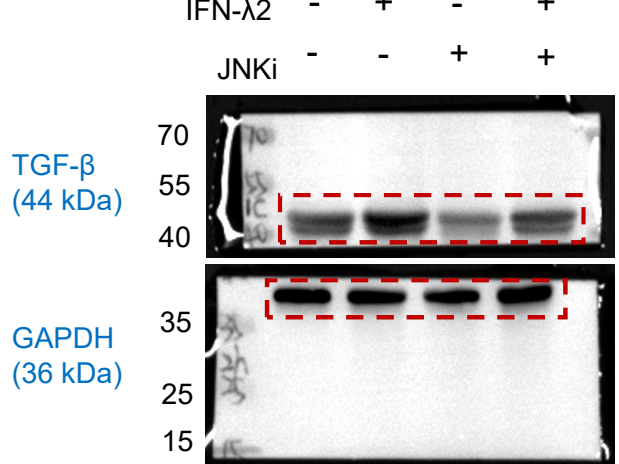

Supplement: SourceData F5 — is the source file for Fig. 5. [file jem_20251858_sourcedataf5.pdf]

Figure 6

Panel F

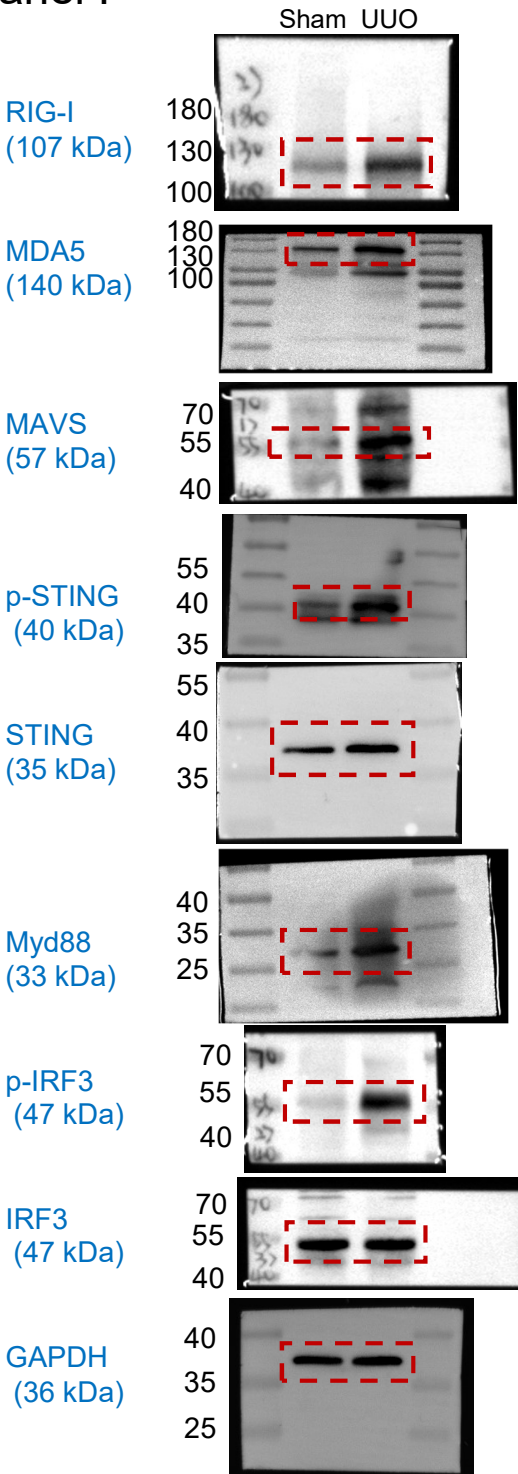

Panel I

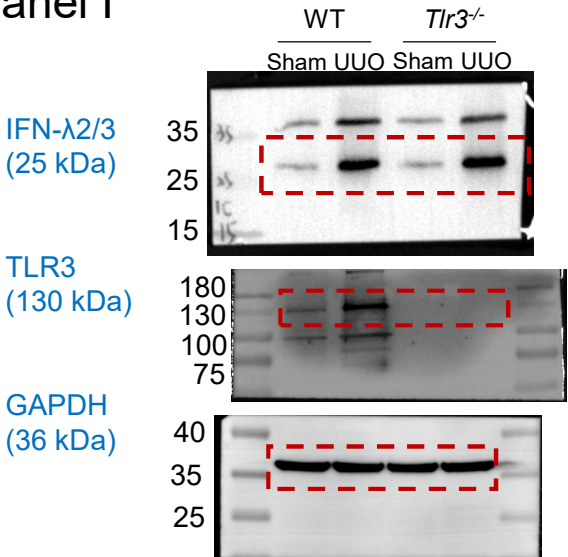

Panel G

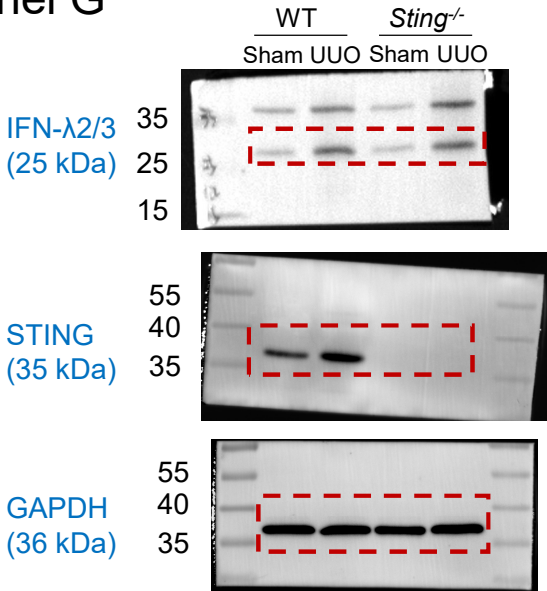

Panel H

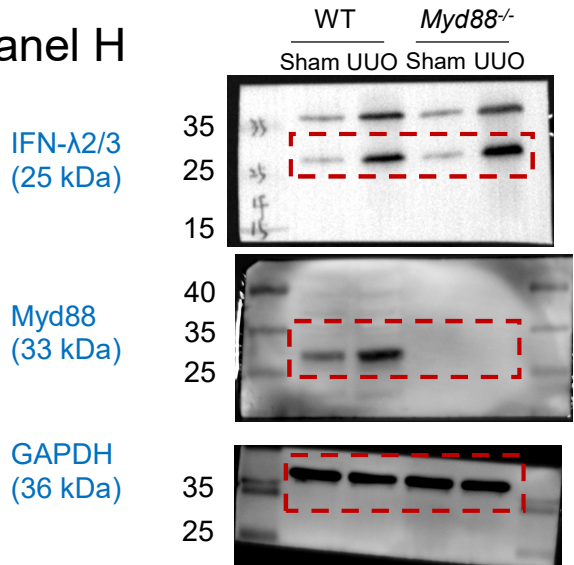

Panel J

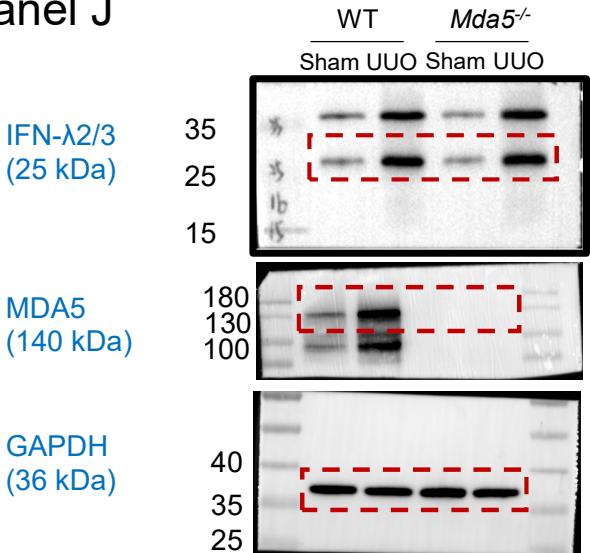

Figure 6

Panel K

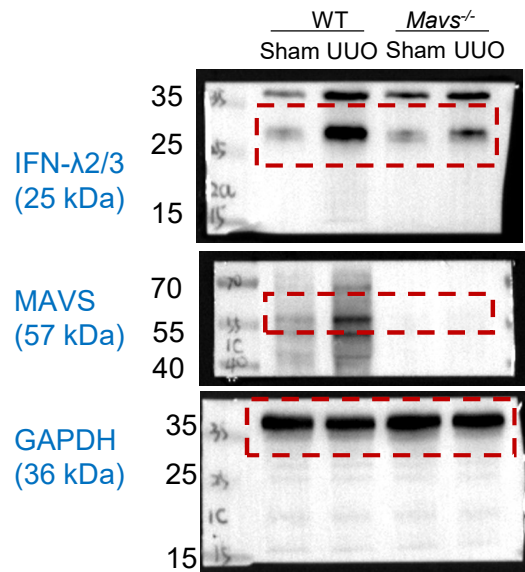

Panel L

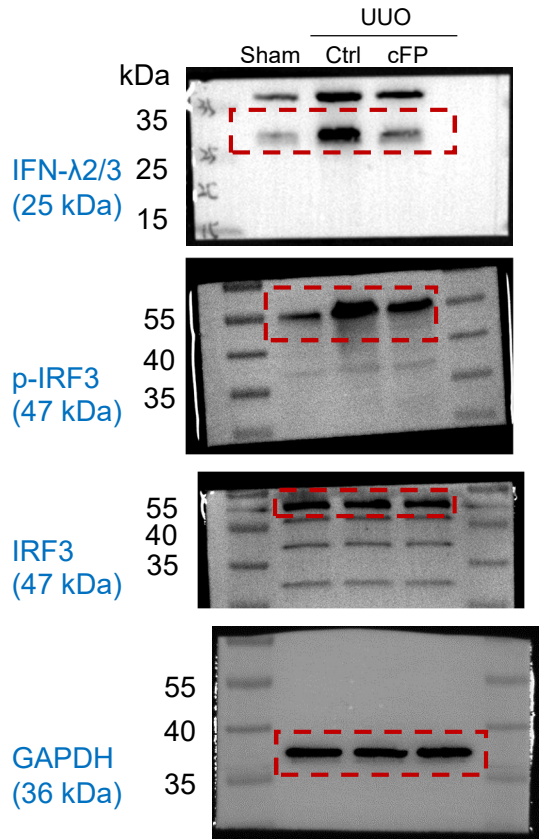

Panel Q

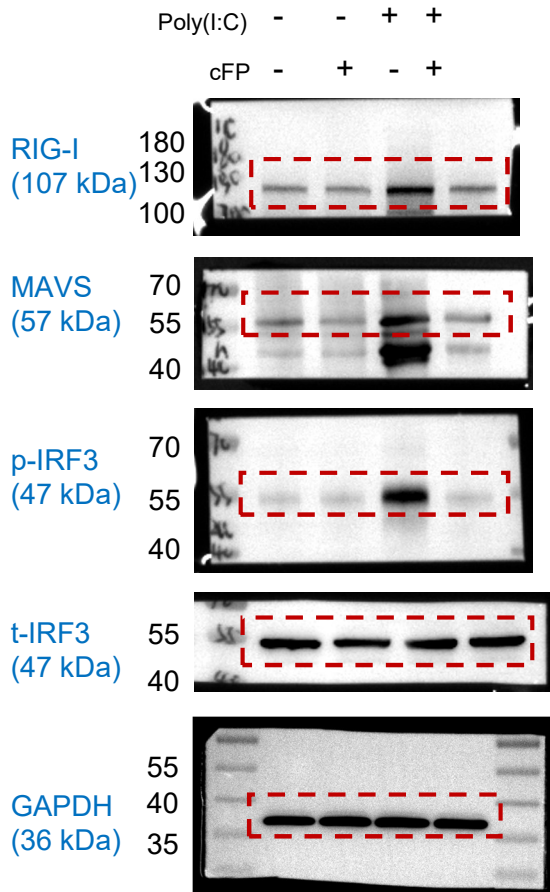

Panel S

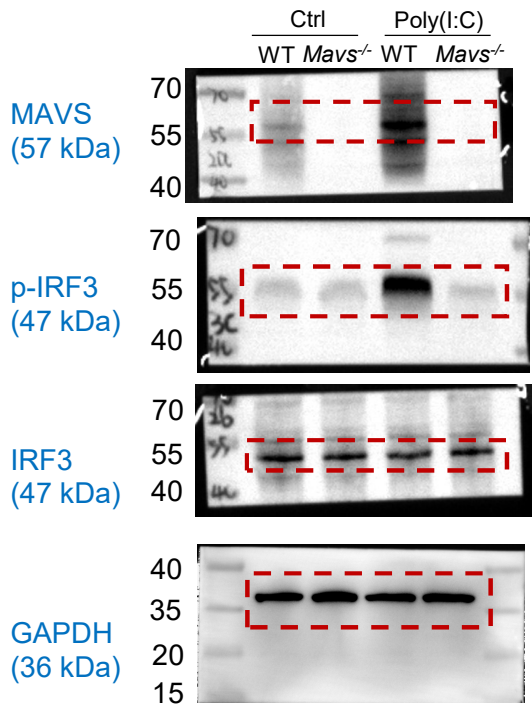

Supplement: SourceData F6 — is the source file for Fig. 6. [file jem_20251858_sourcedataf6.pdf]

Figure 7

Panel C

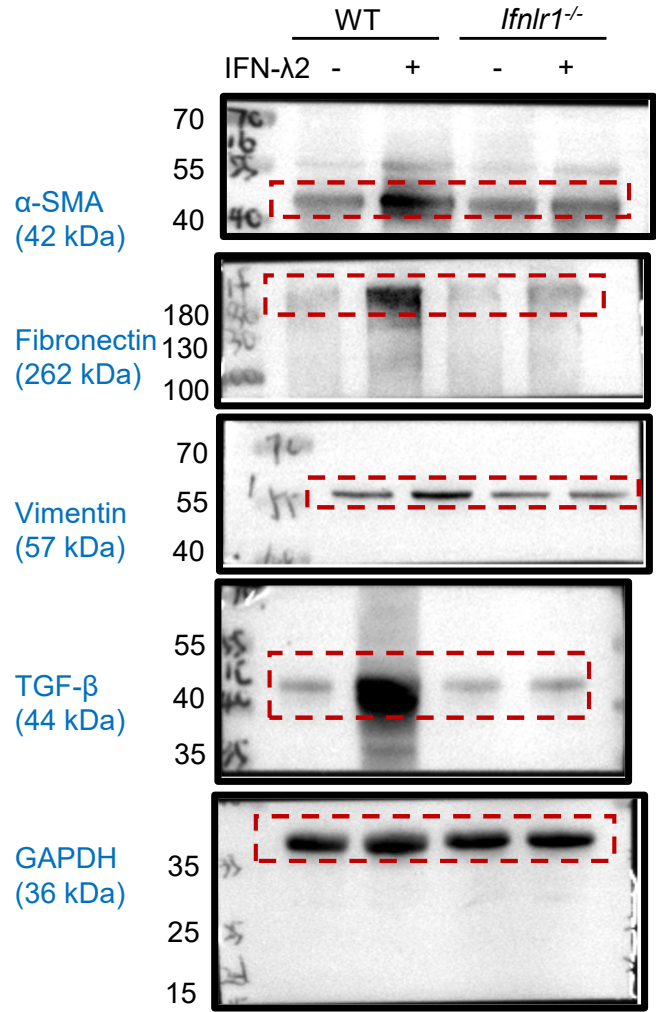

Panel G

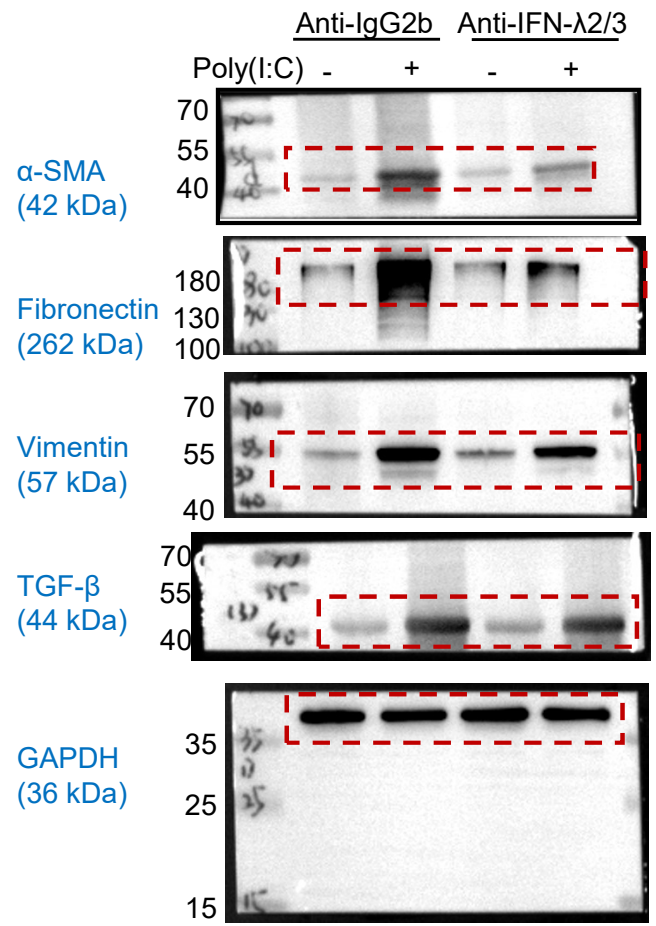

Panel K

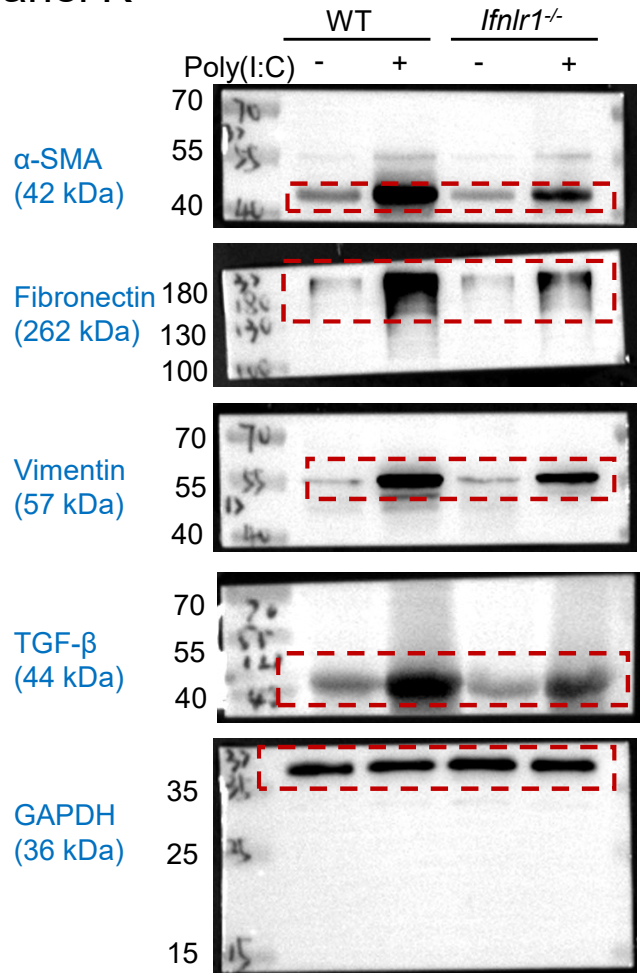

Panel O

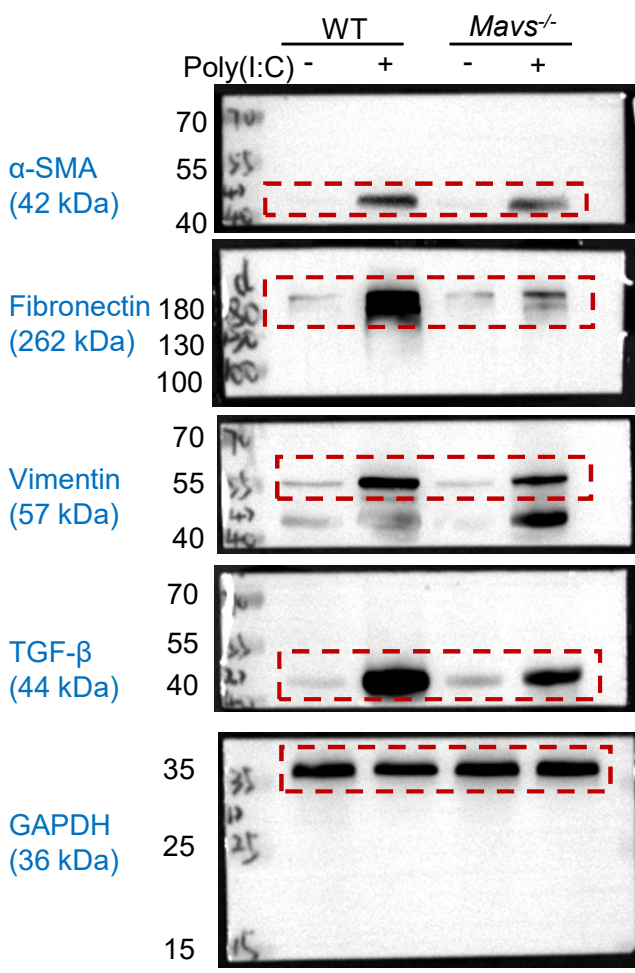

Supplement: SourceData F7 — is the source file for Fig. 7. [file jem_20251858_sourcedataf7.pdf]

Figure 8

Panel D

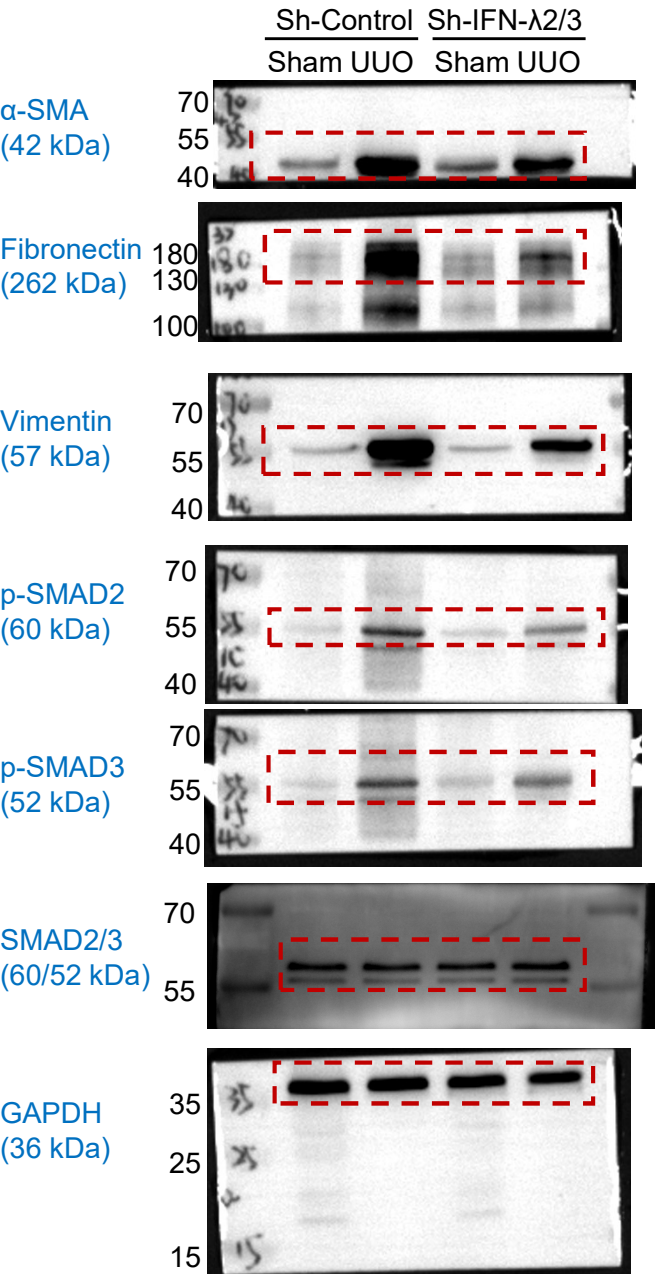

Panel G

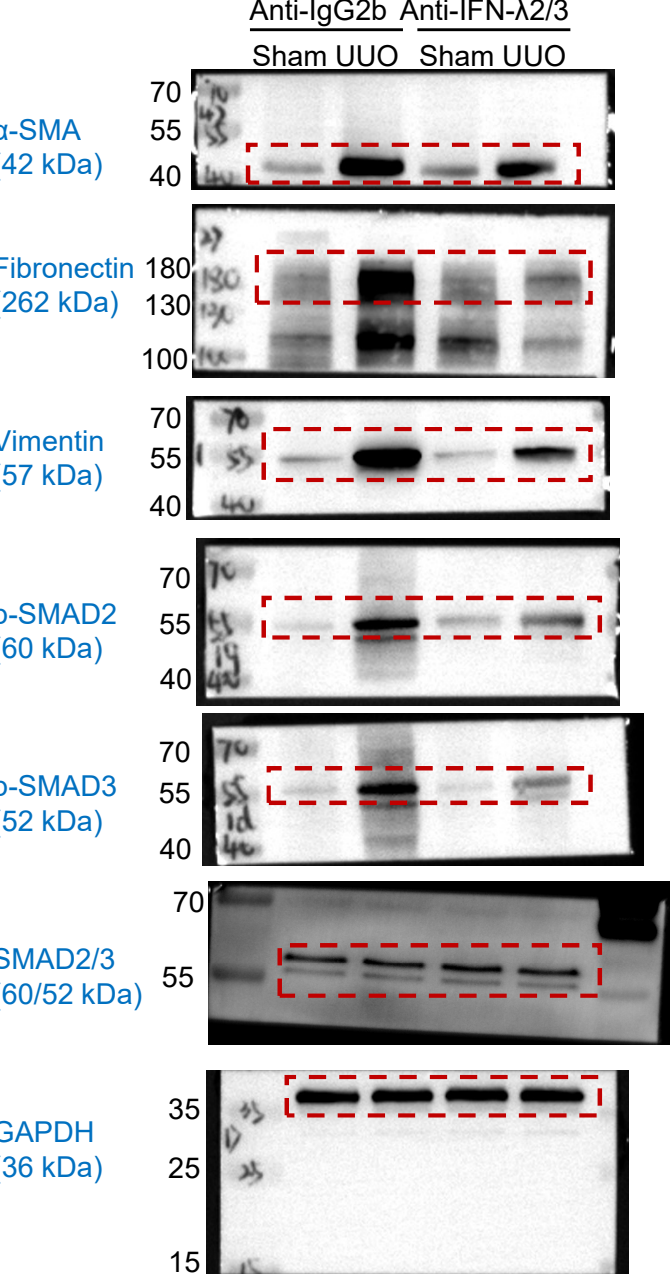

Supplement: SourceData F8 — is the source file for Fig. 8. [file jem_20251858_sourcedataf8.pdf]

Figure S2

Panel D

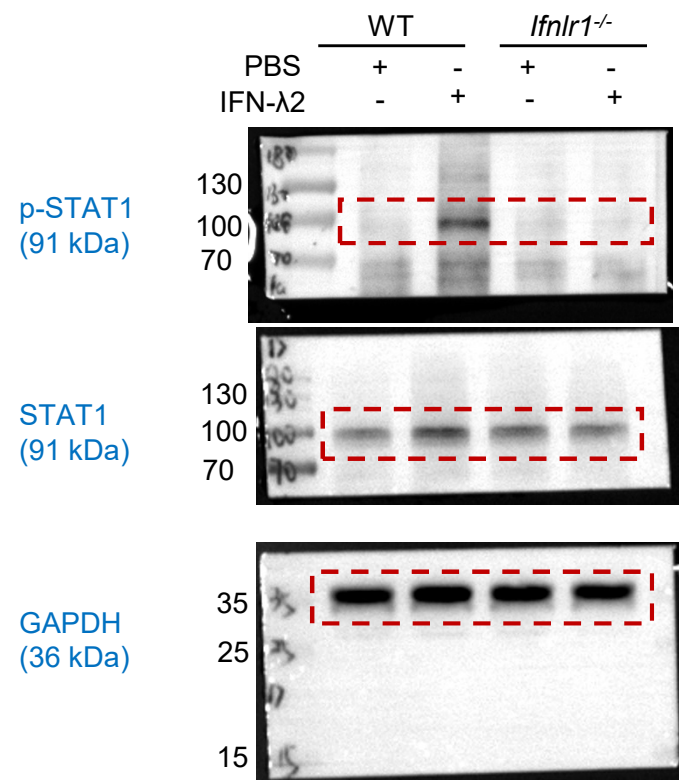

Supplement: SourceData FS2 — is the source file for Fig. S2. [file jem_20251858_sourcedatafs2.pdf]

Figure S3

Panel C

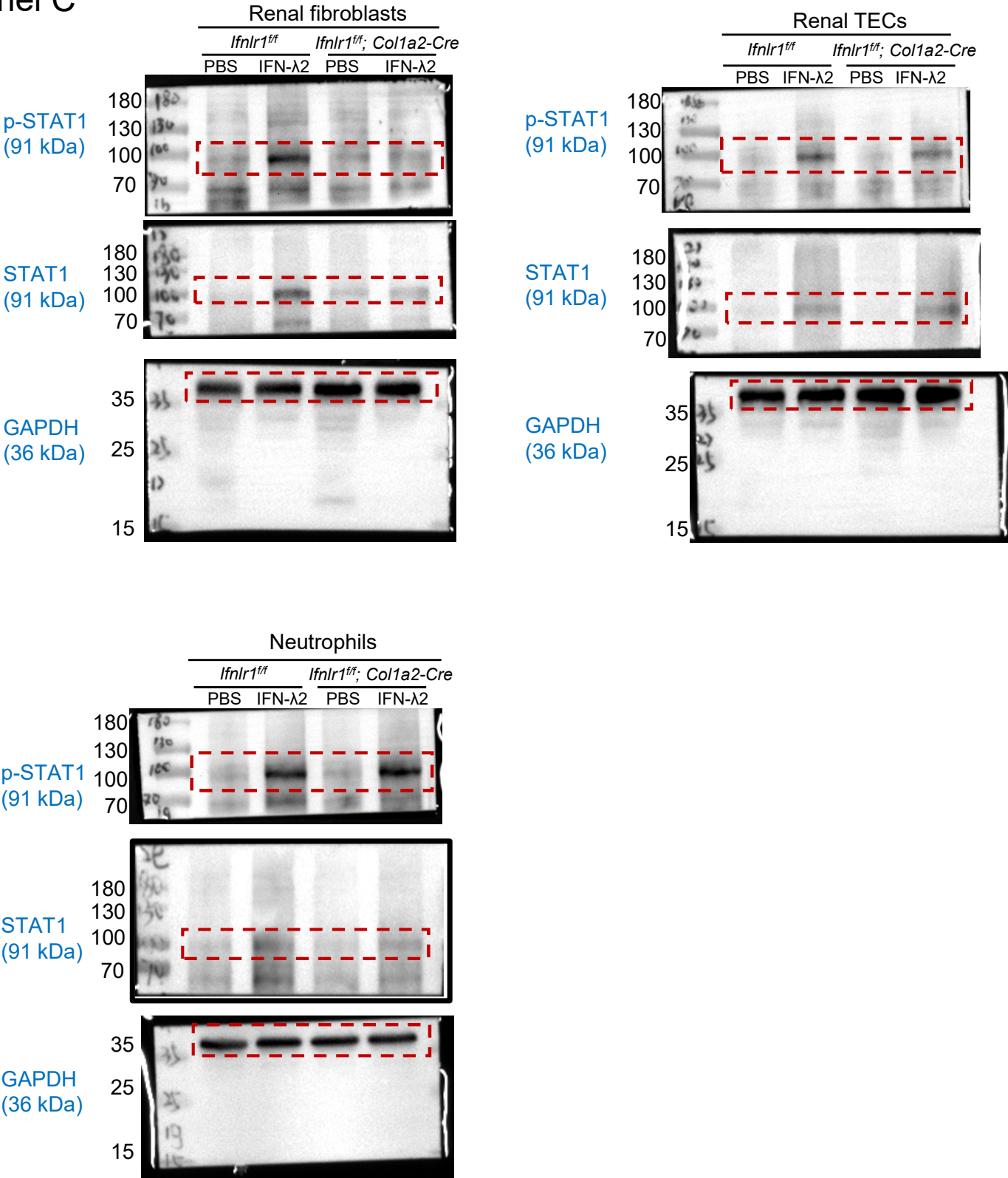

Supplement: SourceData FS3 — is the source file for Fig. S3. [file jem_20251858_sourcedatafs3.pdf]

Figure S4

Panel J

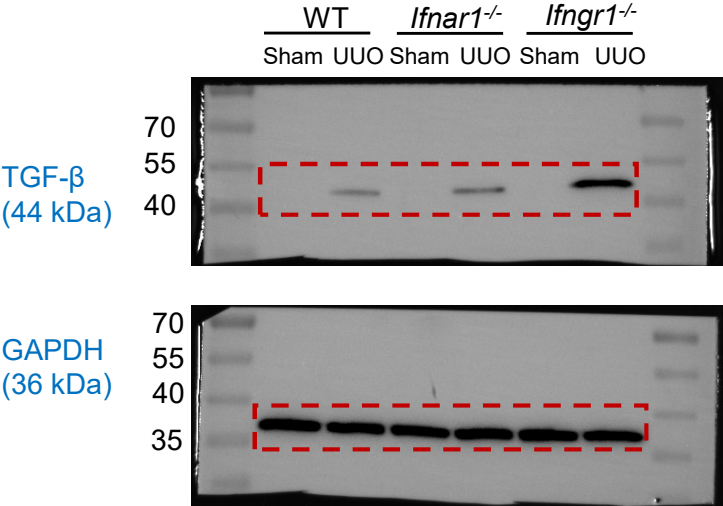

Panel K

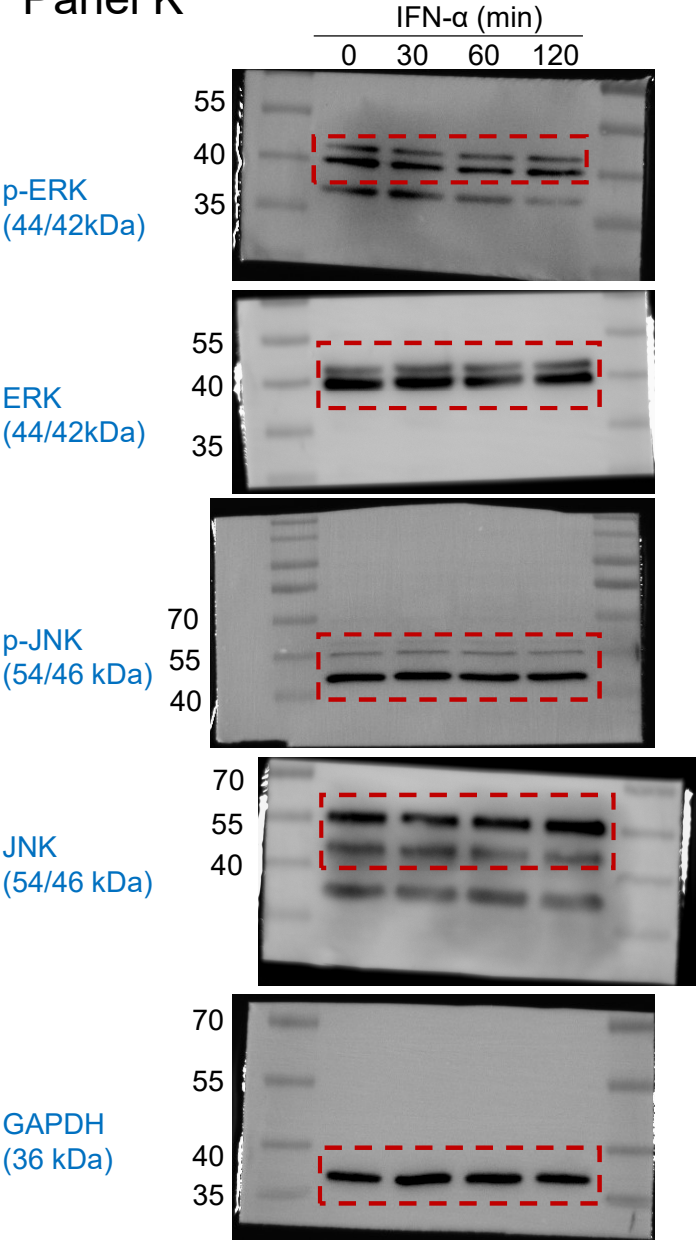

Panel L

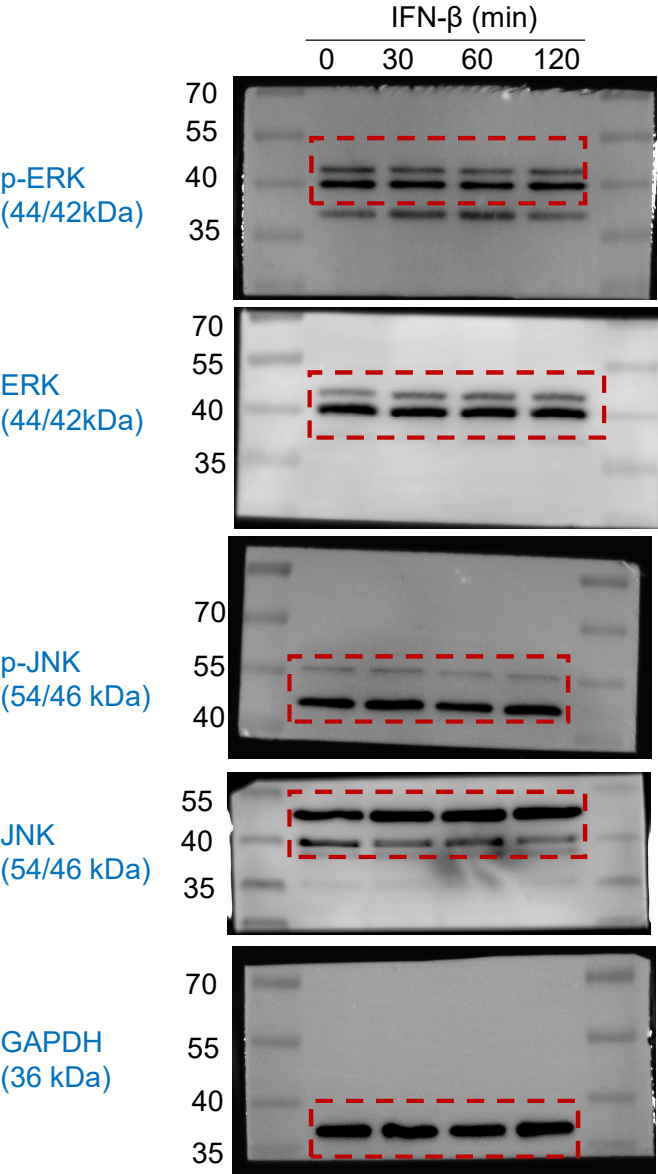

Supplement: SourceData FS4 — is the source file for Fig. S4. [file jem_20251858_sourcedatafs4.pdf]
